# Effectiveness of workplace interventions for health promotion

**DOI:** 10.1016/S2468-2667(25)00095-7

**Published:** 2025-06

**Authors:** Marianna Virtanen, Tea Lallukka, Marko Elovainio, Andrew Steptoe, Mika Kivimäki

**Affiliations:** School of Educational Sciences and Psychology, University of Eastern Finland, Joensuu, Finland; Department of Clinical Neuroscience, Karolinska Institutet, Stockholm, Sweden; Department of Clinical Neuroscience, Karolinska Institutet, Stockholm, Sweden; Department of Public Health, University of Helsinki, Helsinki, Finland; Department of Psychology, University of Helsinki, Helsinki, Finland; Finnish Institute for Health and Welfare, Helsinki, Finland; Department of Behavioural Science and Health, University College London, London, UK; Department of Public Health, University of Helsinki, Helsinki, Finland; Brain Sciences, University College London, London, UK; Finnish Institute of Occupational Health, Helsinki, Finland

## Abstract

Workplaces are an important setting for health promotion, offering established infrastructure, daily access to large populations, and opportunities to engage groups that are often under-represented in such initiatives. Although the effectiveness of workplace health promotion has been evaluated in reviews focusing on specific interventions, a comprehensive overview is needed. To address this gap, we present a quality-informed horizontal analysis encompassing 88 reviews and 339 meta-analysed effect estimates published between 2011 and 2024, covering a broad range of workplace health interventions. Mental health and stress reduction were the most frequently studied targets (36%), followed by weight management and cardiometabolic health (25%), health-related behaviours (22%), and musculoskeletal disorders and pain (17%). According to the GRADE assessment, 71 (21%) of the 339 meta-analysed effect estimates provided evidence of moderate quality, and the remainder were categorised as low or very low quality, with none classified as high quality. Mindfulness showed effectiveness across multiple stress and mental health outcomes, and cognitive behavioural techniques, stress management, physically oriented methods, and e-health interventions also showed some effectiveness. Multicomponent interventions had small but measurable effects on weight loss, glucose levels, fruit intake, and seasonal influenza vaccination uptake. A variety of behavioural, physical activity, environmental, multicomponent, and e-health interventions influenced physical activity and sedentary time at work. Consistent with findings found in non-occupational settings, effects at the individual level were generally modest but could be meaningful at both the workplace and population levels. In this Review we also discuss the broader public health implications of workplace health promotion, and highlight the strengths and limitations of the existing evidence and propose directions for future research.

## Introduction

Work stress, mental health problems, musculoskeletal disorders, and cardiometabolic risks are major public health concerns and, among employees, they contribute to absenteeism, reduced productivity, and increased health-care costs for employees, employers, and society.^1–3^ These risks are influenced by workplace factors.^4–6^ For example, nearly 40% of workers in the EU spend most of their working hours seated, with 18% sitting for more than 7·5 h per day,^7^ and in the USA, 80% of jobs are classified as sedentary or physically light.^8^ Additionally, up to 60% of daily food intake occurs during working hours.^9^ Given these realities, workplaces present a strategic setting for health promotion, offering established infrastructure, daily access to large populations, and the opportunity to engage groups that are often under-represented in such initiatives.

Workplace health promotion refers to the collective efforts of employers, employees, and society to improve the health and wellbeing of people at work.^10^ Individual and group level workplace health promotion, such as wellness programmes, typically includes interventions targeting lifestyle, stress, and mental health. Environment-focused interventions recognise that decision making is not always rational but is influenced by emotions and how choices are presented and framed.^11^ Nudging is a strategy that structures choices to steer individuals towards healthier decisions without imposing substantial economic incentives or restricting freedom of choice.^12^ A comprehensive workplace health promotion policy, along with supportive workplace culture that encourages active employee participation, is an important part of health promotion.^10^

In this Review, we first provide a brief overview of the background and content of workplace health promotion initiatives and previous evidence from umbrella reviews. We then present a systematic horizontal review of quantitative meta-analyses on the effectiveness of these interventions, published between 2011 and 2024, with a focus on interventions targeting stress reduction, mental health, cardiometabolic health, health-related behaviours, and musculoskeletal disorders.

## The workplace as a setting for health promotion

### Interventions targeting health-related behaviours and physical health

Workplace interventions can aim to improve overall wellbeing or promote behavioural changes, such as reducing sedentariness, encouraging a healthy diet, or supporting smoking cessation. Strategies to increase physical activity in the workplace have included behavioural approaches (eg, education and counselling), environmental modifications (eg, sit–stand workstations, treadmill desks, and environmental changes to promote movement), and multicomponent interventions. Umbrella reviews suggest that environmental interventions, such as activity permissive workstations, can reduce sitting time by 40–100 min per 8-h workday.^13,14^ However, evidence for multicomponent interventions remains mixed^13–16^ and findings on e-health interventions using digital and mobile technologies for health promotion have been inconclusive.^17^

Workplace dietary interventions aim to increase fruit and vegetable consumption while reducing sugar, fat, and salt intake.^18^ These interventions use various individual, group, and environmental strategies, such as increasing access to healthy options in the workplace cafeteria; reducing plate, portion, and package sizes; and using point-of-purchase prompts such as motivational messages, posters, and so-called traffic light labels to indicate food and beverage healthiness.^19,20^ Additional strategies include marketing materials, price reductions, and menu modifications to encourage healthier choices in workplace cafeterias.^21^ Previous umbrella reviews on dietary interventions concluded that workplace inter ventions could have small, positive, short-term effects by increasing fruit and vegetable intake,^22,23^ that multicomponent rather than single-component interventions could be effective in improving diet,^15,16^ and they could also improve dietary behaviours.^17^

Many countries have legislation that bans smoking in indoor public places, including workplaces.^24^ However, the prevalence of smoking is still high, especially in low-income and middle-income countries (LMICs).^25^ In the workplace setting, smoking cessation interventions typically include group meetings, individual counselling, educational material, and nicotine replacement therapy.^26^ A 2013 review suggested that all these methods are effective although smoking cessation groups could be particularly effective, with 18% quit rates compared with 12% for other methods.^27^ A recent umbrella review also suggests that multicomponent interventions might be effective.^16^

The costs of alcohol problems in workplaces are substantial, contributing to increased absenteeism, presenteeism (working while ill, with reduced productivity), and workplace accident rates.^3,28^ As a result, prevention of alcohol and other substance misuse is increasingly being integrated in workplace health promotion policies. These interventions include employee education, screening with brief interventions (such as motivational interviewing), psychosocial interventions, drug testing, and employee assistance programmes. A recent umbrella review suggests that motivational interviewing and broad health promotion activities are promising approaches for reducing harmful alcohol use.^16^

Examples of other interventions on health-related behaviours include promotion of seasonal influenza vaccination uptake and breastfeeding. In the workplace setting, policies to enhance vaccination uptake have included education (eg, lectures, group discussions, and online learning), promotional activities (eg, providing the vaccination free of charge, websites, leaflets, and posters) and environmental modifications, such as subtle suggestions without coercion.^29^ In countries where maternity leave is short, early return to work is one of the greatest barriers for women to breastfeed. Tomori and colleagues found that intervention studies on the work place as a setting to promote breastfeeding are scarce.^30^

The workplace can also be a setting for the prevention and management of chronic physical conditions. As fewer than 10% of people with newly diagnosed diabetes attend evidence-based diabetes self-management programmes within the first year of the disease,^31^ the workplace presents a potential setting in which to address common barriers to participation, such as insufficient time, inconvenient location, and lack of awareness of such programmes. Workplace interventions targeting cardiometabolic risk factors—including overweight, high blood pressure, and elevated concentrations of blood glucose and lipids—have typically focused on comprehensive lifestyle modifications. These modifications involve dietary changes, such as reducing saturated fat and trans-fat intake and increasing fibre, fruit, and vegetable intake. Smoking cessation and increased physical activity are also common components of health promotion initiatives.^32^ Previous umbrella reviews suggest that workplace diet and physical activity interventions can be effective in addressing cardiometabolic risk factors, although they typically lead to only modest weight loss;^15,16^ multicomponent interventions appear to yield better results.^15,16^ Another umbrella review found that multi component interventions combining education, dietary modifications, and physical activity were effective in reducing the risk of type 2 diabetes.^33^

Musculoskeletal disorders affect several occupational groups, including office workers, nurses, and dentists, with neck pain prevalence exceeding 50% in these populations.^34^ Among office workers, prolonged sitting and static, unhealthy postures are primary causes of musculoskeletal pain, whereas for nurses and manual workers, physically strenuous tasks—such as lifting, carrying, repetitive movements, and constrained postures—are key contributors.^35,36^ Despite efforts to reduce work-related musculoskeletal workload, the prevalence of musculoskeletal pain has increased.^37^ Musculoskeletal pain often becomes chronic, with 25–33% of patients with lower back pain still having pain after a year.^38^ Many workplace interventions adopt a multilevel approach to the prevention and management of musculoskeletal disorders, following a framework such as the International Classification of Functioning, Disability and Health, which considers individual, environmental and contextual factors as determinants of musculoskeletal disorders and functioning.^39^ A 2018 overview of reviews suggested that workplace modifications, assistive devices, educational interventions (eg, so-called back schools and education on material handling techniques) and exercise (with or without education) could be effective in preventing back pain.^40^

### Interventions that promote mental health

There is growing concern about stress and mental health issues in working populations. Work-related stress, anxiety, and depression are common, with 27–45% of workers in Europe reporting these symptoms.^41,42^ Global estimates suggest that 970 million people have an episode of a mental disorder every year, with over 80% of these cases occurring among the working-age population.^43^ Occupational burnout refers to symptoms that are caused by chronic stress at work and include emotional exhaustion, depersonalisation, and diminished personal accomplishments at work.^44^ Workers in human service occupations, such as nurses and physicians, are at high risk of occupational burnout.^45,46^ Interventions in the workplace context are often group-based and include mindfulness and cognitive behavioural therapy (CBT)-based interventions, relaxation, stress management, resilience training, exercise, and multicomponent interventions. Some umbrella reviews suggest positive or mixed effects of these interventions on various mental health outcomes in the general working population,^47–50^ and in health-care personnel,^51–54^ although the findings for first responders (professionals arriving first at emergency situations)^55^ and for critical situation management^56^ were inconclusive. Other umbrella reviews suggest positive and mixed effects of mindfulness among the general working population^57–59^ and in health-care personnel.^59–62^

Although the research on workplace health promotion has rapidly accumulated over recent decades, the summaries and overviews have repeatedly concluded that limitations exist. Behavioural, non-clinical intervention studies typically suffer from moderate to high risk of bias^63^ and many meta-analyses report high heterogeneity, which make the overall knowledge of the evidence unclear and inconsistent. Despite the abundancy of systematic and umbrella reviews, a comprehensive, quality-informed horizontal analysis—which examines evidence across multiple outcomes while integrating a uniform quality assessment—is needed. Such an approach provides broader insights into the current state of knowledge and can guide employers, workplaces, researchers, clinicians, and policy makers on the effectiveness of workplace interventions.

## A quality-informed horizontal analysis on effectiveness

### Overview of existing meta-analyses by intervention aim, target population, and time

As part of this Review, we conducted a systematic horizontal review of previous meta-analyses to evaluate the effectiveness of a wide range of workplace health promotion interventions. Using explicit criteria for relevance and bias assessment, we synthesised evidence on interventions directly aimed at health promotion, such as workplace wellness programmes, and excluded those interventions primarily focused on medical treatment, the psychosocial work environment, work scheduling, work arrangements, management, and return to work. We covered individual level and group level health promotion interventions targeting key public health challenges, including health-related behaviours, cardiometabolic health, musculoskeletal health, mental health, and stress. Additionally, we included interventions that promote healthy choices through environmental design.

**Figure 1:**
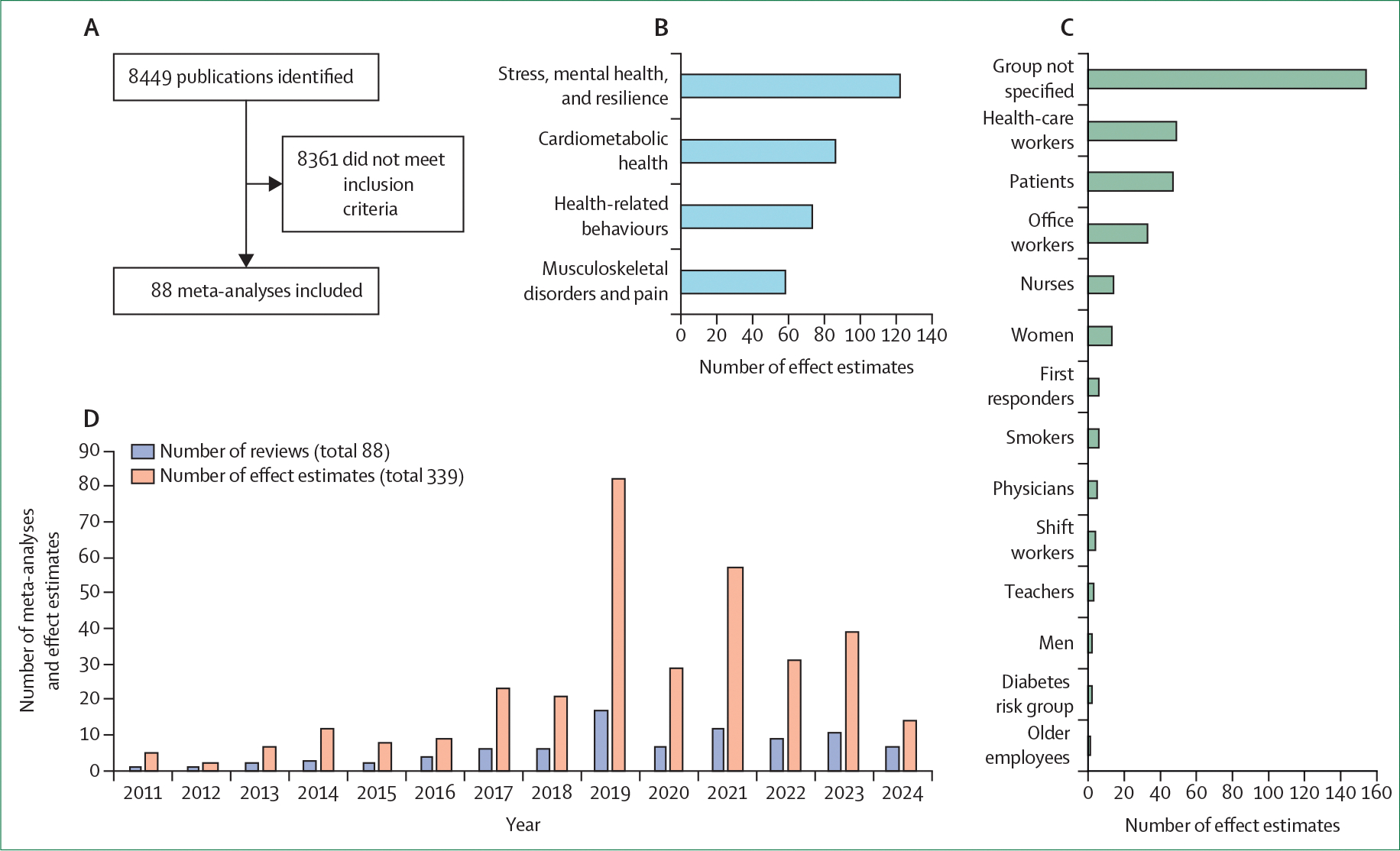
Review of meta-analyses on workplace health promotion interventions by target, participants, and publication year

**Figure 2:**
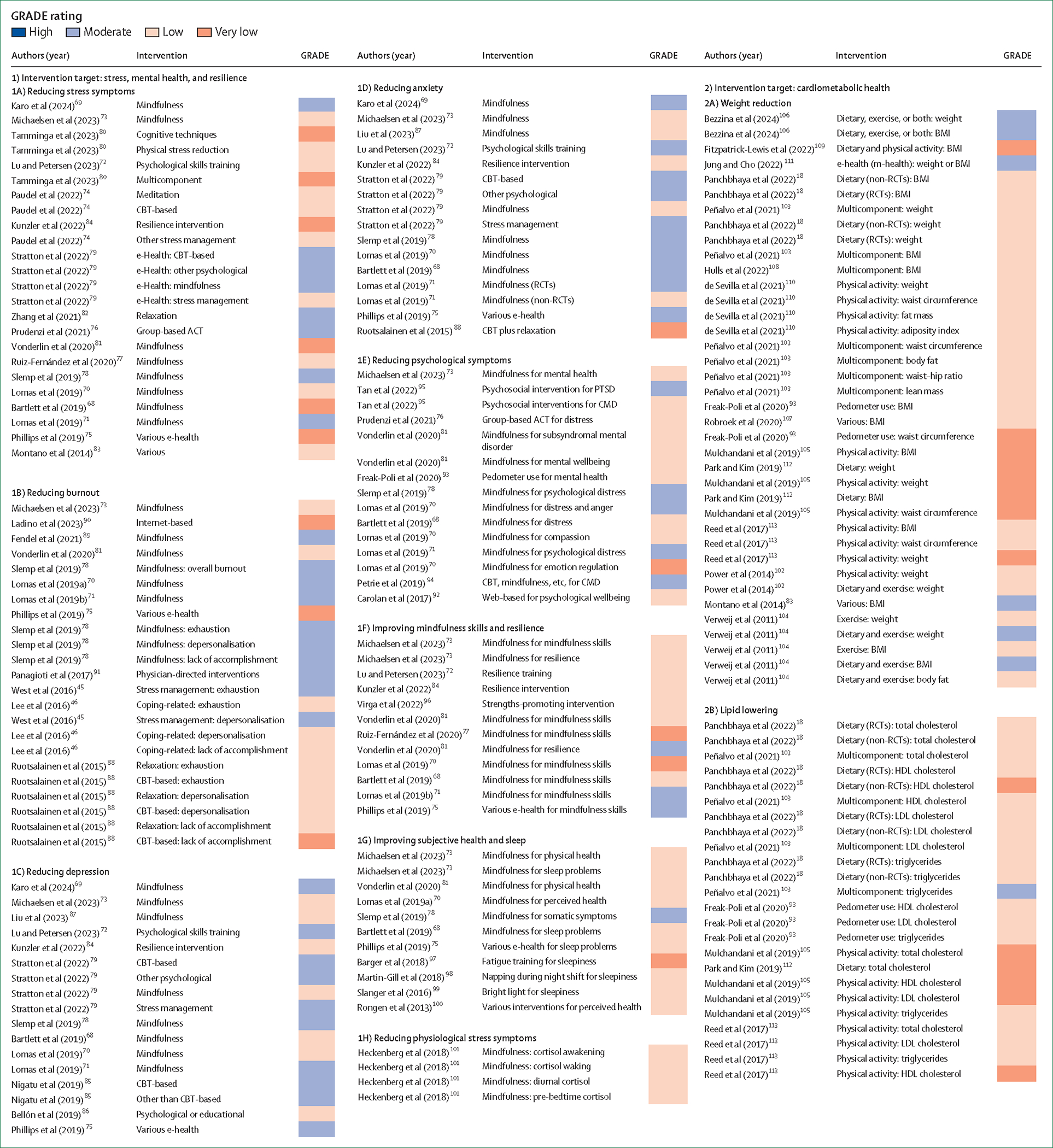

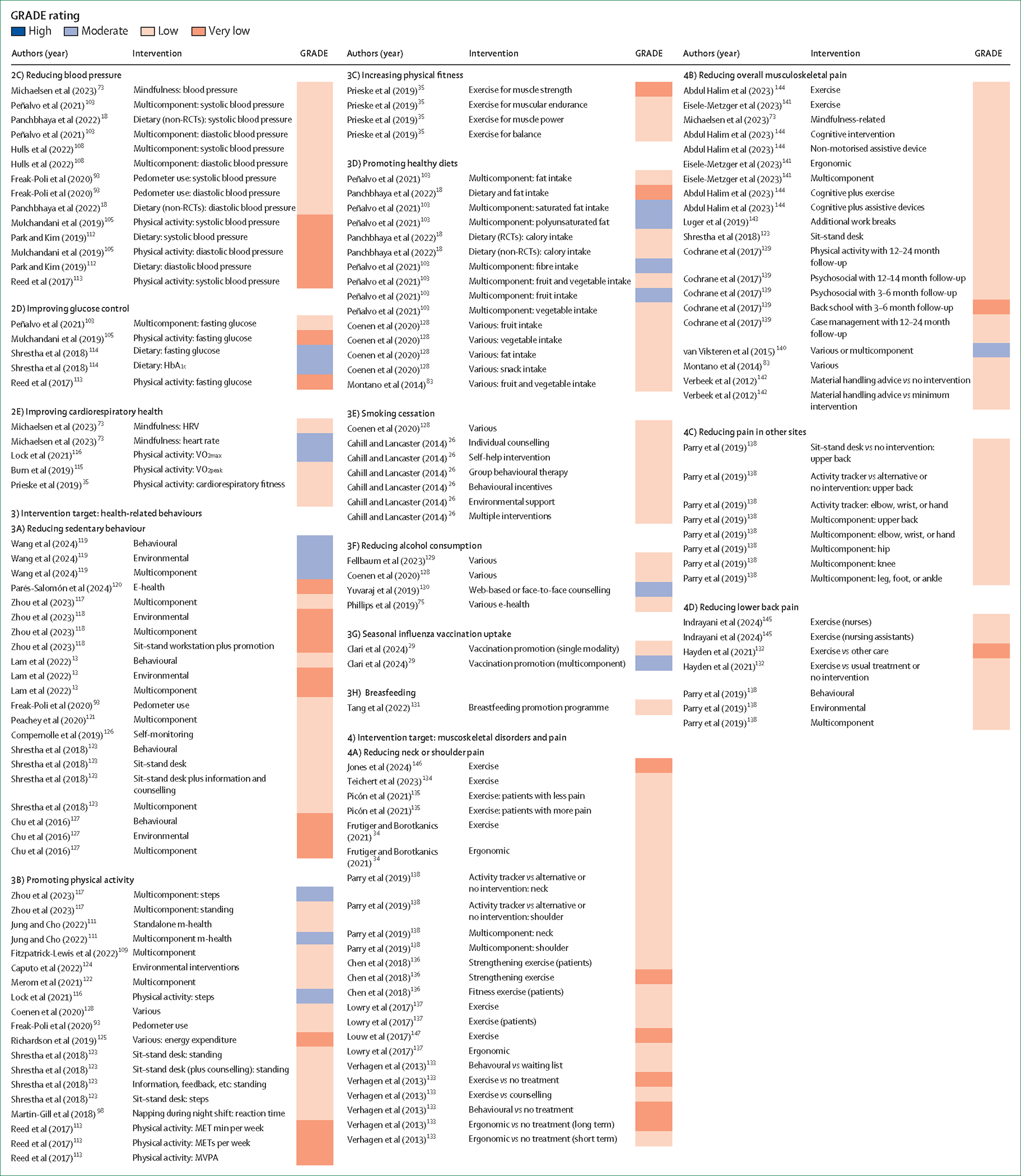
GRADE-based quality of the 339 retrieved meta-analysed effect estimates from 88 meta-analytic studies on the effectiveness of workplace health promotion interventions ACT=acceptance and commitment therapy. CMD=common mental disorder. CBT=cognitive-behavioural therapy. GRADE=Grading of Recommendations, Assessment, Development, and Evaluations. HbA_1c_=glycated haemoglobin. HRV=heart rate variability. MET=metabolic equivalent. MVPA=moderate-to-vigorous physical activity. PTSD=post-traumatic stress disorder. RCT=randomised controlled trial. VO_2_=oxygen consumption.

We followed established guidelines and methods for conducting a systematic review of reviews,^64,65^ including defining inclusion and exclusion criteria, developing a search strategy, selecting studies, extracting data, and presenting results in an appropriate format (figures 1, 2, and appendix pp 1–4). To summarise the quality of evidence for each intervention and outcome estimate, we applied the GRADE framework.^66^ Following this grading, we assessed three key sources of bias: risk of bias in original intervention studies (as reported by the authors in each meta-analysis; appendix pp 5–15), inconsistency (heterogeneity between studies, with *I*^2^ ≥75% indicating high heterogeneity), and small-study effects (evaluated via Egger’s test for publication bias or unknown bias; tables 1, 2). An AMSTAR 2^67^ score was calculated for each meta-analysis.

Of the 8449 publications identified, 88 meta-analyses published between 2011 and 2024 met the inclusion criteria, providing a total of 339 meta-analysed effect estimates (figure 1A, appendix pp 4–15). The interventions examined in these reviews fell into the following broad categories: physical activity (individual and group based physical exercise programmes), dietary (individual and group based programmes for dietary changes), other behavioural interventions (eg, education, information provision, goal setting, feedback, and self-monitoring on various health behaviours), psychological (eg, counselling, self-help, cognitive behavioural based, and resilience inter ventions), mindfulness-based, environmental (eg, ergo nomics, sit–stand workstations, treadmill desks, and environmental changes to promote movement and healthy diet), e-health-based, multi component (combining multiple intervention approaches), and various interventions (a single effect estimate provided for multiple different interventions).

The most common intervention goals were stress reduction and promotion of mental health (accounting for 36% of the effect estimates), followed by improving cardiometabolic health (25%); health-related behaviours (22%); and prevention of musculoskeletal disorders and pain (17%; figure 1B). Although most interventions did not focus on a specific occupational group, several did focus specifically on health-care workers, employees with pre-existing health conditions, and office workers (figure 1C). Most reviews were published between 2019 and 2024 (figure 1D).

Most of the meta-analyses provided estimates for several outcomes. 34 meta-analyses examined interventions for stress reduction and promotion of mental health, covering stress (n [total number of meta-analyses]=17),^68–84^ depression (n=13),^68–73,75,78,79,84–87^ anxiety (n=12),^68–73,75,78,79,84,87,88^ burnout (n=12),^45,46,70,71,73,75,78,81,88–91^ psychological symptoms or mental health (n=11),^68,70,71,73,76,78,81,92–95^ mindfulness skills or resilience (n=9),^68,70–73,75,81,84,96^ sleep (n=6),^68,73,75,97–99^ subjective health or somatic symptoms (n=5),^70,73,78,81,100^ and physiological stress markers (n=1).^101^ For cardiometabolic outcomes, 15 meta-analyses focused on weight management,^18,83,93,102–113^ and others examined interventions targeting blood pressure control (n=8),^18,73,93,103,105,108,112,113^ lipid levels (n=6),^18,93,103,105,112,113^ type 2 diabetes prevention or management (n=4),^103,105,113,114^ cardiometabolic fitness—as measured by, for example, oxygen consumption (VO_2_)_peak_ levels—(n=3),^35,115,116^ and improved cardiovascular or autonomic nervous system function as indicated by heart rate and heart rate variability (n=1).^73^

Regarding workplace interventions aimed at promoting health-related behaviours, we identified 19 meta-analyses targeting sedentary behaviour and physical activity,^13,35,93,98,109,111,113,116–127^ four addressing unhealthy dietary behaviours,^18,83,103,128^ four focusing on harmful alcohol consumption,^75,128–130^ two on smoking cessation,^26,128^ one on seasonal influenza vaccination uptake among health-care workers,^29^ and one on breastfeeding support in the workplace.^131^ Finally, 20 meta-analyses^34,73,83,123,132–147^ examined interventions aimed at reducing musculo skeletal disorders and pain. Detailed information on these meta-analyses is available in the appendix (pp 5–15), and a reference list of 240 systematic reviews without meta-analysis found in our searches (appendix p 37).

### Quality-informed evaluation

We applied the GRADE approach (which assigns ratings, of high, moderate, low, or very low) for quality evaluation for the 339 effect estimates. Among all the 88 meta-analyses reporting these effect estimates, more than 40% of the original studies had a high risk of author-reported bias, preventing us from assigning a GRADE rating of high; therefore, the highest possible rating was moderate (appendix p 1). We further assessed two additional GRADE aspects within the 339 effect estimates: inconsistency (ie, heterogeneity) and publication bias (ie, small-study bias). If neither was present, the rating remained as moderate. The presence of one bias resulted in a downgrade to low, and the presence of both led to a very low rating. Of the 339 estimates, 71 (21%) provided moderate-quality evidence. Based on the GRADE ratings, these 71 effect estimates with moderate-quality evidence are shown in tables 1 and 2, representing the most robust evidence on the effectiveness of workplace health promotion. In addition, nine estimates from three meta-analyses with low grading were included, because they were based on the largest recent meta-analyses on their respective research fields.^73,80,103^ These three meta-analyses, with a larger number of studies but unexplained heterogeneity, might include research from more diverse populations, settings, interventions, and methodologies, which can lead to greater heterogeneity in study-specific estimates—a factor penalised in the GRADE system. Furthermore, regarding mindfulness interventions, a comparison of individual trial overlap between the largest meta-analysis^73^ and the other meta-analyses suggests that the largest meta-analysis had broader inclusion criteria, which can partly explain the observed heterogeneity (appendix pp 23–26).

### Effectiveness of interventions to reduce stress and promote mental health

The evidence on stress and mental health (table 1) graded as moderate quality suggests that mindfulness, CBT-based, and other psychological methods reduce stress, depression, and anxiety symptoms, whether delivered traditionally or via e-health platforms. Mindfulness interventions were also effective in reducing burnout symptoms, improving subjective health and resilience, and enhancing mindfulness skills. Physical stress reduction techniques, such as relaxation exercises, were also effective in reducing stress symptoms, and psychological skills training could prevent depression and anxiety in police personnel. However, group based acceptance and commitment therapy did not reduce stress symptoms among health-care workers, and psychological interventions did not prevent post-traumatic stress disorder among first responders. Effect sizes of these interventions were generally small to moderate, with the exception of mindfulness and e-health, which showed some stronger effects on stress, burnout, and anxiety relief (standardised mean differences of 0·72, 0·70, and 0·79, respectively).

### Effectiveness of interventions on cardiometabolic and musculoskeletal health

The moderate-quality evidence on cardiovascular and musculoskeletal health (table 2) suggests that multicomponent interventions could lead to modest weight loss (0·52–1·19 kg or 0·12–0·34 BMI units), whereas e-health interventions showed no effect. Integration of both individually and environmentally focused interventions could aid in the prevention and management of type 2 diabetes, as indicated by glycated haemoglobin levels.^114^ Multicomponent interventions were not effective in improving blood triglyceride levels. One meta-analysis reported that physical activity interventions effectively improved cardiorespiratory fitness.^116^ Mindfulness practices could enhance cardiovascular or autonomic functioning, as indicated by heart rate.^73^ One meta-analysis suggested that workplace-oriented inter ventions, compared with usual care, had a small effect on musculo skeletal pain for workers on sick leave.^140^

### Effectiveness of interventions promoting health-related behaviours

The evidence suggests that workplace environmental interventions can reduce sedentary time at work by approximately 64 min per day, whereas behavioural interventions can cause reductions of up to 28 min, and the average reduction for multicomponent interventions was 41 min (table 2). Reviews of physical activity programmes, as part of a larger wellness programme or including specific components (eg, self-monitoring devices) and interventions including e-health approaches, suggest improvements in employees’ overall physical activity. We found no evidence for multicomponent interventions when the outcome was overall physical activity. Multi component interventions proved successful in increasing seasonal vaccination uptake among health-care personnel. Regarding dietary behaviour, multi component inter ventions led to a modest increase in fruit consumption (a fifth portion’s increase), but had no effect on fibre intake, saturated fat intake, or polyunsaturated fat intake. Work place interventions had a small effect on alcohol consumption.

### A new era in health promotion: e-health and mobile-health interventions

The COVID-19 pandemic has transformed work arrangements, leading to a rapid rise in remote and hybrid working. In the EU, 13·5% of employees now work from home, with metropolitan areas in Sweden, Ireland, and Finland reporting the highest rates (35–40%).^148^ This shift necessitates a deeper understanding of its health implications, including effects on workplace cohesion, and requires organisations to adapt to the evolving work environment.^149^ As traditional workplace health promotion activities are tied to physical workplaces, e-health is a potential novel pathway to health promotion, as the widespread availability of digital solutions presents an opportunity to extend health promotion beyond the workplace and reach those working from home. Three meta-analyses with moderate GRADE ratings focused on e-health and m-health (ie, delivered via mobile apps) solutions^75,79,111^ and provided small-to-strong effects on depressive, anxiety and stress symptoms, and physical activity, whereas there was no support for their effectiveness in weight management. Although e-health and m-health solutions are low-cost and easy to access, their suitability as a sole intervention must be addressed (ie, whether they can replace face-to-face contacts or whether they work better when used as a complementary method to other forms of workplace health promotion).

### Overall evaluation of effectiveness

All the evidence on workplace intervention effects was first rated as moderate by GRADE, as a large number of the original intervention studies were subject to potential bias. However, in some cases, inconsistency (ie, heterogeneity in effect estimates across trials) and small-study effects (ie, publication bias) were avoided. Since 2019, there was a surge in research on mindfulness-based workplace interventions beyond its original use in stress reduction, cognitive therapy, addiction treatment, and recovery in clinical settings.^59^ Our findings support this approach, as a high AMSTAR-2 quality rating and the most consistent effects across various psychological outcomes and occupational groups related to mind fulness interventions. The most comprehensive and recent meta-analysis—covering 59 studies on stress reduction, 39 on resilience, 25 on somatic symptom relief, 24 on burnout reduction, and 22 on depression prevention—reported unexplained heterogeneity in intervention effects, unlike earlier, smaller meta-analyses.^73^ As a result, this evidence was rated as low quality in GRADE, indicating uncertainty in intervention success. However, evidence from another meta-analysis with 54 studies without heterogeneity confirms that mindfulness interventions could effectively reduce psychological symptoms.^78^ The psychological mechan isms underlying the effectiveness of mindfulness include reduced rumination, improved emotion regulation, and increased cognitive flexibility.^150^ Other forms, such as CBT-based approaches, were also effective in mental health promotion. CBT can enable people to gain control over their thoughts and learn coping skills to manage symptoms.^151^ However, further research is needed to identify the key factors for successful implementation and optimal content of workplace mental health interventions.

For workplace interventions targeting health behaviours and cardiometabolic health, multicomponent approaches were suggested to have a small effect on weight management and fruit intake. However, the findings for glucose control and physical activity were mixed. Multicomponent interventions were also found to increase seasonal influenza vaccination uptake among health-care personnel. The holistic nature of multicomponent interventions offers flexibility, allowing participants to choose the most suitable elements. A domain-specific analysis by Peñalvo and colleagues^103^ suggested that combining group education with food environment modifications, such as nudging, could be particularly effective in increasing fruit intake. However, multicomponent interventions do not always outperform single-component interventions, for example when the goal is to increase physical activity. For interventions addressing harmful alcohol use, workplace interventions had a small effect on people with higher consumption levels (ie, baseline alcohol consumption of over 15 standard drinks per week).^130^ Evidence on the effective types of alcohol interventions and those targeting musculoskeletal disorders and pain remains unclear, as the relevant reviews combined heterogeneous interventions within the same meta-analysed effect estimate. Although the interventions substantially reduced workplace sedentary time, whether this alone improves population health remains uncertain.^152^ The key challenge is ensuring that reduced sedentary time is combined with increased physical activity, particularly moderate-to-vigorous physical activity, which has been emphasised in new guidelines.^152^

With the exception of some stress and mental health interventions, the effect sizes of interventions were modest. Potential reasons include inadequate implementation, external influences from non-work sources such as leisure time,^20^ and the possibility that interventions were too light and short in duration. Several barriers to active participation could also limit effectiveness, including lack of time, scheduling conflicts, low self-efficacy, and low motivation.^153^ For example, effective weight management interventions could require a nutrition component and at least 135 min of supervised moderate-intensity exercise per week for a minimum of 4–6 months.^110^ For certain groups, such as night workers, those with long working hours, or employees with irregular schedules, workplace interventions could be particularly effective, a topic needing further investigation. Furthermore, in primary prevention, most individuals remain healthy, meaning that expected intervention effects are inherently small. However, even small improvements at the population level can have important public health benefits.^47^

## Workplace interventions, socioeconomic health inequalities, and controversies

Workplaces can complement public health efforts led by primary health care and health-care policies. However, as workplace interventions target only employed individuals, they exclude the unemployed, those retired, and other people outside the labour market, including due to health reasons. This selective reach raises concerns that workplace health promotion could contribute to socioeconomic health inequalities.

Our analysis identified three meta-analyses addressing socioeconomic health inequalities between employees.^83,107,128^ One found that intervention studies were more than twice as common among workers with higher socioeconomic position than those with lower socioeconomic position, particularly in research focusing on musculoskeletal disorders and work stress.^83^ Similarly, many physical activity interventions in our review targeted office workers rather than manual labourers. However, two other meta-analyses from the same dataset suggested that workplace interventions do not necessarily exacerbate socioeconomic inequalities within the working population, because the effects did not differ between socioeconomic groups.^107,128^ A 2020 systematic review on this issue had similar conclusions.^154^

The systematic review by Sundstrup and colleagues^155^ found that workplace interventions might reduce musculoskeletal disorders in physically demanding jobs, whereas the review by Crane and colleagues^156^ suggested that for workers with low socioeconomic position, tailoring interventions to specific work settings and individual contact could improve effectiveness. Another review examined workplace interventions in LMICs,^157^ but identified only three successful workplace interventions, with overall study quality rated as poor. These findings highlight that individuals with low socioeconomic position and workers in LMICs remain under-represented in workplace intervention research, leaving open the question of whether workplace health promotion reduces or increases socioeconomic inequalities in employee health.^158^

There are also notable controversies in health promotion. Traditional approaches assume that individuals are reflective, goal-oriented, and guided by their definite value system, with interventions focusing on providing information, changing attitudes, and motivating behaviour change.^11,12^ An alternative perspective recognises the influence of an automatic, effect-driven system that operates with minimal cognitive involvement, overriding the rational, goal-directed decision making. To address this, the nudging method was developed to leverage environmental cues, making healthier choices more accessible or appealing without imposing major economic incentives or restricting individual freedom.^11,12^ However, in practice, harmful purposes can often outweigh healthy nudging. For example, the ways in which food is marketed, packaged, and displayed tend to trigger automatic emotional responses that encourage overconsumption.^159^ An alternative approach, known as boosting, aims to equip people with the skills or tools needed to make better choices themselves. Unlike nudging, which modifies external influences, boosting focuses on strengthening internal decision-making abilities. However, the effectiveness of boosting needs to be fully explored in future studies.^160^

### Limitations

Our Review has several limitations, including the reliance on author-reported publication bias and study quality. In reviews of reviews, such as ours, the overlap of primary studies across meta-analyses can be a concern. Combining estimates from multiple reviews into a meta-analysis without accounting for this overlap can artificially inflate the sample size, leading to over estimated precision of intervention effects. To avoid this bias, we compared the findings from different meta-analyses qualitatively rather than quantitatively. In addition, we did a brief check of primary studies to get information on the level of overlapping in original studies and meta-analyses (appendix p 23). For example, in the largest and most recent review^73^ on interventions to improve mindfulness (N=39), nearly half (48·7%, N=22) of the original trials were also included in other reviews on the same topic. The second largest review^81^ contained 10 trials that were not part of the most recent review, highlighting differences in inclusion criteria across reviews. We observed lower levels of overlap for other mindfulness-related outcomes, such as stress reduction (32·2% of trials in the most recent review^73^ were included in previous reviews), depression (31·8% overlap), and burnout (41·2% overlap). Despite these variations in overlap, our conclusions remain robust, as the main findings consistently converged across reviews, and since we do not provide quantitative effect size estimates, study overlap is an unlikely source of bias in this Review.

It is also possible that we missed some articles through our searches, although the total number was extensive: 403 articles (including umbrella reviews and reviews with or without meta-analysis). It is also important to note that, as the data are based on published meta-analyses, our findings depend on their quality and do not include topics for which meta-analyses have not yet been published. Lastly, the generalisability of some findings is limited, as certain interventions targeted specific groups, such as health-care workers.

## Conclusions and implications for future research

Our Review found most consistent evidence supporting interventions that were aimed at stress reduction and mental health promotion. Positive effects were also seen in reducing sedentary behaviours and, to a lesser extent, in interventions promoting physical activity and healthier diets. Interventions improving glucose control showed some effectiveness, and there was evidence of increased vaccination uptake among health-care workers. Modest evidence was observed for interventions targeting weight management, reducing harmful drinking, and improving cardiorespiratory health. The evidence for interventions aimed at preventing musculoskeletal disorders and pain was uncertain, as only one meta-analysis remained in the final analytic sample. Our Review reveals substantial limitations in the quality of existing evidence. Of the 339 intervention–outcome pairs (ie, effect estimates), only 71 (21%) were rated as providing moderate-quality evidence, none were rated as high quality, and 268 (79%) were classified as providing only low-quality or very low-quality evidence.

Conducting large, multicentre, randomised controlled trials (RCTs) with long follow-ups in workplace settings is challenging due to heterogeneity of work environments, the special needs of target populations, and the lack of one-size-fits-all solutions.^22^ The role of organisational culture, which likely influences intervention effectiveness, has been largely overlooked in workplace health promotion research.^106^ In addition, the more complex the intervention is, the more thorough the evaluation process can need to be.^161^ Complex interventions—those involving multiple components, diverse target groups, and various experts delivering the intervention—can require more comprehensive evaluation strategies than are applied in the existing research. To address these, broader evaluation frameworks that integrate assessments of efficacy, effectiveness, and underlying theoretical frameworks within a systems approach could be needed for a more in-depth evaluation of workplace health promotion interventions.^161^

Future research should assess whether the workplace is a more effective platform for the implementation of health promotion interventions compared with non-occupational settings. Insights from a Cochrane review and meta-analysis suggest that the effect of physical exercise interventions on chronic low back pain was comparable across clinical, occupational, and general population studies.^132^ Similarly, the effectiveness of occupational smoking cessation interventions mirrored that of interventions in non-occupational settings.^26^ Further research should focus on under-represented populations, including workers with low socioeconomic position, those in LMICs, young workers, shift workers, and those working in small and medium-sized enterprises.^162^ In the EU, many small and medium-sized enterprises lack even basic occupational health and safety management measures, such as risk assessment and monitoring,^163^ highlighting the need for innovative strategies to support their capacity to implement workplace health promotion programmes.^164^

In this Review, we did not evaluate interventions that targeted psychosocial work environment (eg, modification of working conditions [such as work demands, job control, role clarity, effort–reward imbalance, and violence], leadership, workplace climate, and social relationships), shift work schedules, or economic aspects, such as return on investment metrics, productivity, or sickness absence. Future reviews are warranted to evaluate these aspects of workplace health promotion.^165^ Future studies should also examine sensitive aspects of workplace health promotion, including challenges related to disclosing health conditions, concerns about confidentiality, and the risk of unwanted disclosure of health information to colleagues or employers.

In particular, meta-analyses of interventions to prevent musculoskeletal diseases often suffered from small-study effects (publication bias). This bias can be avoided, for example by conducting larger, well-powered intervention studies, by implementing rigorous study designs, and by publishing all study results. For meta-analyses, identifying both published and unpublished data can reduce publication bias. Finally, longer follow-ups are needed to assess the long-term benefits of workplace health promotion. For example, most RCTs in mindfulness had follow-up periods of no more than 12 weeks after the intervention, and the number of trials with extended follow-ups was too small for meta-analysis.^81^ Similarly, there was an insufficient number of RCTs on physical activity interventions for meta-analysis, with follow-ups rarely exceeding 1 year.^116^ The findings of this Review highlight positive effects of some workplace-based health promotion strategies; however, the lack of high-quality intervention studies and the heterogeneity of existing evidence preclude firm conclusions about their overall effectiveness and contribution to public health.

## Supplementary Material

1

## Figures and Tables

**Table 1:** Effectiveness of workplace interventions on stress, mental health, and subjective health, by target and intervention type

	Author (year)	Population	N (studies)	Effect (standard mean difference unless otherwise indicated)—favouring intervention	95% confidence interval	GRADE rating	Risk of bias in studies*	Inconsistency†	Small-study effects‡	Low AMSTAR-2 score	Comment

**Reducing stress symptoms**											
Mindfulness	Michaelsen et al (2023)^73^	Not specified	59	0·72	0·54 to 0·90	Low	Yes	Yes	No	No	The most comprehensive and recent meta-analysis on the topic
Mindfulness	Slemp et al (2019)^78^	Not specified	32	0·47	0·35 to 0·58	Moderate	Yes	No	No	Yes	··
Mindfulness	Lomas et al (2019)^71^	Health-care workers	7	0·42	0·17 to 0·67	Moderate	Yes	No	No	Yes	··
Mindfulness	Karo et al (2024)^69^	Nurses	5	0·50	0·18 to 0·82	Moderate	Yes	No	No	Yes	··
Physical stress reduction	Tamminga et al (2023)^80^	Health-care workers	35	0·55	0·40 to 0·70	Low	Yes	No	Yes	No	The most comprehensive and recent meta-analysis on the topic
Relaxation	Zhang et al (2021)^82^	Health-care workers	15	0·53	0·33 to 0·74	Moderate	Yes	No	No	Yes	··
Group ACT	Prudenzi et al (2021)^76^	Health-care workers	9	0·15	−0·15 to 0·46	Moderate	Yes	No	No	Yes	ACT
E-health	Stratton et al (2022)^79^	Not specified	19	0·10	0·04 to 0·16	Moderate	Yes	No	No	Yes	CBT-based e-health
E-health	Stratton et al (2022)^79^	Not specified	9	0·12	0·04 to 0·21	Moderate	Yes	No	No	Yes	Other psychological methods (eg, cognitive training)
E-health	Stratton et al (2022)^79^	Not specified	15	0·42	0·31 to 0·53	Moderate	Yes	No	No	Yes	Mindfulness-based e-health
**Reducing burnout**											
Mindfulness	Michaelsen et al (2023)^73^	Not specified	24	0·70	0·33 to 1·07	Low	Yes	Yes	No	No	The most comprehensive and recent meta-analysis on the topic
Mindfulness	Fendel et al (2021)^89^	Physicians	5	0·26	0·03 to 0·50	Moderate	Yes	No	No	No	··
Mindfulness	Lomas et al (2019)^70^	Not specified	14	0·36	0·16 to 0·55	Moderate	Yes	No	No	Yes	··
Mindfulness	Lomas et al (2019)^71^	Health-care workers	4	0·31	0·04 to 0·57	Moderate	Yes	No	No	Yes	··
Mindfulness	Slemp et al (2019)^78^	Not specified	24	0·07	−0·06 to 0·20	Moderate	Yes	No	No	Yes	Overall burnout score
Mindfulness	Slemp et al (2019)^78^	Not specified	17	0·29	0·15 to 0·42	Moderate	Yes	No	No	Yes	Emotional exhaustion score
Mindfulness	Slemp et al (2019)^78^	Not specified	13	0·17	0·02 to 0·33	Moderate	Yes	No	No	Yes	Depersonalisation score
Mindfulness	Slemp et al (2019)^78^	Not specified	13	0·23	0·05 to 0·42	Moderate	Yes	No	No	Yes	Lack of accomplishment score
Physician-directed interventions	Panagioti et al (2017)^91^	Physicians	12	0·18	0·03 to 0·32	Moderate	Yes	No	No	Yes	Mindfulness and CBT techniques; outcome: emotional exhaustion
Stress management	West et al (2016)^45^	Physicians	12	2·06	0·27 to 3·86	Moderate	Yes	No	No	Yes	Mean difference (survey score): emotional exhaustion
Stress management	West et al (2016)^45^	Physicians	11	0·92	−0·05 to 1·90	Moderate	Yes	No	No	Yes	Mean difference (survey score): depersonalisation
**Reducing depression**											
Mindfulness	Michaelsen et al (2023)^73^	Not specified	22	0·51	0·29 to 0·73	Low	Yes	Yes	No	No	The most comprehensive and recent meta-analysis on the topic
Mindfulness	Slemp et al (2019)^78^	Not specified	13	0·42	0·24 to 0·59	Moderate	Yes	No	No	Yes	··
Mindfulness	Karo et al (2024)^69^	Nurses	5	0·42	0·06 to 0·78	Moderate	Yes	No	No	Yes	··
Mindfulness	Lomas et al (2019)^71^	Health-care workers	3	0·55	0·22 to 0·87	Moderate	Yes	No	No	Yes	··
CBT-based	Nigatu et al (2019)^85^	Patients	10	0·44	0·26 to 0·61	Moderate	Yes	No	No	Yes	··
Psychological skills training	Lu and Petersen (2023)^72^	Police officers	9	0·47	0·22 to 0·73	Moderate	Yes	No	No	No	··
Other than CBT methods	Nigatu et al (2019)^85^	Patients	6	0·32	0·06 to 0·59	Moderate	Yes	No	No	Yes	Various methods (eg, exercise and stress management)
E-health	Stratton et al (2022)^79^	Not specified	23	0·11	0·06 to 0·17	Moderate	Yes	No	No	Yes	CBT-based e-health
E-health	Stratton et al (2022)^79^	Not specified	13	0·15	0·09 to 0·21	Moderate	Yes	No	No	Yes	Other psychological methods (eg, cognitive training)
E-health	Stratton et al (2022)^79^	Not specified	4	0·61	0·47 to 0·75	Moderate	Yes	No	No	Yes	Stress management
E-health	Phillips et al (2019)^75^	Not specified	17	0·30	0·18 to 0·42	Moderate	Yes	No	No	Yes	Various e-health methods
**Reducing anxiety**											
Mindfulness	Michaelsen et al (2023)^73^	Not specified	23	0·53	0·31 to 0·74	Low	Yes	Yes	No	No	··
Mindfulness	Slemp et al (2019)^78^	Not specified	11	0·58	0·37 to 0·79	Moderate	Yes	No	No	Yes	··
Mindfulness	Karo et al (2024)^69^	Nurses	7	0·06	−0·25 to 0·14	Moderate	Yes	No	No	Yes	··
Mindfulness	Lomas et al (2019)^70^	Not specified	5	0·57	0·73 to 0·81	Moderate	Yes	No	No	Yes	··
Mindfulness	Bartlett et al (2019)^68^	Not specified	4	0·62	0·72 to 0·92	Moderate	Yes	No	No	Yes	··
Mindfulness	Lomas et al (2019)^71^	Health-care workers	3	0·49	0·16 to 0·81	Moderate	Yes	No	No	Yes	··
Psychological skills training	Lu and Petersen (2023)^72^	Police officers	9	0·40	0·06 to 0·73	Moderate	Yes	No	No	No	··
E-health	Phillips et al (2019)^75^	Not specified	15	0·34	0·18 to 0·70	Moderate	Yes	No	No	Yes	Various e-health methods
E-health	Stratton et al (2022)^79^	Not specified	14	0·11	0·04 to 0·19	Moderate	Yes	No	No	Yes	CBT-based e-health
E-health	Stratton et al (2022)^79^	Not specified	6	0·13	0·01 to 0·25	Moderate	Yes	No	No	Yes	Other psychological methods (eg, cognitive training)
E-health	Stratton et al (2022)^79^	Not specified	4	0·79	0·64 to 0·93	Moderate	Yes	No	No	Yes	Stress management
**Reducing psychological symptoms**											
Mindfulness	Slemp et al (2019)^78^	Not specified	54	0·39	0·30 to 0·49	Moderate	Yes	No	No	Yes	··
Mindfulness	Lomas et al (2019)^70^	Not specified	14	0·56	0·41 to 0·72	Moderate	Yes	No	No	Yes	··
Mindfulness	Lomas et al (2019)^71^	Health-care workers	7	0·61	0·44 to 0·79	Moderate	Yes	No	No	Yes	··
Other methods	Tan et al (2022)^95^	First responders	12	0·05	−0·01 to 0·11	Moderate	Yes	No	No	Yes	For PTSD; trauma risk management, stress management, and debriefing
Other methods	Petrie et al (2019)^94^	Physicians	5	0·62	0·40 to 0·83	Moderate	Yes	No	No	Yes	CBT-based, mindfulness, stress management, and social support
**Improving mindfulness skills and resilience**											
Mindfulness	Michaelsen et al (2023)^73^	Not specified	39	0·43	0·33 to 0·52	Low	Yes	Yes	No	No	The most comprehensive and recent meta-analysis; outcome: mindfulness
Mindfulness	Lomas et al (2019)^71^	Health-care workers	5	0·34	−0·06 to 0·73	Moderate	Yes	No	No	Yes	Outcome: mindfulness
Mindfulness	Vonderlin et al (2020)^81^	Not specified	4	0·49	0·74 to 0·73	Moderate	Yes	No	No	Yes	Outcome: resilience
E-health	Phillips et al (2019)^75^	Not specified	5	0·42	0·24 to 0·60	Moderate	Yes	No	No	Yes	Various e-health methods; outcome: mindfulness
**Improving subjective health**											
Mindfulness	Michaelsen et al (2023)^73^	Not specified	25	0·40	0·30 to 0·50	Low	Yes	Yes	No	No	The most comprehensive and recent meta-analysis on the topic
Mindfulness	Slemp et al (2019)^78^	Not specified	4	0·40	0·03 to 0·77	Moderate	Yes	No	No	Yes	··

ACT=acceptance and commitment therapy. CBT=cognitive-behavioural therapy. PTSD=post-traumatic stress disorder.

* High risk of bias in >40% of the original interventions.

† High between-study heterogeneity; *I*^2^ ≥75%.

‡ Detected or unclear bias due to small-study effects.

**Table 2:** Effectiveness of workplace interventions on health behaviours and cardiometabolic health, by target and intervention type

	Author (year)	Population	n (studies)	Effect (standard mean difference unless otherwise indicated)—favouring intervention	(95% confidence interval)	GRADE rating	Risk of bias in studies*	Inconsistency†	Small-study effects‡	Low AMSTAR-2 score^67^	Comment

**Weight reduction (kg)**											
Multicomponent	Peñalvo et al (2021)^103^	Not specified	59	0·52	0·31 to 0·72	Low	Yes	Yes	No	Yes	The most comprehensive and recent meta-analysis on the topic
Dietary, exercise, or both	Bezzina et al (2024)^106^	Male workers	14	1·07	−0·13 to 2·28	Moderate	Yes	No	No	Yes	Dietary, exercise, or both
Multicomponent	Verweij et al (2011)^104^	Not specified	9	1·19	0·74 to 1·64	Moderate	Yes	No	No	Yes	A combination of dietary and exercise intervention
**BMI reduction (units)**											
Multicomponent	Peñalvo et al (2021)^103^	Not specified	67	0·12	0·06 to 0·48	Low	Yes	Yes	No	Yes	The most comprehensive and recent meta-analysis on the topic
Dietary, exercise, or both	Bezzina et al (2024)^106^	Male workers	12	0·27	−0·09 to 0·63	Moderate	Yes	No	No	Yes	Dietary, exercise, or both
Various	Montano et al (2014)^83^	Not specified	12	0·16	0·02 to 0·29	Moderate	Yes	No	No	Yes	Standard mean difference instead of mean difference
Multicomponent	Verweij et al (2011)^104^	Not specified	11	0·34	0·22 to 0·46	Moderate	Yes	No	No	Yes	A combination of dietary and exercise intervention
E-health (m-health)	Jung and Cho (2022)^111^	Not specified	4	0·02	−0·07 to 0·10	Moderate	Yes	No	No	Yes	Weight or BMI: standard mean difference instead of mean difference
**Lipid lowering: triglycerides (mg/dL)**											
Multicomponent	Peñalvo et al (2021)^103^	Not specified	26	2·14	−2·11 to 6·39	Moderate	Yes	No	No	Yes	··
**Improving glucose control: fasting glucose (mg/dL)**											
Multicomponent	Shrestha et al (2018)^114^	Not specified	16	2·60	−0·08 to 5·27	Moderate	Yes	No	No	Yes	Individual and environmental dietary intervention
**Improving glucose control: HbA_1c_ (%)**											
Multicomponent	Shrestha et al (2018)^114^	Not specified	13	0·18	0·06 to 0·29	Moderate	Yes	No	No	Yes	Individual and environmental dietary intervention
**Improving cardiorespiratory health**											
Physical activity	Lock et al (2021)^116^	Not specified	11	2·53	1·69 to 3·36	Moderate	Yes	No	No	Yes	Outcome: cardiorespiratory fitness (VO_2max_)
Mindfulness	Michaelsen et al (2023)^73^	Not specified	6	0·21	0·08 to 0·33	Moderate	Yes	No	No	No	Outcome: heart rate (standard mean difference)
**Reducing sedentary behaviours: min/8-h workday**											
Behavioural	Wang et al (2024)^119^	Office workers	7	27·99	5·78 to 50·22	Moderate	Yes	No	No	Yes	··
Environmental	Wang et al (2024)^119^	Office workers	4	63·68	20·1 to 107·2	Moderate	Yes	No	No	Yes	··
Multicomponent	Wang et al (2024)^119^	Office workers	13	40·74	31·3 to 50·2	Moderate	Yes	No	No	Yes	··
**Promoting physical activity**											
Multicomponent	Zhou et al (2023)^117^	Office workers	8	3·14	−0·19 to 6·47	Moderate	Yes	No	No	Yes	Difference in steps per 8-h workday
Physical activity	Lock et al (2021)^116^	Not specified	16	814·01	446·4 to 1181·7	Moderate	Yes	No	No	Yes	Difference in steps per day
E-health and m-health	Jung and Cho (2022)^111^	Not specified	7	0·19	0·05 to 0·33	Moderate	Yes	No	No	Yes	Standard mean difference in physical activity
**Promoting healthy diets**											
Multicomponent	Peñalvo et al (2021)^103^	Not specified	4	0·31	−0·25 to 0·87	Moderate	Yes	No	No	Yes	Outcome: saturated fat intake (% daily energy)
Multicomponent	Peñalvo et al (2021)^103^	Not specified	3	0·23	−0·13 to 0·59	Moderate	Yes	No	No	Yes	Outcome: polyunsaturated fat intake (% daily energy)
Multicomponent	Peñalvo et al (2021)^103^	Not specified	15	0·20	0·11 to 0·28	Moderate	Yes	No	No	Yes	Outcome: fruit consumption (servings per day)
Multicomponent	Peñalvo et al (2021)^103^	Not specified	15	0·26	−0·15 to 0·67	Moderate	Yes	No	No	Yes	Outcome: fibre intake (g/day)
**Reducing alcohol consumption (units per week)**											
Various	Yuvaraj et al (2019)^130^	Not specified	7	2·25	0·30 to 4·20	Moderate	Yes	No	No	Yes	All interventions merged in the estimate; mean difference (units)
**Seasonal influenza vaccination uptake**											
Multicomponent	Clari etal (2024)^29^	Health-care workers	5	1·37	1·13 to 1·66	Moderate	Yes	No	No	Yes	Risk ratio
**Reducing musculoskeletal pain**											
Various	van Vilsteren et al (2015)^140^	Patients	5	0·26	0·06 to 0·47	Moderate	Yes	No	No	No	All interventions merged in the estimate; standard mean difference

HbA_1c_ =glycated haemoglobin. VO_2_=oxygen consumption.

* High risk of bias in >40% of the original interventions.

† High between-study heterogeneity; *I*^2^ ≥75%.

‡ Detected or unclear bias due to small-study effects.

## Search strategy and selection criteria

We searched PubMed and Web of Science from Jan 1, 2010, to Jan 3, 2025, using search terms such as ”workplace”, ”worksite”, ”wellness programs”, and ”health promotion”, focusing on published systematic reviews with meta-analysis. We also conducted a cited reference search in Web of Science for all identified systematic reviews and meta-analyses. Inclusion criteria were: (1) systematic reviews with meta-analysis including at least two randomised controlled trials; (2) focus on workplace health promotion; (3) interventions targeting at least one of promotion of health-related behaviours, improvement of cardiometabolic health (eg, prevention of type 2 diabetes, cardiovascular diseases, and change in blood biomarkers), prevention of musculoskeletal disorders, or stress reduction and mental health promotion; (4) publication in a peer-reviewed international scientific journal (non-predatory) from 2010 onwards; (5) not focused on the effects of the COVID-19 pandemic; and (6) written in English. After screening 8449 titles and abstracts, 88 systematic reviews with meta-analysis met the inclusion criteria and were included in the final analysis.
