# Supplementary material for: Effectiveness of workplace interventions for health promotion
Source: Lancet Public Health. Author manuscript; Available in PMC 2025 Jun 18. (PMC12176053; doi:10.1016/S2468-2667(25)00095-7)
Supplement: 1 [file NIHMS2085668-supplement-1.pdf]

THE LANCET
Public Health

Supplementary appendix

This appendix formed part of the original submission and has been peer reviewed. We post it as supplied by the authors.

Supplement to: Virtanen M, Lallukka T, Elovainio M, Steptoe A, Kivimäki. M Effectiveness of workplace interventions for health promotion. *Lancet Public Health* 2025; **10:** e512–30.

Appendix

Effectiveness of workplace interventions for health promotion

Marianna Virtanen, Tea Lallukka, Marko Elovainio, Andrew Steptoe, Mika Kivimäki

Contents **Page**

Methods for systematic review..1

Supplementary table 1: Inclusion and exclusion criteria..2

Supplementary table 2: Search terms for PubMed...3

Supplementary figure 1: Flow chart for search strategy...4

Supplementary table 3: Details of included systematic reviews with meta-analysis..............5

Supplementary table 4: Details of excluded systematic reviews with meta-analysis............16

Overlapping analysis...23

List of previous umbrella reviews...27

References..29

Methods for systematic review

We searched from PubMed and Web of Science for all systematic reviews and meta-analyses according to pre-defined search strategies (supplementary tables 1 and 2, p. 2-3). PubMed and Web of Science databases were searched on 14 May 2024 and updated on 3 January 2025. The first screening was performed by two independent researchers (either MV, ME, TL or MK). The second screening was conducted by MV and confirmed either by ME, TL or MK. Results from the searches are presented in a flow chart (p. 4). Our search also included a cited reference search in Web of Science, a method that helps to identify additional relevant research not covered by the search terms. The inclusion and exclusion criteria are presented in supplementary table 1 (p. 2).

To assess the quality of reviews, we used the online AMSTAR-2 tool,[1] an appraisal tool for systematic reviews and meta-analyses. This tool assigns different weights to various aspects of evidence quality, prioritising critical points in the review process, meaning that the same sum score can yield different overall quality evaluations. From the initial screening of 8449 titles and abstracts, 370 systematic reviews met the initial inclusion criteria. Of these, 42 were excluded for being classified as 'critically low' quality, based on the AMSTAR-2 assessment (see supplementary table 4, p. 16). Among the remaining systematic reviews, 88 contained a meta-analysis (supplementary table 3, p. 5), while 240 were systematic reviews without meta-analysis (a full list on p. 34). Our horizontal review included the 88 reviews containing a meta-analysis.

We collected the following information from the retrieved meta-analyses: first author, publication year, number of studies included, total number of participants, proportion of studies with randomized controlled, quasi-randomized controlled, other controlled, and noncontrolled study designs, target population, content of intervention, type of intervention (universal, selective or indicative prevention), outcome(s), main findings (effect sizes, confidence intervals, heterogeneity and small-study bias estimates), and the method that the authors used to assess risk of bias in the included original studies. For one meta-analysed estimate,[2] we converted the estimate to a standardized mean difference, following a guideline with formula.[3] The meta-analyses on the same topic partially overlapped in their inclusion of primary studies. To prevent spurious estimates caused by double-counting individual studies, we chose not to perform a meta-analysis of the retrieved meta-analyses.[4]

We retrieved author-reported information on the proportion of the original intervention studies involving high/unclear risk of bias (≤40% or >40%). We found that 14 (17%) meta-analyses reported 40% or fewer original studies with a high risk of bias.[5-18] However, these meta-analyses either did not account for all items of the Cochrane Risk of Bias Tool or used a tool not comparable to the Cochrane Tool. To ensure consistency in quality assessment, we re-classified these 14 meta-analyses as having a high proportion of original studies with a high risk of bias, resulting in a downgrade of their GRADE

rating from ‘high quality’ to ‘moderate’. Consequently, the highest quality rating among all 88 evaluated meta-analyses was ‘moderate’. We considered inconsistency (i.e., high heterogeneity defined as I^2≥75%) and small-study bias (defined as publication bias detected by Egger’s test or unknown publication bias). If neither inconsistency nor small-study bias was present, the evidence was rated as remaining in ‘moderate’ GRADE; if one of these biases was identified, the rating was downgraded to ‘low’, and if both biases were present, the evidence further downgraded to ‘very low’.

Supplementary table 1. Inclusion and exclusion criteria

	**Inclusion**	**Exclusion**
**Population**	Employed populations	Non-employed populations
**Intervention**	Workplace health promotion; individual and group-level activities focusing on health improvement in the workplace setting	Health promotion outside the workplace<br>Medical treatment and rehabilitation<br>Modification of psychosocial work environment<br>Work scheduling interventions<br>Work arrangements<br>Leadership/management interventions
**Control group**	Controlled randomised and non-randomised studies (≥2 randomised controlled trials); control group without intervention or minimal intervention	Non-controlled studies
**Outcomes**	Health-related behaviours, cardiometabolic health, musculoskeletal health, mental health and stress	Diseases which are rare in employed populations (e.g., dementia); injuries, sickness absence, work disability, return to work after illness, job satisfaction
**Languages**	English	Other than English
**Other**	Systematic review and meta-analysis<br>Published in international peer-reviewed journal, not classified as predatory<br>Meta-analyses not focussing on the effects of COVID-19 pandemic	Systematic review without meta-analysis, narrative review, scoping review<br>National, non-peer-reviewed or predatory journal<br>Meta-analyses focussing on the effects of COVID-19 pandemic

Supplementary table 2. Search terms for PubMed

**Search 13 May 2024**	
	**Defining intervention**
1.	promot* OR prevent* OR educat* OR screening OR control OR management OR intervention* OR polic* OR improve* OR (employee AND incentive AND plan) OR (risk AND reduction) OR (risk AND assessment)
2.	workplace OR worksite OR organisation* OR organization* OR occupation* OR employee* OR worker* OR employer*
3.	wellness AND program*
4.	#1 OR #2 OR #3
5.	**Defining outcomes** (with separate searches in combination with line #4)
	**Work stress**: stress OR distress OR mindfulness OR psychosocial OR workload OR (work AND hours) OR (job AND strain) OR (job AND control) OR (working AND conditions) OR (physical AND strain)
	**Work environment:** nudge* OR nudging OR (work AND environment)
	**Health and general behavioural outcomes:** health OR disease OR illness OR behavioral OR behavioural OR sleep* OR insomnia OR lifestyle OR (life AND style) OR (risk AND factor) OR (risk AND reduction)
	**Weight:** obesity OR overweight OR weight OR (weight AND management) OR (weight AND reduction) OR (weight AND control)
	**Diet:** eat OR eating OR nutrition* OR diet* OR food
	**Substance use:** alcohol OR drinking OR (substance AND use) OR (substance AND abuse) OR (risky AND drinking)
	**Physical activity:** exercise OR sitting OR (physical AND activity)
	**Smoking**: smoking OR anti-smoking OR smoke-free
	**Filters:** systematic review/meta-analysis, English, humans, adults, years 2010 to present; titles/abstracts and MESH term searches
**Search 3 Jan 2025 (update)**	
1.	health OR disease OR behavioral OR behavioural OR behavior OR behaviour OR lifestyle OR (life AND style)
2.	cardiovascular OR cardiometabolic OR metabolic OR musculoskeletal OR pain
3.	exercise OR sedentary OR sitting OR (physical AND activity)
4.	weight OR overweight OR obesity OR nutrition OR eating OR healthy OR alcohol OR smoking OR physical OR mental OR distress OR depression OR stress OR burnout OR mindfulness OR sleep OR drinking OR lipid OR hypertension OR diabetes OR (blood AND pressure)
5.	workplace OR worksite OR wellness OR work OR organisation OR organization OR organisational OR organizational OR worker OR nudge
6.	intervention OR interventions OR RCT OR trial OR program OR programme OR promotion
8.	(#1 OR #2 OR #3 OR #4) AND (#5 AND #6)
	**Filters:** systematic review/meta-analysis, English, humans, adults, years 2010 to present; titles/abstracts searches

Supplementary figure 1. Flow chart for search strategy identifying systematic reviews and meta-analyses on workplace health promotion

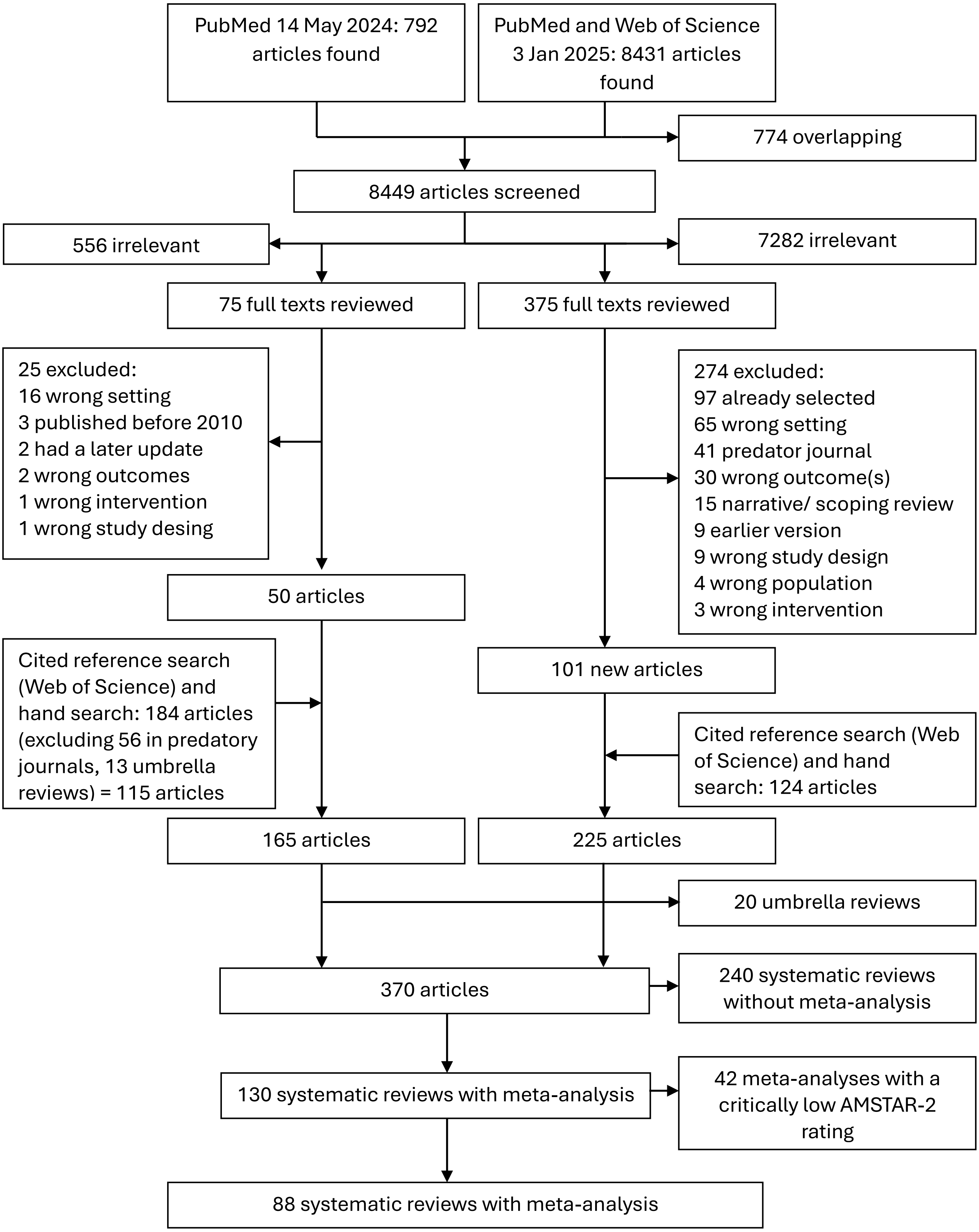

Supplementary table 3. Details of included systematic reviews with meta-analysis (n=88)

First author (year)	No. of studies in review	No. of participants	No. of RCTs/ other controlled /non-controlled	Intervention(s)	Type of prevention	Outcome (s)	Author-reported high /unclear risk of bias (in >40% of studies; yes/no)	Risk of bias method used	Quality of review (AMSTAR -2)
Halim (2023)[5]	40 (12 RCTs)	2539	12/6/22	Cognitive, exercise, assistive devices, combined	Selective (health-care workers)	Musculoskeletal disorders	No	EPHPP	Low
Barger (2018)[19]	18 (5 in meta-analysis	n.a.	3/13/2	Fatigue training; education, workshops, counselling, mindfulness, multicomponent	Selective (emergency medical workers and other shift workers)	Sleep quality, fatigue, stress, burnout	Yes	Cochrane RoB Tool; GRADE	Low
Bartlett (2019)[20]	25	2291	23/0/0	Mindfulness-based interventions in the workplace	Universal	Mindfulness, stress, mental health symptoms	Yes	Cochrane RoB tool	Low
Bellon (2019)[21]	3	1246	3/0/0	Psychological or educational interventions in the workplace	Universal /selective (symptomatic)	Depressive symptoms	Yes	Cochrane RoB tool; GRADE	High
Bezzina (2024)[22]	11	1932	11/0/0	Lifestyle interventions targeted to men	Selective (men)	Smoking, nutrition, alcohol use, physical activity, overweight and obesity	Yes	Cochrane RoB tool	Low
Burn (2019)[23]	12	826	12/0/0	Physical activity interventions of at least moderate intensity activity	Universal	Improve cardio-respiratory fitness, CRF (peak oxygen consumption, VO_{2peak})	Yes	PEDro RoB tool	High
Cahill 2014[24]	57	7419	17/0/0	Various types: group therapy, individual counselling, self-help materials, nicotine replacement therapy, social support,	Selective (smokers)	Smoking cessation	Yes	Cochrane review; GRADE	Moderate

				environmental and multicomponent					
Caputo (2022)[25]	5 (worksites)	n.a.	n.a.	Promote stair climbing (nudge measures)	Selective (office workers)	Stair climbing	Yes	Dows and Black scale, GRADE	Low
Carolan (2017)[26]	21	5260	21/0/0	Web-based psychological interventions delivered at the workplace; cognitive-behavioural therapy, mindfulness, etc.	Universal	Psychological well-being and work effectiveness	Yes	Cochrane RoB tool	Low
Chen (2018)[27]	27	8164	27/0/0	Workplace exercise; strengthening, fitness training; ergonomic interventions, education, breaks	Selective (office workers with or without neck pain)	Reducing neck pain	Yes	Cochrane Back and Neck Group RoB guideline	Low
Chu (2016)[28]	26	4586	15/11/0	Reduce sedentary time (educational/behavioural, environmental, multicomponent)	Selective (office workers)	Sedentary time	Yes	Cochrane RoB Tool	Low
Clari (2024)[6]	12	529 909	12/0/0	Interventions to improve vaccination adherence; educational, promotional, policy	Selective (healthcare workers)	Vaccine uptake (seasonal influenza)	No	Quality Assess-ment Tool for Controlled Inter-vention Studies	Low
Cochrane (2017)[29]	20	16 319	20/0/0	Early multicomponent interventions with a biopsychosocial approach, back school, or stepped care approach	Indicative (workers on sick leave due to regional musculo-skeletal pain)	Musculo-skeletal pain	Yes	Cochrane RoB Tool; GRADE	Critically low*
Coenen (2020)[7]	15	8709	15/0/0	Lifestyle interventions: advice, counselling (individual/ group activity), combined (5 studies), with environmental (3 studies) carried out in the Netherlands	11 universal, 4 selective/ indicated	Physical activity, diet, alcohol, smoking	No	Cochrane RoB tool	High
Compernolle (2019)[8]	5 (worksites)	525	3/2/0	Reduce sedentary time by self-monitoring	Selective (office workers)	Sedentary time	No	EPHPP Tool	Low
de Sevilla (2021)[10]	12	1270	12/0/0	Supervised exercise interventions in the workplace	Selective (overweight)	Body weight, fat mass, muscle mass	No	Cochrane RoB tool	Low

Eisele-Metzger (2023)[30]	24	7080	24/0/0	Multicomponent, exercise, ergonomic, education, behavioural interventions	Selective (office workers, with or without back pain)	Preventing back pain and sickness absence	Yes	Cochrane RoB Tool; GRADE	High
Fellbaum (2023)[11]	20	4484	20/0/0	Various types: lectures, lifestyle campaigns, CBT, feedback etc.	Universal /selective	Reducing alcohol consumption	No	Cochrane Risk of Bias Tool	High
Fendel (2021)[31]	25 (6 controlled studies)	925	6/3/16	Mindfulness	Selective (physicians)	Burnout, stress	Yes	Cochrane RoB Tool, Effective Public Health Practice Project Quality, GRADE	High
Fitzpatrick-Lewis (2022)[32]	5	1494	5/0/0	Three elements: health coach/ educator, focus on diet and increase in physical activity	Selective (BMI of 25.0-50.0 and pre-diabetes)	Target: 5%/7% weight loss, physical activity<br>Type 2 diabetes not measured	Yes	Cochrane RoB tool; GRADE evidence rating	Low
Freak-Poli (2020)[33]	14	4762	14/0/0	Workplace pedometer interventions	Universal/ selective (sedentary)	Physical activity, sedentary behaviour, BMI, quality of life, blood pressure	Yes	Cochrane review; GRADE evidence rating	Moderate
Frutiger (2021)[34]	29	8076	29/0/0	Strength training, tailored workplace modifications	Selective (office workers)	Reducing neck pain	Yes	Cochrane RoB tool; GRADE evidence rating	Low
Hayden (2021)[35]	21/249 with a workplace setting	1974	21/0/0	Exercise treatment; muscle strengthening, stretching, core strengthening, flexibility and mobilising exercises, aerobic	Indicative (chronic, low-back pain)	Reducing chronic low-back pain, improving function	Yes	Cochrane review; GRADE evidence rating	High

				exercises, functional restoration, McKenzie therapy, and yoga					
Heckenberg (2018)[36]	9	812	7/1/1	Mindfulness-based interventions	Universal	Physiological indices of stress (HPA axis; cortisol)	Yes	MINORS scale	Low
Hulls (2021)[37]	35	8856	27/7/1	Workplace wellness programs, e.g., education classes, workshops, counselling, exercise, mindfulness	Selective (male-dominated industries)	Improving physical and mental health, lifestyle, sleep, and stress	Yes	Cochrane RoB tool	Low
Indrayani (2024)[9]	9 (3 in meta-analysis)	1355	9/0/0	Exercise	Indicative (nursing personnel with low back pain)	Low back pain	No	Cochrane RoB Tool	Critically low*
Jones (2024)[38]	8 (5 in meta-analysis)	265	8/0/0	Exercise	Indicative (office workers with chronic neck pain)	Neck pain	Yes	Cochrane RoB Tool, GRADE	Low
Jung (2022)[39]	4 (BMI), 7 (physical activity)	4728	8/0/0	Mobile health (m-Health) interventions	Universal	Physical activity and weight loss	Yes	Cochrane RoB Tool	Low
Karo (2024)[40]	16 (12 in meta-analysis)	1372	16/0/0	Mindfulness	Selective (nurses)	Psychological distress	Yes	Cochrane RoB Tool	Low
Kunzler (2022)[41]	24	1879	24/0/0	Stress management, psychoeducation, mindfulness, cognitive methods	Indicative (nurses)	Resilience, mental health	Yes	Cochrane RoB Tool	Low
Ladino (2023)[42]	4 (2 in meta-analysis)	122	2/0/0	Internet-based psychosocial interventions	Indicative (healthcare workers)	Burnout	Yes	Cochrane RoB Tool	Low
Lam (2022)[43]	39/136 with a workplace setting	3725	Umbrella review (not reported)	Interventions to reduce sedentary behaviour (umbrella review and meta-analysis of individual studies)	Selective (office workers)	Reducing sedentary behaviour	Yes	AMSTAR-2 for systematic reviews	Low
Lee (2016)[44]	7	1463	5/2/0	Coping-oriented workplace interventions, e.g., CBT, mindfulness, stress management	Selective (nurses)	Reducing burnout symptoms	Yes	Jadad scale Cochrane RoB Tool	Low
Liu (2023)[45]	12	807	12/0/0	Mindfulness-based interventions	Indicative (nurses)	Anxiety and depression	Yes	Cochrane RoB Tool	Low

Lock (2021)[12]	83 (38 in meta-analysis)	5425	26/12/0	Physical activity interventions	Universal	Physical activity (daily steps), oxygen intake	No	EPHPP Tool	Low
Lomas (2019a)[46]	35/42	3942	35/0/0	Mindfulness-related interventions in the workplace	Universal	Reducing symptoms of stress and mental ill health	Yes	QATQS	Low
Lomas (2019b)[13]	42 (14 in meta-analysis)	1360	12/6/22	Mindfulness-based interventions	Indicative (healthcare workers)	Stress, anxiety, depression, burnout	No	QATQS Tool	Low
Louw (2017)[47]	8 (2 in meta-analysis)	2075 (385 in meta-analysis)	8/0/0	Exercise	Indicative (office workers with neck pain)	Neck pain	Yes	PEDro scale	Low
Lowry (2017)[48]	13	5747	11/0/0	Workplace exercise training, e.g., resistance training, stretching, stabilisation, whole-body exercise, ergonomic interventions (e.g. workstation change) etc.	Indicative (Shoulder disorders)	Reduce shoulder pain	Yes	Cochrane RoB tool; GRADE	Critically low*
Lu (2023)[49]	12	2298	0/12/0	Psychological training programs	Indicative (police personnel)	Stress, resilience, depression, anxiety	Yes	Downs and Black checklist	Moderate
Luger (2019)[50]	6	373	6/0/0	Work-break schedules	Selective (office workers)	Preventing onset of musculo-skeletal disorders or symptoms, or fatigue	Yes	Cochrane review; GRADE	High
Martin-Gill (2018)[51]	13	293	2/11/0; lab. based studies	Napping during shift work	Selective (shift workers, mostly healthcare workers)	Reaction time, sleep quality, sleepiness	Yes	Cochrane RoB Tool; GRADE	Low
Merom (2021)[52]	18	3309	12/2/4	Physical activity.<br>7 studies with multicomponent interventions (screening, nutrition); coaching, group sessions; mainly aerobic exercise	Selective (older, with CVD risk factors/ obesity)	Increase in physical activity	Yes	Cochrane RoB and Robins-I tool GRADE	Low

Michaelsen (2023)[53]	92	9375	92/0/0	Mindfulness	Universal/ selective	Mental health, stress, resilience, physical health	Yes	Cochrane RoB Tool	High
Montano (2014)[54]	36	17 942	36/0/0	Worksite interventions, e.g., education, counselling, ergonomic improvements, exercise, environmental changes (e.g. food choices) etc.	Universal	BMI, fruit and vegetable consumption, musculo-skeletal symptoms, stress	Yes	Cochrane RoB Tool; GRADE	Low
Mulchandani (2019)[55]	33	36 188	27/6/0	Physical activity interventions: information, workshops, behavioural change, coaching, and environmental changes; 28/33 studies used multicomponent approaches	Universal / selective (older, overweight, CVD risk factors)	Improving cardio-metabolic health	Yes	Cochrane RoB tool	Low
Nigatu (2019)[56]	15	4258	14/1/0	Cognitive-behavioural therapy (CBT) in the workplace setting (10 studies), other interventions, e.g., web-based self-help, exercise, educational, workshops, and multicomponent	Indicative (workers with depressive symptoms)	Reducing depressive symptoms, preventing depressive disorder	Yes	Cochrane RoB Tool	Low
Panagioti (2017)[57]	19	1550	17/2/0	Organization-directed and physician-directed workplace interventions	Selective (physicians)	Reducing burnout	Yes	EPOC	Low
Panchbhaya (2021)[58]	39	7324	16/14/4	Workplace dietary interventions within a healthcare setting; environmental, educational and behavioural, or combinations of these	Selective (healthcare workers)	Increasing fruit & vegetable intake, decreasing energy and fat intake; Improvement in BMI, weight, blood pressure, cholesterol	Yes	Cochrane and ROBINS-I RoB tools	Critically low*

Pares-Salomon (2024)[14]	19	3529	19/0/0	Workplace interventions with digital elements, e..g., information delivery and mediated organisational support and social influences	Selective (office workers)	Reducing sedentary behaviours	Yes	QUALSYST Tool	Critically low*
Park (2019)[15]	7	2854	7/0/0	Dietary interventions	Selective (overweight/ obesity)	BMI, cholesterol, blood pressure	No	Cochrane RoB Tool	Low
Parry (2019)[59]	10	955	8/2/0	Increasing standing or walking, e.g. sit-stand or treadmill workstations, activity trackers; multicomponent (5 studies)	Indicative (sedentary & musculo-skeletal symptoms)	Reduction of musculo-skeletal symptoms	Yes	Cochrane review; GRADE evidence rating	High
Paudel (2022)[60]	26	2472	24/0/2	Workplace stress management interventions; meditation, CBT, other (e.g., coaching, workshops)	Selective (teachers)	Reducing stress	Yes	Robins-I Tool; GRADE evidence rating	Low
Peachey (2020)[61]	18/35 with a workplace setting	1627	18/0/0	Active workstations, apps, prompts, text messages, educational sessions, programs	Selective (office workers)	Reducing sitting time	Yes	Cochrane RoB and Robins-I tool; GRADE	Low
Penalvo (2021)[62]	121	4081	82/39/0	Multicomponent workplace wellness programs; educational component, environmental changes (cafeteria, stair use etc.), financial incentives, physical activity	Universal / selective	Improving diet, over-weight, and cardio-metabolic health	Yes	Bias score 0-5	Low
Petrie (2019)[63]	8	1023	7/1/0	Physician-directed (group or individual, e.g., stress management, psychoeducation, CBT, mindfulness); organizational-level interventions (no studies found)	Selective (physicians)	Reducing mental health symptoms and suicidal ideation	Yes	Cochrane RoB Tool	Low
Phillips (2019)[64]	50 (34 in meta-analysis)	15 258	34/0/0	Occupational e-mental health interventions	Universal /selective	Insomnia, burnout, depression, anxiety, alcohol use	Yes	Cochrane RoB Tool	Low

Picon (2021)[65]	27	3292	27/0/0	Workplace-based physical exercises, ergonomics and combinations	Indicative (workers with shoulder disorders)	Reduce shoulder pain	Yes	PEDro Scale, GRADE evidence rating	Low
Power (2014)[16]	13	3751	13/0/0	Diet and physical activity interventions; single or multicomponent	Selective (healthcare workers, overweight /obese)	Weight /body mass index loss	No	Public Health Practice Project Quality Assess-ment Tool for Quan-titative Studies	High
Prieske (2019)[66]	17	3423	17/0/0	Workplace exercise training; resistance, endurance, team sports, multicomponent training	Universal	Improvement in cardio-respiratory fitness (CRF), muscular endurance, and muscle power	Yes	PEDro RoB scale	Critically low*
Prudenzi (2021)[67]	22 (10 in meta-analysis)	920	10/0/0	Group-based acceptance and commitment therapy	Selective (healthcare workers)	Psychological distress	Yes	POMRF Tool	Low
Reed (2017)[68]	24 (20 in meta-analysis)	4707	19/1/0	Physical activity	Universal (women)	Physical activity and cardio-metabolic health	Yes	Cochrane RoB Tool, GRADE	Low
Richardson (2019)[69]	99 (4 in workplaces)	763 (in workplaces)	4/0/0	Workplace obesity prevention	Universal	Energy expenditure	Yes	GRADE	Low
Robroek (2020)[17]	13	5183	10/3/0	Workplace interventions grouped into: agentic (e.g., counselling, health education) and structural (e.g., changing the food environment, removing vending machines) carried out in the Netherlands	Universal / selective	BMI	No	Cochrane RoB tool-and Verweij et al. tool	Moderate

Rongen (2013)[70]	18	5755	18/0/0	Workplace health promotion programs (e.g., physical activity, smoking cessation, diet, weight loss, stress management; single or multicomponent)	Universal / selective	Self-perceived health	Yes	Cochrane RoB Tool	Critically low*
Ruiz-Fernandez (2020)[71]	9	438	4/5/0	Mindfulness	Selective (healthcare workers)	Stress	Yes	Cochrane RoB Tool	Low
Ruotsalainen (2015)[72]	58	7188	54/4/0	Cognitive-behavioural training, relaxations, or both; organizational interventions	Selective (nurses)	Preventing stress	Yes	Cochrane review; GRADE	High
Shrestha (2018a)[73]	34	3397	17/17/0	Workplace interventions for reducing sitting; e.g., physical workplace changes, policy changes, information and counselling, multiple methods	Selective (office workers)	Reducing sitting at work	Yes	Cochrane RoB tool; GRADE	High
Shrestha (2018b)[74]	17	14 272	10/0/7	Worksite dietary interventions; individual and environmental-focused	Universal /selective /indicative	Prevention and management of type 2 diabetes; reduction in HbA1c and fasting glucose	Yes	Quality score	Low
Slanger (2016)[75]	17 (2 in meta-analysis)	556	2/0/0	Bright light, napping, other (e.g., exercise)	Selective (shift workers)	Sleepiness, sleep disturbances	Yes	Cochrane RoB Tool, GRADE (very low)	Moderate
Slemp (2019)[76]	119	6044	54/19/44	Workplace contemplative interventions, e.g., mindfulness, meditation, acceptance and commitment therapy	Universal / selective?	Reducing psychological distress	Yes	Downs and Black Tool	Low
Stratton (2022)[77]	75 (29 anxiety, 46 depression 57 stress)	4961 (anxiety), 6989 (depression, 10160 stress)	75/0/0	Workplace eHealth interventions	Universal	Mental health (depression, anxiety, stress)	Yes	Cochrane RoB Tool	Low

Tamminga (2023)[78]	117	11 119	117/0/0	Interventions based on CBT, exercise, relaxation, yoga or a combination of those	Selective (healthcare workers)	Reducing occupational stress	Yes	Cochrane review; GRADE	High
Tan (2022)[79]	7	18818	7/0/0	Psychosocial interventions	Selective (first responder (e.g., emergency services, military)	Post-traumatic stress disorder (PTSD), common mental disorder	Yes	Downs and Black Tool	Low
Tang 2020[80]	9 (3 in meta-analysis)	326	0/3/0	Workplace breastfeeding promotion	Selective (women with babies)	Breastfeeding	Yes	MMAT	Low
Teichert 2023[81]	5	1722	5/0/0	Exercise interventions	Selective (mainly office workers)	Preventing onset of neck pain	Yes	Cochrane RoB tool; GRADE	Moderate
van Vilsteren (2015)[2]	14	1897	14/0/0	Workplace interventions	Indicative (sickness absentees)	Reducing musculo-skeletal pain	Yes	Cochrane review; GRADE	Moderate
Verbeek (2012)[82]	7	1929	7/0/0	Manual material handling advice at work	Universal / selective (workers with back pain)	Preventing and reducing back pain	Yes	Cochrane review; GRADE	High
Verhagen 2013[83]	44	6580	44/0/0	Exercises, ergonomics, behavioural and other interventions	Indicated (workers with work-related arm, neck, or shoulder pain)	Work-related arm, neck, and shoulder disorders (pain, disability, sick leave)	Yes	Cochrane review; GRADE	Moderate
Verweij (2011)[84]	43	48 627	43/0/0	Workplace physical activity and diet interventions, e.g., exercise, education, workshops	Universal (normal weight)	Preventing overweight/ obesity	Yes	Cochrane Handbook quidelines; GRADE	Critically low*
Virga (2022)[85]	21	2922	all RCT or other controlled	Interventions promoting strengths	Universal /selective	Personal resources	Yes	Cochrane RoB Tool	Low
Vonderlin (2020)[86]	56	5161	56/0/0	Mindfulness-based interventions	Universal/ selective	Stress, burnout resilience, somatic	Yes	Cochrane RoB Tool	Low

						symptoms, anxiety, depression, etc.			
Wang (2024)[18]	24	3169	21/3/0	Workplace interventions, e.g., environmental support, motivational strategies, or multicomponent interventions	Selective (office workers)	Reducing sitting at work; secondary outcomes physical and mental health	No	Cochrane RoB tool; GRADE	Low
West (2016)[87]	52 (15 RCTs)	3630	15/37/0	Workplace interventions; individual (e.g., small groups, stress management) and structural / organizational focused interventions, e.g., practice-delivery changes.	Selective (physicians, with or without burnout symptoms)	Preventing and reducing burnout	Yes	Cochrane RoB Tools	Low
Yuvaraj (2019)[88]	7	1291	7/0/0	Workplace-initiated counselling, web-based interventions	Universal (alcohol users)	Alcohol consumption	Yes	Cochrane RoB Tool	Low
Zhang (2021)[89]	15	688	15/0/0	Physical relaxation: yoga, stretching, massage therapy, and progressive muscle relaxation	Selective (healthcare workers)	Reducing occupational stress	Yes	Cochrane RoB Tool	Low
Zhou (2023a)[90]	23	1428	23/0/0	Active workstations + other promotion, e.g., reminders	Selective (office workers)	Reducing sitting at work	Yes	Cochrane RoB tool; GRADE	Low
Zhou (2023b)[91]	11	1894	11/0/0	Multicomponent interventions to mitigate occupational sedentary behaviour; individual strategies (e.g., counselling, prompts), environmental changes (e.g., active workstations) and organizational strategies (e.g., workshops, site visits, management support)	Selective (office workers)	Reducing sitting at work	Yes	Cochrane RoB tool; GRADE	Critically low*

*We included reviews rated as critically low quality only if their primary limitation was the absence of a publication bias assessment.

Abbreviations: BMI, body mass index; CBT, cognitive behavioural therapy; CVD, cardiovascular disease; EPHPP, Effective Public Health Practice Project; EPOC, Effective Practice and Organisation of Care; GRADE, Grading of Recommendations, Assessment, Development, and Evaluations; HPA axis, hypothalamus-pituitary-adrenal axis; MMAT, Mixed Methods Appraisal Tool; PEDro, Physiotherapy Evidence Database Tool; POMRF, Psychotherapy Quality Rating Scale; QATQS, Quality Assessment Tool for Quantitative Studies; QUALSYST, Quality Assessment Tool; RCT, randomized controlled trial; RoB, risk-of-bias; ROBINS, Risk Of Bias In Non-randomized Studies Tool.

Supplementary table 4. Details of excluded systematic reviews with meta-analysis (n=42) due to a critically low AMSTAR-2 rating

First author (year)	No. of studies in review	No. of participants	No. of RCTs/ other controlled /non-controlled	Intervention(s)	Type	Outcome (s)	Results	Author-reported high /unclear risk of bias (in >40% of studies; yes/no)	Risk of bias method used
Agarwal (2018)[92]	8 (meta) + 4 (not meta)	206	1/10/1	Sit-stand workstations	Selective (office work)	Low-back discomfort	Decreased low-back pain (SMD=0.23, 95% CI= 0.02-0.44)	Not reported	Not assessed
Andrews (2019)[93]	9	462	9/0/0	Exercise	Selective (fire fighters)	Health and fitness	Improved health and fitness (d=0.94, 0.67-1.21)	Not reported	Not assessed
Antipas (2024)[94]	10	1397	10/0/0	Organisational and employee-focused stress management	Selective (dementia care employees)	Stress	STD mean difference, total effect: -0.27 (-0.64-0.11)	No	Cochrane RoB Tool, Downs and Black Tool
Ayaz (2024)[95]	19	19394	19/0/0	Motivational, group counselling, telephone coaching, web-based training, mindfulness, and financial interventions	Selective (smokers)	Smoking	Improved quitting /reducing smoking (Hedge's g=1.17, 1.03-1.33)<br>Significant heterogeneity	Not reported	Not assessed
Beames (2023)[96]	46	5685	23/23/0	Stress management	Selective (teachers)	Mental health, stress, sleep, wellbeing	Separate analyses for RCTs and non-RCTs. Mainly positive or null findings for various outcomes.	Yes	Cochrane RoB Tool II, Robins I
Burton (2017)[97]	9	284	2/0/7	Mindfulness-based interventions in the workplace	Selective (healthcare workers)	Stress	Meta-analysis suggests a positive impact for the intervention to reduce stress	Yes	QATSDD
Busireddy (2017)[98]	19	2537	6/7/6	Work hour reductions	Selective (resident physicians)	Burnout	Emotional exhaustion: OR=0.59 (0.44-0.79)<br>Depersonalization: 0.86 (0.64-1.14)<br>Personal accomplishment: 1.11 (0.74-1.65)<br>Overall burnout: 0.60 (0.37-0.98)	No	Jadad & Newcastle -Ottawa scales

							No heterogeneity		
De Simone (2021)[99]	19	1750	19/0/0	Stress management (mindfulness, education, exercise)	Selective (physicians)	Burnout	Physician-focused interventions, reduced burnout: SMD= 0.18 (0.04−0.32); no heterogeneity	Yes	Cochrane RoB Tool
Dharma-wardene (2016)[100]	12	488	6/6/0	Meditative interventions	Selective (healthcare workers)	Physical and mental health	Improvement in emotional exhaustion: SMD= 0.37 (04-0.70); personal accomplishment: SMD=1.18 (0.10-2.25)	Yes	Downs and Black Tool
Dreison (2018)[101]	13	863	13/0/0	Job training, education, person-focused (e.g., stress management)	Selective (healthcare)	Burnout	Overall mean=0.20 (0.02-0.38)<br>Emotional exhaustion mean=0.21 (0.04-0.39)<br>Depersonalisation mean=0.36 (0.13-0.59)<br>Accomplishment mean=0.03 (-0.26-0.31)	Yes	Four quality aspects assessed
Guilllaumie (2017)[102]	17/32 (controlled studies)	885 in RCTs	17/0/15	Mindfulness in the workplace setting; e.g., mindfulness-stress reduction programme (MBSR)	Selective (nurses)	Occupational stress	Meta-analysis of RCT studies showed a significant reduction in anxiety (6 studies, high heterogeneity) and depressive symptoms (4 studies, no heterogeneity); no effect on blood pressure or pulse rate (3 studies, no heterogeneity)	Yes	Cochrane RoB tool
Haslam (2024)[103]	38	2010	33/5/0	Mindfulness, education, coaching, schedule change	Selective (physicians)	Burnout	Mean difference for emotional exhaustion: 1.11 (0.09-2.14); depersonalisation 0.32 (0.04-0.61); personal accomplishment 1.11 (0.21-2.43); high heterogeneity	Yes	Cochrane RoB Tool
Hutchinson (2012)[104]	31	12 411	all with a control group	Workplace interventions for nutrition and physical activity	Universal / selective?	Lifestyle and cardio-metabolic risk factors	Meta-analysis showed a negative effect of motivational enhancement on physical activity (5 studies) but a positive effect of exercise on fitness (4	Not reported	Not assessed

							studies); increase in cholesterol (3 studies); decrease in weight (4 studies); decreased systolic and diastolic blood pressure (3 studies) Fruit & vegetable use (8/10),		
Kang (2020)[105]	13 (6 in meta-analysis)	471	7/6/0	Stress management (aromatherapy)	Selective (shift-working nurses)	Sleep quality	SMD (improved sleep)=0.97 (0.64-1.29); heterogeneity	No	Cochrane RoB Tool
Klingbeil (2018)[106]	29	1493	18/11	Mindfulness	Selective (teachers)	Psychological distress, physical health	Hedges' g=0.55 (0.37-0.73) for psychological distress; g=0.62 (0.20-1.03) for physical health; high heterogeneity	Not reported	Not assessed
Kröll (2017)[107]	43	22 822	All were controlled interventions (no. of RCTs not reported)	Workplace stress interventions; individual-focused (stress management training), work-focused (flexible work arrangements, work accommodation)	Universal	Improving psychological health and reducing absenteeism	Meta-analysis showed that work adjustments and telecommuting had favourable impact on psychological health (7 and 6 studies, high heterogeneity); no impact of work adjustments on absenteeism (3 studies); of individual-focused interventions, cognitive-behavioural skills training (5 studies, low heterogeneity); relaxation techniques had positive impact (7 studies, high heterogeneity); multimodal stress management training (9 studies, low heterogeneity) had positive impact on psychological health	Yes	Quality assess-ment tool
La Torre (2020)[108]	27 (4 RCTs)	1280	7/0/20	Pharmacological and behavioural interventions	Indicative (healthcare workers, smokers)	Smoking cessation	RR=1.24 (1.10-1.41) for smoking cessation in RCT studies; high heterogeneity	No	Cochrane RoB Tool, Newcastle -Ottawa Tool

Leeks (2010)[109]	14	n.a.	n.a.	Worksite-based incentive and competitions combined with additional interventions	Selective (smokers)	Smoking	Abstinence rate was 4.4 percentage points (2.7-9.4) higher in the intervention groups	No	Pre-defined quality criteria
Li (2024)[110]	44	n.a.	n.a.	Interventions targeting well-being	Indicative (teachers)	Well-being	Hedges' g: well-being 0.44 (0.34-0.55); Reduced symptoms: 0.31 (−0.03-0.65); several interactions found	Not reported	Thomas et al. model
Liu (2020)[111]	36, 20, 145, 82 (workplace setting)	n.a.	n.a.	Interventions to improve resilience	Universal	Resilience	Hedges' g: action=0.69 (0.52-0.87); emotion=0.59 (0.35-0.82); symptoms=0.36 (0.28-0.44); well-being=0.45 (0.35-0.54)	Not reported	Not assessed
Maricutoiu (2016)[112]	47	Not reported	34/13/0	Interventions to prevent burnout, group interventions (42 studies), individual interventions (5 studies)	Universal / selective (healthcare professionals)	Preventing employee burnout	Meta-analysis showed a small effect on general burnout (13 studies) and exhaustion scales (34 studies), with low heterogeneity between studies.	Not reported	Not assessed
Moreira-Silva (2016)[113]	12	1913	5/7/0	Physical activity interventions	Indicative (workers with pain)	Musculoskeletal pain	Hedges' *g*, overall pain: 0.40 (0.02-0.78); neck and shoulder pain: 0.37 (0.12-0.63); low back pain 0.21 (-0.1-0.58) upper extremity pain 0.07 (-0.22-0.36)	No	Cochrane RoB Tool
Neuhaus (2014)[114]	38	984	6/19/13	Workstation interventions, e.g., st-stand, walking, or cycling	Selective (office workers)	Reducing sedentary behaviours and improving health (weight, waist circumference blood biomarkers, musculo-	Meta-analysis showed a significant reduction in sedentary behaviours (mean 77 min/day, with high heterogeneity). Other outcomes: musculoskeletal (40/127 comparisons), weight (2/9), body weight /fat (7/30), blood profile	Yes	Quality score

						skeletal symptoms, fatigue and other physiological measures)	(9/34), fatigue (5/10), absenteeism (0/5)		
Ochentel (2018)[115]	4	312	4/0/0	Exercise	Indicative (persons with burnout)	Burnout	0.16 (-0.09-0.41)	Yes	PEDro Tool
Oye-Somefun (2021)[116]	13 (6 work-places, 7 lab. based)	351	2/4/0 of workplace studies	Treadmill desks	Selective (sedentary jobs)	Sitting time	Effect found for sitting time 1.73 min/h (0.17-3.30)	Not reported	Cochrane RoB Tool
Patterson (2014)[117]	12	906	9/3/0	Stress management	Selective (police officers)	Physical and mental health	Hedges' g=0.20 (-0.19-0.57) for physical health (2 studies)<br>g=0.04 (-0.16-0.23) for mental health (12 studies)	No	Maryland scale of Scientific Methods
Podrekar (2021)[118]	22	622	1/21/0	Cycle and treadmill desks	Selective (sedentary workers)	Energy expenditure, cardio-metabolic health	SMD, energy expenditure: 3.84 (2.44-5.23)<br>Heart rate: 1.68 (1.13-2.23)<br>No effect on blood pressure<br>Lowered glucose and insulin levels (p<0.001),<br>No effect on lipids, dopamine or cortisol	Yes	PEDro Tool
Rama-chandran (2023)[119]	14	1077	14/0/0	Mindfulness interventions	Selective (nurses)	Psychological well-being, burnout, PTSD	Significant reduction of psychological distress; no effect on other outcomes	Yes	Cochrane RoB Tool
Reeve (2018)[120]	7	386	5/0/2	Acceptance and commitment therapy	Selective (healthcare workers)	Reducing burnout symptoms	Meta-analysis on 4/7 studies suggests no impact. Of all studies, 4/7 reported positive findings.	No	ICROMS Tool
Regehr (2014)[121]	12	1034	6/4/2	Cognitive, behavioral, and mindfulness interventions	Selective (physician)	Anxiety and burnout symptoms	Reduced anxiety: SMD= 1.07 (0.74-1.39)<br>burnout: SMD=0.38 (0.26-0.49)	Not reported	Cochrane handbook
Spinelli (2019)[122]	39	2505	39/0/0	Mindfulness	Selective (healthcare workers)	Anxiety, burnout, depression, stress,	Significant impact on most of the outcomes (Hedges' g)	Yes	Cochrane RoB Tool

						physical health,			
Stephenson (2017)[123]	8 in workplaces	762	8/0/0	Digital technologies	Universal	Sitting time	Mean reduction 39.88 (20.18-59.58) minutes/day	Yes	Cochrane RoB Tool
Sun (2021)[124]	31	7116	31/0/0	Exercise, education, psychological, multimodal	Indicative (nurses with back pain)	Low-back pain	SMD=0.97 (0.56-1.38) for reduction of back pain	Yes	Cochrane RoB Tool
Tan (2014)[125]	9	2178	9/0/0	Workplace intervention; cognitive-behavioural techniques (CBT)	Universal	Preventing depression	Meta-analysis based on 9 studies suggests a favourable impact (no heterogeneity); subgroup analysis (CBT, 5 studies), favourable effect, no heterogeneity	No	Downs and Black checklist
Uslu (2022)[126]	79	Not reported	79/0/0?	Wellness interventions, e.g. psychoeducation, stress management, training, mindfulness, resilience	Universal / selective	Outcomes divided into: attitudinal (e.g. job satisfaction, affective well-being, stress, burnout, commitment) and motivational (resilience, self-efficacy)	Effect on both attitudinal and motivational outcomes. No difference between single or multiple-method interventions. Individual-based training more effective than group-based training. Web-based training more effective than face-to face.	Not reported	Not assessed
Van Heijster (2021)[127]	6	1906	6/0/0?	Workplace health promotion programs to improve lifestyle and prevent obesity; mainly counselling, one study with an environmental component. Carried out in the Netherlands	Selective (workers with low socio-economic position, workers with health risks)	Self-perceived health	No overall effect on self-perceived health. No difference in gender, age and marital status groups.	Not reported	Cochrane RoB Tool
Vanhove (2016)[128]	37 (22 in meta-analysis)	4148	12/10/15	Resilience interventions	Universal	Resilience	Hedges' d RCTs=0.18 (0.04-0.31)<br>non-RCTs=0.12 (0.00-0.24)	Not reported	Not assessed

Wang (2024)[129]	21	not reported	21/0/0	Loving-kindness and compassion meditation at the workplace	Universal /selective	Burnout, stress, mental health, psychological resources	Decreased burnout (10 studies): g = 0.40<br>Stress (10 studies): g=0.54<br>Mental health (13 studies) g=0.31<br>Psychological resources (6 studies): g=0.41	Yes	Cochrane RoB Tool
Wasson (2020)[130]	27	327 in controlled studies	7/4/16	Workplace mindfulness-based interventions; 25 were group-based interventions	Selective (healthcare workers)	Improving self-compassion	Meta-analysis of 6 RCT studies suggests improvement in self-compassion, with moderate heterogeneity	Yes	Jadad score
Witt (2017)[131]	13 (5 in meta-analysis)	455878	2/3/0	Workplace suicide prevention programs	Selective (emergency and protective services employees)	Suicide	Reduced suicide risk: IRR=0.45 (0.31–0.65)	Yes	GRADE
Yu (2024)[132]	18	1431	18/0/0	Digital and face-to-face interventions	Selective (nurses)	Resilience	Improved resiliece: SMD=0.71 (0.13-1.29)	Yes	JBI Tool
Zarate (2019)[133]	18	1001	12/6/0	Mindfulness interventions	Selective (teachers)	Stress, anxiety. burnout, depression	Stress (10 studies): SMD=0.53 (0.30-0.76)<br>Anxiety (8 studies): 0.52 (0.25-0.78)<br>Burnout (8 studies): 0.33 (0.15-0.52)<br>Depression (7 studies): 0.67 (0.42-0.92)	Yes	CEC scale

Abbreviations: CBT, cognitive-behavioural therapy; CEC, Council for Exceptional Children; ICROMS Tool, Intergated Quality Criteria for Review of Mutiple Study Designs; IRR, incidence rate ratio; PEDro, Physiotherapy Evidence Database Tool; PTSD, post-traumatic stress disorder; QATSDD, Quality Assessment with Diverse Studies Tool; RCT, randomized controlled trial; RoB, risk of bias; RR, risk ration; SMD, standardized mean difference

Overlapping analysis of original studies in mindfulness reviews

OUTCOME: STRESS	Michaelsen 2023	Slemp 2019	Lomas 2019b (Health care	Karo 2024 (Nurses)
Aikens 2014	Overlapping	Overlapping		
Allexandre 2016 1	Overlapping	Overlapping		
Allexandre 2016 2	Overlapping	Overlapping		
Allexandre 2016 3	Overlapping	Overlapping		
Arredondo 2017	Not overlapping			
Baby 2019	Not overlapping			
Bartlett 2017	Overlapping	Overlapping		
Bhandari 2010	Not overlapping			
Bhandari 2017	Not overlapping			
Brinkborg 2011	Overlapping	Overlapping		
Calder Calisi 2017	Not overlapping			
Chin 2019	Not overlapping			
Christopher 2018	Not overlapping			
Coelhoso 2019	Not overlapping			
Cook 2017	Not overlapping			
Dahl 2019	Not overlapping			
Dahl & Dlugosch 2020	Not overlapping			
Elder 2014	Overlapping	Overlapping		
Franco 2010	Not overlapping			
Gregoire & Lachance 2015	Overlapping	Overlapping		
Gregoire 2015	Not overlapping			
Hartfiel 2012	Not overlapping			
Huang 2015	Overlapping	Overlapping		
Ireland 2017	Not overlapping			
Jennings 2017	Overlapping	Overlapping		
Klatt 2009	Overlapping	Overlapping		
Klatt 2017	Overlapping	Overlapping		
Lacerda 2018	Not overlapping			
Lebares 2019	Not overlapping			
Lemaire 2011	Not overlapping			
Lilly 2019	Not overlapping			
Lin 2015	Not overlapping			
Lin 2019	Overlapping			Overlapping
Maddux 2018	Not overlapping			
Manotas 2014	Overlapping	Overlapping	Overlapping	
Masih 2020	Not overlapping			
McConachie 2014	Overlapping	Overlapping		
Mino 2006	Not overlapping			
Mistretta 2018	Not overlapping			
Pang & Ruch 2019	Not overlapping			
Pipe 2009	Not overlapping			
Rao 2017	Not overlapping			
Riley 2017	Not overlapping			
Roeser 2013	Overlapping	Overlapping		
Schroeder 2018	Not overlapping			
Shonin 2014	Not overlapping			
Singh 2016	Not overlapping			
Singh 2020	Not overlapping			
Slutsky 2019	Not overlapping			
Smith 2020	Not overlapping			
Sood 2011	Not overlapping			
Sood 2014	Not overlapping			
Sutarto 2012	Not overlapping			
Taylor 2016	Overlapping	Overlapping		
Versluis 2018	Not overlapping			
Wolever 2012 1	Overlapping	Overlapping		
Wolever 2012 2	Overlapping	Overlapping		
Yang 2018	Not overlapping			
Zolnierczyk-Zreda 2016	Not overlapping			
Number of included studies	59	17/ 32	1/7	1/5

Overlapping with Michaelsen
Not overlapping

Michaelsen 2023: 19/59 (32.2%) primary studies included in other reviews

Slemp 2019: 17/32 (30.8%) primary studies included in this review were also included in Michaelsen 2023

Karo 2024 and Lomas 2019b: 1 of 5 or 7 (14.3–33.3%) primary studies were also included in Michaelsen 2023

OUTCOME: BURNOUT					
OUTCOME: BURNOUT	Michaelsen 2023	Slemp 2019	Lomas 2019 a	Lomas 2019b (Health care)	Fendel 2021 (Physicians)
Alexander 2015					
Allexandre 2016					
Amutio 2015					
Arredondo 2017					
Brinkborg 2011					
Christopher 2018					
Dahl 2019					
Dahl & Dlugosch 2020					
Elder 2014					
Flook 2013					
Gregoire & Lachance 2015					
Hulsheger 2013					
Ireland 2017					
Lebares 2019					
Mackenzie 2006					
Michel 2014					
Möltner 2018					
Pandya 2019					
Rexroth 2017					
Riley 2017					
Roeser 2013					
Schroeder 2018					
Singh 2020					
Watanabe 2019					
Number of included studies	24	6/24	6/14	1/4	2/5

Overlapping with Michaelsen
Not overlapping

Michaelsen 2023: 10/24 (41.2%) primary studies included in other reviews

Stemp 2019: 6/24 (25.0%) primary studies included in this review were also included in Michaelsen 2023

Lomas 2019, Fendal 2021: 6 of 14, 1 of 4and 2 of 5 (20–42.9%) primary studies included in these reviews were also included in Michaelsen 2023

OUTCOME: DEPRESSION	Michaelsen 2023	Slemp 2019	Karo 2024 (Nurses)	Lomas 2019b (Health care)
Baccarani 2013				
Bostock 2019				
Calder Calisi 2017				
Christopher 2018				
Elder 2014				
Franco 2010				
Lacerda 2018				
Maddux 2018				
Manotas 2014				
Mino 2006				
Mistretta 2018				
Molek-Winiarska 2018				
Pipe 2009				
Riley 2017				
Shonin 2014				
Sutarto 2012				
Tahamsebi 2018				
Watanabe 2019				
Wolever 2012				
Wolever 2012				
Wolever 2012				
Yang 2018				
Number of primary studies	22	4/13	1/5	1/3

Overlapping with Michaelsen

Not overlapping

Michaelsen 2023: 7/22 (31.8%) primary studies included in other reviews

Slemp 2019: 4/13 (30.8%) primary studies included in this review were also included in Michaelsen 2023

Karo 2024 and Lomas 2019b: 1 of 3 or 5 (20.0–33.3%) primary studies were also included in Michaelsen 2023

**OUTCOME: MINDFULNESS**	**Michaelsen 2023**	**Vonderlin 2020 (post intervention)**	**Vonderlin 2020 (post intervention)**	**Lomas 2019b (Health care)**
Aikens 2014	blue	blue		blue
Alexander 2015	pink	blue		
Allexandre 2016 1	blue	blue	blue	
Allexandre 2016 2	blue	blue	blue	
Allexandre 2016 3	pink			
Amutio 2015	blue	blue		
Arredondo 2017	pink			
Bartlett 2017	blue	blue		
Christopher 2018	blue	blue	blue	
Crain 2017	blue	blue	blue	
Dahl 2019, 2020	pink			
Feicht 2013	pink			
Flook 2013	pink			
Gregoire & Lanchane 2015	pink			
Gregoire 2015	blue	blue		
Hulsheger 2013	pink			
Hulsheger 2015	pink			
Jennings 2017	blue	blue		
Klatt 2009	blue	blue		
Krick & Felfe 2020	pink			
Lacerda 2018	blue	blue		
Lebares 2019	pink			
Lilly 2019	pink			
Ludwigs 2019	pink			
Maddux 2018	pink			
Manotas 2014	blue	blue		blue
Masih 2020	pink			
Michel 2014	blue	blue	blue	
Möltner 2018	pink			
Nubold 2019	pink			
Querstret 2017	blue	blue		
Rexroth 2017	blue	blue	blue	
Roeser 2013	blue	blue	blue	
Schroeder 2018	blue	blue	blue	
Sood 2014	pink			
Valley & Stallones 2017	blue	blue		
van Berkel 2014	pink			
Van Dongen 2016	pink			
Versluis 2018	pink			
Wolever 2012	blue	blue		
Wolever 2012	blue	blue		
Wolever 2012	blue	blue		
Number of included studies	39	22/ 32	8/11	2/5

Overlapping with Michaelsen (blue)
Not overlapping (pink)

Michaelsen 2023: 19/39 (48.7%) primary studies included in other reviews

Vonderlin 2020: 22/32 (68.8%) and 8/11 (72.7%) primary studies included in these reviews were also included in Michaelsen

Lomas 2019b: 2 of 5 (40.0%) primary studies included in this review were also included in Michaelsen 2023

List of previous umbrella reviews

We retrieved the relevant systematic reviews and meta-analyses from the following previously published umbrella reviews and overviews of systematic reviews:

Adnan NBB, Dafny HA, Baldwin C, et al. What are the solutions for well-being and burn-out for healthcare professionals? An umbrella realist review of learnings of individual-focused interventions for critical care. *BMJ Open* 2022;12: e060973.[134]

Antony J, Brar R, Khan PA, et al. Interventions for the prevention and management of occupational stress injury in first responders: a rapid overview of reviews. *Syst Rev* 2020; 9: 121.[135]

Cleland CL, Jones S, Moeinaddini M, et al. Complex interventions to reduce car use and change travel behaviour: An umbrella review. *J Transport Health* 2023; 31: 101652.[136]

Di Mario S, Cocchiara RA, La Torre G. The use of yoga and mindfulness-based interventions to reduce stress and burnout in healthcare workers: An umbrella review. *Altern Ther Health Med* 2023; 29: 29-35.[137]

Fadel M, Roquelaure Y, Descatha A. Interventions on well-being, occupational health, and aging of healthcare workers: A scoping review of systematic reviews. *Safety Health Work* 2023; 14: 135-40.[138]

Fiedler J, Eckert T, Wunsch K, Woll A. Key facets to build up eHealth and mHealth interventions to enhance physical activity, sedentary behavior and nutrition in healthy subjects - an umbrella review. *BMC Public Health* 2020; 20: 1605.[139]

Fishwick D, Carroll C, McGregor M, et al. Smoking cessation in the workplace. *Occup Med* 2013; 63: 526-36.[140]

Gelius P, Messing S, Goodwin L, Schow D, Abu-Omar K. What are effective policies for promoting physical activity? A systematic review of reviews. *Prev Med Rep* 2020; 18: 101095.[141]

Goldberg SB, Riordan KM, Sun S, Davidson RJ. The empirical status of mindfulness-based interventions: A systematic review of 44 meta-analyses of randomized controlled trials. *Perspect Psychol Sci* 2022; 17: 108-30.[142]

Hilton, LG, Marshall, NJ, Motalaa A, Taylor SL, Miake-Lye IM, Baxi S, Shanman RM, Sollowayg MR, Beroes JM, Hempel S. Mindfulness meditation for workplace wellness. Work 2019; 63: 205-18.[143]

Hoare E, Collins S, Marx W, et al. Universal depression prevention: An umbrella review of meta-analyses. *J Psychiatr Res* 2021; 144: 483-93.[144]

Kajee N, Montero-Marin J, Saunders KEA, Myall K, Harriss E, Kuyken W. Mindfulness training in healthcare professions: A scoping review of systematic reviews. *Med Educ* 2024; 58: 671-86.[145]

Kalani SD, Azadfallah P, Oreyzi H, Adibi P. Interventions for physician burnout: A systematic review of systematic reviews. *Int J Prev Med* 2018; 9: 81.[146]

Ketelaars E, Gaudin C, Flandin S, Poizat G. Resilience training for critical situation management. An umbrella and a systematic literature review. *Safety Sci* 2024; 170: 106311.[147]

La Torre G, Leggieri PF, Cocchiara RA, et al. Mindfulness as a tool for reducing stress in healthcare professionals: An umbrella review. *Work* 2022; 73: 819-29.[148]

Lam K, Baurecht H, Pahmeier K, et al. How effective and how expensive are interventions to reduce sedentary behavior? An umbrella review and meta-analysis. *Obes Rev* 2022; 23: e13422.[43]

Miguel C, Amarnath A, Akhtar A, et al. Universal, selective and indicated interventions for supporting mental health at the workplace: an umbrella review of meta-analyses. *Occup Environ Med* 2023; 80: 225-36.[149]

Nguyen P, Le LK, Nguyen D, Gao L, Dunstan DW, Moodie M. The effectiveness of sedentary behaviour interventions on sitting time and screen time in children and adults: an umbrella review of systematic reviews. *Int J Behav Nutr Phys Act* 2020; 17: 117.[150]

Proper KI, van Oostrom SH. The effectiveness of workplace health promotion interventions on physical and mental health outcomes - a systematic review of reviews. *Scand J Work Environ Health* 2019; 45: 546-59.[151]

Schliemann D, Woodside JV. The effectiveness of dietary workplace interventions: a systematic review of systematic reviews. *Public Health Nutr* 2019; 22: 942-55.[152]

Schröer S, Haupt J, Pieper C. Evidence-based lifestyle interventions in the workplace—an overview. *Occup Med* 2014; 64: 8-12.[153]

Sowah D, Boyko R, Antle D, Miller L, Zakhary M, Straube S. Occupational interventions for the prevention of back pain: Overview of systematic reviews. *J Safety Res* 2018; 66: 39-59.[154]

Storen PG, Gronningsater H. Do worksite health promotion programs (WHPP) influence presenteeism among employees? A systematic review. *Work* 2024; 77: 85-102.[155]

Teufer B, Ebenberger A, Affengruber L, et al. Evidence-based occupational health and safety interventions: a comprehensive overview of reviews. *BMJ Open* 2019; 9:e032528.[156]

Tomori C, Hernandez-Cordero S, Busath N, Menon P, Perez-Escamilla R. What works to protect, promote and support breastfeeding on a large scale: A review of reviews. *Maternal Child Nutr* 2022; 18: e13344.[157]

Turon H, Bezzina A, Lamont H, et al. Interventions in the workplace to reduce risk factors for noncommunicable diseases: an umbrella review of systematic reviews of effectiveness. *J Occup Health* 2024; 66: 1.[158]

Waddell A, Kunstler B, Lennox A, et al. How effective are interventions in optimizing workplace mental health and well-being? A scoping review of reviews and evidence map. *Scand J Work Environ Health* 2023; 49: 235-48.[159]

White MI, Dionne CE, Wärje O, et al. Physical activity and exercise interventions in the workplace impacting work outcomes: A stakeholder-centered best evidence synthesis of systematic reviews. *Int J Occup Environ Med* 2016; 7: 61-74.[160]

Wnuk K, Switalski J, Tatara T, et al. Workplace interventions for type 2 diabetes mellitus prevention - an umbrella review. *Curr Diab Rep* 2023; 23: 293-304.[161]

Wolfenden L, Barnes C, Lane C, et al. Consolidating evidence on the effectiveness of interventions promoting fruit and vegetable consumption: an umbrella review. *Int J Behav Nutr Phys Act* 2021; 18: 11.[162]

Wu JY, Li H, Shuai JK, He Y, Li PC. Evidence summary on the non-pharmacological management of sleep disorders in shift workers. *Sleep Breath* 2024; 28: 909-18.[163]

Zhang D, Lee EKP, Mak ECW, Ho CY, Wong SYS. Mindfulness-based interventions: an overall review. *Br Med Bull* 2021; 138: 41-57.[164]

Zhang XJ, Song Y, Jiang T, Ding N, Shi TY. Interventions to reduce burnout of physicians and nurses: An overview of systematic reviews and meta-analyses. *Medicine* 2020; 99: e20992.[165]

References

1. Shea BJ, Reeves BC, Wells G, et al. AMSTAR 2: a critical appraisal tool for systematic reviews that include randomised or non-randomised studies of healthcare interventions, or both. *BMJ* 2017; 358: j4008.
2. van Vilsteren M, van Oostrom SH, de Vet HC, Franche RL, Boot CR, Anema JR. Workplace interventions to prevent work disability in workers on sick leave. *Cochrane Database Syst Rev* 2015; 2015: CD006955.
3. Fusar-Poli P, Radua J. Ten simple rules for conducting umbrella reviews. *Evid Based Ment Health* 2018; 21: 95-100.
4. Pollock M, Fernandes RM, Becker LA, Pieper D, Hartling L. Overviews of reviews. In: Higgins JPT, Thomas J, Chandler J, et al., eds. Cochrane Handbook for Systematic Reviews of Interventions: Cochrane, 2022.
5. Abdul Halim NSS, Ripin ZM, Ridzwan MIZ. Efficacy of interventions in reducing the risks of work-related musculoskeletal disorders among healthcare workers - A systematic review and meta-analysis. *Workplace Health Safety* 2023; 71: 557-76.
6. Clari M, Albanesi B, Comoretto RI, et al. Effectiveness of interventions to increase healthcare workers' adherence to vaccination against vaccine-preventable diseases: a systematic review and meta-analysis, 1993 to 2022. *Euro Surveillance* 2024; 29: 9.
7. Coenen P, Robroek SJW, van der Beek AJ, et al. Socioeconomic inequalities in effectiveness of and compliance to workplace health promotion programs: an individual participant data (IPD) meta-analysis. *Int J Behav Nutr Phys Act* 2020; 17: 112.
8. Compernolle S, DeSmet A, Poppe L, et al. Effectiveness of interventions using self-monitoring to reduce sedentary behavior in adults: a systematic review and meta-analysis. *Int J Behav Nutr Phys Act* 2019; 16: 63.
9. Indrayani NLD, Kao CY, Suyasa I, Padmalatha KMS, Chang JH, Wang CJ. Effectiveness of exercise programs to reduce low back pain among nurses and nursing assistants: A systematic review and meta-analysis. *J Safety Res* 2024; 89: 312-21.
10. de Sevilla GGP, Vicente-Arche FC, Thuissard IJ, Barcelo O, Perez-Ruiz M. Effectiveness of Workplace Exercise Interventions on Body Composition: A Systematic Review and Meta-Analysis. *Am J Health Promot* 2021; 35: 1150-61.
11. Fellbaum L, Mojzisch A, Bielefeld L, Benit N, Soellner R. The effectiveness of workplace interventions for the prevention of alcohol use: A meta-analysis. *Addiction* 2023; 118: 2043-61.
12. Lock M, Post D, Dollman J, Parfitt G. Efficacy of theory-informed workplace physical activity interventions: a systematic literature review with meta-analyses. *Health Psychol Rev* 2021; 15: 483-507.
13. Lomas T, Medina JC, Ivtzan I, Rupprecht S, Eiroa-Orosa FJ. A systematic review and meta-analysis of the impact of mindfulness-based interventions on the well-being of healthcare professionals. *Mindfulness* 2019; 10: 1193-216.
14. Pares-Salomon I, Sene-Mir AM, Martin-Bozas F, et al. Effectiveness of workplace interventions with digital elements to reduce sedentary behaviours in office employees: a systematic review and meta-analysis. *Int J Behav Nutr Phys Act* 2024; 21: 41.
15. Park SH, Kim SY. Effectiveness of worksite-based dietary interventions on employees' obesity: a systematic review and meta-analysis. *Nutr Res Pract* 2019; 13: 399-409.
16. Power BT, Kiezebrink K, Allan JL, Campbell MK. Effects of workplace-based dietary and/or physical activity interventions for weight management targeting healthcare professionals: a systematic review of randomised controlled trials. *BMC Obes* 2014; 1: 23.
17. Robroek SJW, Oude Hengel KM, van der Beek AJ, et al. Socio-economic inequalities in the effectiveness of workplace health promotion programmes on body mass index: An individual participant data meta-analysis. *Obes Rev* 2020; 21: e13101.
18. Wang C, Lu EY, Sun W, Chang JR, Tsang HWH. Effectiveness of interventions on sedentary behaviors in office workers: a systematic review and meta-analysis. *Public health* 2024; 230: 45-51.

19. Barger LK, Runyon MS, Renn ML, et al. Effect of fatigue training on safety, fatigue, and sleep in emergency medical services personnel and other shift workers: A systematic review and meta-analysis. *Prehosp Emerg Care* 2018; 22: 58-68.
20. Bartlett L, Martin A, Neil AL, et al. A systematic review and meta-analysis of workplace mindfulness training randomized controlled trials. *J Occup Health Psychol* 2019; 24: 108-26.
21. Bellon JA, Conejo-Ceron S, Cortes-Abela C, Pena-Andreu JM, Garcia-Rodriguez A, Moreno-Peral P. Effectiveness of psychological and educational interventions for the prevention of depression in the workplace: A systematic review and meta-analysis. *Scand J Work Environ Health* 2019; 45: 324-32.
22. Bezzina A, Clarke ED, Ashton L, Watson T, James CL. Workplace Health Promotion Programs Targeting Smoking, Nutrition, Physical Activity, and Obesity in Men: A Systematic Review and Meta-Analysis of Randomized Controlled Trials. *Health Educ Behav* 2024; 51: 113-27.
23. Burn NL, Weston M, Maguire N, Atkinson G, Weston KL. Effects of workplace-based physical activity interventions on cardiorespiratory fitness: A systematic review and meta-analysis of controlled trials. *Sports Med* 2019; 49: 1255-74.
24. Cahill K, Lancaster T. Workplace interventions for smoking cessation. *Cochrane Database Syst Rev* 2014; 2014: CD003440.
25. Caputo EL, Feter N, Alt R, da Silva MC. How do different interventions impact stair climbing? A systematic review and meta-analysis. *Global Health Prom* 2022: 17579759221093388.
26. Carolan S, Harris PR, Cavanagh K. Improving employee well-being and effectiveness: Systematic review and meta-analysis of web-based psychological interventions delivered in the workplace. *J Med Internet Res* 2017; 19: e271.
27. Chen X, Coombes BK, Sjogaard G, Jun D, O'Leary S, Johnston V. Workplace-based interventions for neck pain in office workers: Systematic review and meta-analysis. *Phys Ther* 2018; 98: 40-62.
28. Chu AH, Ng SH, Tan CS, Win AM, Koh D, Muller-Riemenschneider F. A systematic review and meta-analysis of workplace intervention strategies to reduce sedentary time in white-collar workers. *Obes Rev* 2016; 17: 467-81.
29. Cochrane A, Higgins NM, FitzGerald O, et al. Early interventions to promote work participation in people with regional musculoskeletal pain: a systematic review and meta-analysis. *Clin Rehabil* 2017; 31: 1466-81.
30. Eisele-Metzger A, Schoser DS, Klein MD, et al. Interventions for preventing back pain among office workers - a systematic review and network meta-analysis. *Scand J Work Environ Health* 2023; 49: 5-22.
31. Fendel JC, Burkle JJ, Goritz AS. Mindfulness-based interventions to reduce burnout and stress in physicians: A systematic review and meta-analysis. *Acad Med* 2021; 96: 751-64.
32. Fitzpatrick-Lewis D, Ali MU, Horvath S, Nagpal S, Ghanem S, Sherifali D. Effectiveness of workplace interventions to reduce the risk for type 2 diabetes: A systematic review and meta-Analysis. *Can J Diabetes* 2022; 46: 84-98.
33. Freak-Poli R, Cumpston M, Albarqouni L, Clemes SA, Peeters A. Workplace pedometer interventions for increasing physical activity. *Cochrane Database Syst Rev* 2020; 7: CD009209.
34. Frutiger M, Borotkanics R. Systematic review and meta-analysis suggest strength training and workplace modifications may reduce neck pain in office workers. *Pain Pract* 2021; 21: 100-31.
35. Hayden JA, Ellis J, Ogilvie R, Malmivaara A, van Tulder MW. Exercise therapy for chronic low back pain. *Cochrane Database Syst Rev* 2021; 9: CD009790.
36. Heckenberg RA, Eddy P, Kent S, Wright BJ. Do workplace-based mindfulness meditation programs improve physiological indices of stress? A systematic review and meta-analysis. *J Psychosom Res* 2018; 114: 62-71.
37. Hulls PM, Richmond RC, Martin RM, Chavez-Ugalde Y, de Vocht F. Workplace interventions that aim to improve employee health and well-being in male-dominated industries: a systematic review. *Occup Environ Med* 2022; 79: 77-87.
38. Jones LB, Jadhakhan F, Falla D. The influence of exercise on pain, disability and quality of life in office workers with chronic neck pain: A systematic review and meta-analysis. *Appl Ergon* 2024; 117: 104216.

39. Jung J, Cho I. Promoting Physical Activity and Weight Loss With mHealth Interventions Among Workers: Systematic Review and Meta-analysis of Randomized Controlled Trials. *JMIR Mhealth Uhealth* 2022; 10: e30682.
40. Karo M, Simorangkir L, Daryanti Saragih I, Suarilah I, Tzeng HM. Effects of mindfulness-based interventions on reducing psychological distress among nurses: A systematic review and meta-analysis of randomized controlled trials. *J Nurs Scholarsh* 2024; 56: 319-30.
41. Kunzler AM, Chmitorz A, Rothke N, et al. Interventions to foster resilience in nursing staff: A systematic review and meta-analyses of pre-pandemic evidence. *Int J Nurs Stud* 2022; 134: 104312.
42. Ladino MDM, Bolanos C, Ramirez VAC, et al. Effects of internet-based, psychosocial, and early medical interventions on professional burnout in health care workers: Systematic literature review and meta-analysis. *Internet Interv* 2023; 34: 100682.
43. Lam K, Baurecht H, Pahmeier K, et al. How effective and how expensive are interventions to reduce sedentary behavior? An umbrella review and meta-analysis. *Obes Rev* 2022; 23: e13422.
44. Lee HF, Kuo CC, Chien TW, Wang YR. A meta-Analysis of the effects of coping strategies on reducing nurse burnout. *Appl Nurs Res* 2016; 31: 100-10.
45. Liu H, Kong L, Sun Q, Ma X. The effects of mindfulness-based interventions on nurses' anxiety and depression: A meta-analysis. *Nurs Open* 2023; 10: 3622-34.
46. Lomas T, Medina JC, Ivtzan I, Rupprecht S, Eiroa-Orosa FJ. Mindfulness-based interventions in the workplace: An inclusive meta-analysis of their impact upon wellbeing. *J Positive Psychol* 2019; 14: 625-40.
47. Louw S, Makwela S, Manas L, Meyer L, Terblanche D, Brink Y. Effectiveness of exercise in office workers with neck pain: A systematic review and meta-analysis. *S Afr J Physiother* 2017; 73: 392.
48. Lowry V, Desjardins-Charbonneau A, Roy JS, et al. Efficacy of workplace interventions for shoulder pain: A systematic review and meta-analysis. *J Rehabil Med* 2017; 49: 529-42.
49. Lu YF, Petersen K. Effectiveness of psychological skills training for police personnel: a meta-analysis. *Occup Environ Med* 2023; 80: 590-8.
50. Luger T, Maher CG, Rieger MA, Steinhilber B. Work-break schedules for preventing musculoskeletal symptoms and disorders in healthy workers. *Cochrane Database Syst Rev* 2019; 7: CD012886.
51. Martin-Gill C, Barger LK, Moore CG, et al. Effects of napping during shift work on sleepiness and performance in emergency medical services personnel and similar shift workers: A systematic review and meta-analysis. *Prehosp Emerg Care* 2018; 22: 47-57.
52. Merom D, Stanaway F, Gebel K, et al. Supporting active ageing before retirement: a systematic review and meta-analysis of workplace physical activity interventions targeting older employees. *BMJ open* 2021; 11: e045818.
53. Michaelsen MM, Graser J, Onescheit M, et al. Mindfulness-based and mindfulness-informed interventions at the workplace: A systematic review and meta-regression analysis of RCTs. *Mindfulness* 2023: 1-34.
54. Montano D, Hoven H, Siegrist J. Effects of organisational-level interventions at work on employees' health: a systematic review. *BMC Public Health* 2014; 14: 135.
55. Mulchandani R, Chandrasekaran AM, Shivashankar R, et al. Effect of workplace physical activity interventions on the cardio-metabolic health of working adults: systematic review and meta-analysis. *Int J Behav Nutr Phys Act* 2019; 16: 134.
56. Nigatu YT, Huang J, Rao S, Gillis K, Merali Z, Wang J. Indicated prevention interventions in the workplace for depressive symptoms: A systematic review and meta-analysis. *Am J Prev Med* 2019; 56: e23-e33.
57. Panagioti M, Panagopoulou E, Bower P, et al. Controlled Interventions to Reduce Burnout in Physicians: A Systematic Review and Meta-analysis. *JAMA Int Med* 2017; 177: 195-205.
58. Panchbhaya A, Baldwin C, Gibson R. Improving the dietary intake of health care workers through workplace dietary interventions: A systematic review and meta-analysis. *Adv Nutrition* 2022; 13: 595-620.

59. Parry SP, Coenen P, Shrestha N, O'Sullivan PB, Maher CG, Straker LM. Workplace interventions for increasing standing or walking for decreasing musculoskeletal symptoms in sedentary workers. *Cochrane Database Syst Rev* 2019; 2019: 11.
60. Paudel NR, Adhikari BA, Prakash KC, Kyronlahti S, Nygard CH, Neupane S. Effectiveness of interventions on the stress management of schoolteachers: a systematic review and meta-analysis. *Occup Environ Med* 2022; 79: 477-85.
61. Peachey MM, Richardson J, A VT, Dal-Bello Haas V, Gravesande J. Environmental, behavioural and multicomponent interventions to reduce adults' sitting time: a systematic review and meta-analysis. *Br J Sports Med* 2020; 54: 315-25.
62. Penalvo JL, Sagastume D, Mertens E, et al. Effectiveness of workplace wellness programmes for dietary habits, overweight, and cardiometabolic health: a systematic review and meta-analysis. *Lancet Public Health* 2021; 6: e648-e60.
63. Petrie K, Crawford J, Baker STE, et al. Interventions to reduce symptoms of common mental disorders and suicidal ideation in physicians: a systematic review and meta-analysis. *Lancet Psychiatry* 2019; 6: 225-34.
64. Phillips EA, Gordeev VS, Schreyogg J. Effectiveness of occupational e-mental health interventions: a systematic review and meta-analysis of randomized controlled trials. *Scand J Work Environ Health* 2019; 45: 560-76.
65. Picon SPB, Batista GA, Pitangui ACR, de Araujo RC. Effects of Workplace-Based Intervention for Shoulder Pain: A Systematic Review and Meta-analysis. *J Occup Rehabil* 2021; 31: 243-62.
66. Prieske O, Dalager T, Herz M, et al. Effects of Physical Exercise Training in the Workplace on Physical Fitness: A Systematic Review and Meta-analysis. *Sports Med* 2019; 49: 1903-21.
67. Prudenzi A, Graham CD, Clancy F, et al. Group-based acceptance and commitment therapy interventions for improving general distress and work-related distress in healthcare professionals: A systematic review and meta-analysis. *J Affect Disord* 2021; 295: 192-202.
68. Reed JL, Prince SA, Elliott CG, et al. Impact of workplace physical activity interventions on physical activity and cardiometabolic health among working-age women: A systematic review and meta-analysis. *Circ Cardiovasc Qual Outcomes* 2017; 10: 2.
69. Richardson AS, Chen C, Sturm R, et al. Obesity prevention interventions and implications for energy balance in the United States and Mexico: A systematic review of the evidence and meta-analysis. *Obesity* 2019; 27: 1390-403.
70. Rongen A, Robroek SJW, van Lenthe FJ, Burdorf A. Workplace health promotion - A meta-analysis of effectiveness. *Am J Prev Med* 2013; 44: 406-15.
71. Ruiz-Fernandez MD, Ortiz-Amo R, Ortega-Galan AM, Ibanez-Masero O, Rodriguez-Salvador MDM, Ramos-Pichardo JD. Mindfulness therapies on health professionals. *Int J Mental Health Nurs* 2020; 29: 127-40.
72. Ruotsalainen JH, Verbeek JH, Marine A, Serra C. Preventing occupational stress in healthcare workers. *Cochrane Database Syst Rev* 2015; 2015: CD002892.
73. Shrestha N, Kukkonen-Harjula KT, Verbeek JH, Ijaz S, Hermans V, Pedisic Z. Workplace interventions for reducing sitting at work. *Cochrane Database Syst Rev* 2018; 12: CD010912.
74. Shrestha A, Karmacharya BM, Khudyakov P, Weber MB, Spiegelman D. Dietary interventions to prevent and manage diabetes in worksite settings: a meta-analysis. *J Occup Health* 2018; 60: 31-45.
75. Slanger TE, Gross JV, Pinger A, et al. Person-directed, non-pharmacological interventions for sleepiness at work and sleep disturbances caused by shift work. *Cochrane Database Syst Rev* 2016; 2016: CD010641.
76. Slemp GR, Jach HK, Chia A, Loton DJ, Kern ML. Contemplative interventions and employee distress: A meta-analysis. *Stress Health* 2019; 35: 227-55.
77. Stratton E, Lampit A, Choi I, et al. Trends in effectiveness of organizational eHealth interventions in addressing employee mental health: Systematic review and meta-analysis. *J Med Internet Res* 2022; 24: e37776.
78. Tamminga SJ, Emal LM, Boschman JS, et al. Individual-level interventions for reducing occupational stress in healthcare workers. *Cochrane Database Syst Rev* 2023; 5: CD002892.

79. Tan L, Petrie K, Deady M, Bryant RA, Harvey SB. Systematic review of first responder post-deployment or post-incident psychosocial interventions. *Occup Med (Lond)* 2022; 72: 160-9.
80. Tang X, Patterson P, MacKenzie-Shalders K, et al. Workplace programmes for supporting breast-feeding: a systematic review and meta-analysis. *Public Health Nutr* 2021; 24: 1501-13.
81. Teichert F, Karner V, Doding R, Saueressig T, Owen PJ, Belavy DL. Effectiveness of exercise interventions for preventing neck pain: A systematic review with meta-analysis of randomized controlled trials. *J Orthop Sports Phys Ther* 2023; 53: 594-609.
82. Verbeek J, Martimo KP, Karppinen J, Kuijer PP, Takala EP, Viikari-Juntura E. Manual material handling advice and assistive devices for preventing and treating back pain in workers: a Cochrane Systematic Review. *Occup Environ Med* 2012; 69: 79-80.
83. Verhagen AP, Bierma-Zeinstra SM, Burdorf A, Stynes SM, de Vet HC, Koes BW. Conservative interventions for treating work-related complaints of the arm, neck or shoulder in adults. *Cochrane Database Syst Rev* 2013; 2013: CD008742.
84. Verweij LM, Coffeng J, van Mechelen W, Proper KI. Meta-analyses of workplace physical activity and dietary behaviour interventions on weight outcomes. *Obes Rev* 2011; 12: 406-29.
85. Virga D, Rusu A, Pap Z, Maricutoiu L, Tisu L. Effectiveness of strengths use interventions in organizations: A pre-registered meta-analysis of controlled trials. *Applied Psychology* 2022; 72: 1653-93.
86. Vonderlin R, Biermann M, Bohus M, Lyssenko L. Mindfulness-based programs in the workplace: a meta-analysis of randomized controlled trials. *Mindfulness* 2020; 11: 1579-98.
87. West CP, Dyrbye LN, Erwin PJ, Shanafelt TD. Interventions to prevent and reduce physician burnout: a systematic review and meta-analysis. *Lancet* 2016; 388: 2272-81.
88. Yuvaraj K, Eliyas SK, Gokul S, Manikandanesan S. Effectiveness of workplace intervention for reducing alcohol consumption: a systematic review and meta-analysis. *Alcohol Alcohol* 2019; 54: 264-71.
89. Zhang M, Murphy B, Cabanilla A, Yidi C. Physical relaxation for occupational stress in healthcare workers: A systematic review and network meta-analysis of randomized controlled trials. *J Occup Health* 2021; 63: e12243.
90. Zhou L, Deng X, Xu M, et al. The effects of active workstations on reducing work-specific sedentary time in office workers: a network meta-analysis of 23 randomized controlled trials. *Int J Behav Nutr Phys Act* 2023; 20: 92.
91. Zhou L, Deng X, Guo K, et al. Effectiveness of multicomponent interventions in office-based workers to mitigate occupational sedentary behavior: Systematic review and meta-analysis. *JMIR Public Health Surveill* 2023; 9: e44745.
92. Agarwal S, Steinmaus C, Harris-Adamson C. Sit-stand workstations and impact on low back discomfort: a systematic review and meta-analysis. *Ergonomics* 2018; 61: 538-52.
93. Andrews KL, Gallagher S, Herring MP. The effects of exercise interventions on health and fitness of firefighters: A meta-analysis. *Scand J Med Sci Sports* 2019; 29: 780-90.
94. Antipas H, Tamplin J, Vieira Sousa T, Baker FA. Interventions for mitigating occupational stress for professional dementia caregivers in residential aged care: A systematic review with meta-analysis. *Dementia* 2024; 23: 292-311.
95. Ayaz D, Asi E, Meydanlioglu A, Oncel S. Effectiveness of smoking cessation interventions in the workplace: A systematic review and meta-analysis. *Am J Ind Med* 2024; 67: 712-22.
96. Beames JR, Spanos S, Roberts A, et al. Intervention programs targeting the mental health, professional burnout, and/or wellbeing of school teachers: Systematic review and meta-analyses. *Educ Psychol Rev* 2023; 35: 26.
97. Burton A, Burgess C, Dean S, Koutsopoulou GZ, Hugh-Jones S. How effective are mindfulness-based interventions for reducing stress among healthcare professionals? A systematic review and meta-analysis. *Stress Health* 2017; 33: 3-13.
98. Busireddy KR, Miller JA, Ellison K, Ren V, Qayyum R, Panda M. Efficacy of interventions to reduce resident physician burnout: A systematic review. *J Grad Med Educn* 2017; 9: 294-301.

99. De Simone S, Vargas M, Servillo G. Organizational strategies to reduce physician burnout: a systematic review and meta-analysis. *Aging Clin Exp Res* 2021; 33: 883-94.
100. Dharmawardene M, Givens J, Wachholtz A, Makowski S, Tjia J. A systematic review and meta-analysis of meditative interventions for informal caregivers and health professionals. *BMJ Support Palliat Care* 2016; 6: 160-9.
101. Dreison KC, Luther L, Bonfils KA, Sliter MT, McGrew JH, Salyers MP. Job burnout in mental health providers: A meta-analysis of 35 years of intervention research. *J Occup Health Psychol* 2018; 23: 18-30.
102. Guillaumie L, Boiral O, Champagne J. A mixed-methods systematic review of the effects of mindfulness on nurses. *J Adv Nurs* 2017; 73: 1017-34.
103. Haslam A, Tuia J, Miller SL, Prasad V. Systematic review and meta-analysis of randomized trials testing interventions to reduce physician burnout. *Am J Med* 2024; 137: 249-57 e1.
104. Hutchinson AD, Wilson C. Improving nutrition and physical activity in the workplace: a meta-analysis of intervention studies. *Health Promot Int* 2012; 27: 238-49.
105. Kang J, Noh W, Lee Y. Sleep quality among shift-work nurses: A systematic review and meta-analysis. *Appl Nurs Res* 2020; 52: 151227.
106. Klingbeil DA, Renshaw TL. Mindfulness-based interventions for teachers: A meta-analysis of the emerging evidence base. *Sch Psychol Q* 2018; 33: 501-11.
107. Kröll C, Doebler P, Nüesch S. Meta-analytic evidence of the effectiveness of stress management at work. *Eur J Work Organ Psychol* 2017; 26: 677-93.
108. La Torre G, Tiberio G, Sindoni A, Dorelli B, Cammalleri V. Smoking cessation interventions on health-care workers: a systematic review and meta-analysis. *PeerJ* 2020; 8: e9396.
109. Leeks KD, Hopkins DP, Soler RE, Aten A, Chattopadhyay SK, Task Force on Community Preventive. Worksite-based incentives and competitions to reduce tobacco use. A systematic review. *Am J Prev Med* 2010; 38: S263-74.
110. Li Y, Wang X, Chen J, Chi-Kin Lee J, Yan Z, Li J. The intervention effects on teacher well-being: A three-level meta-analysis. *Educ Psychol Rev* 2024; 36: 129.
111. Liu JJW, Ein N, Gervasio J, Battaion M, Reed M, Vickers K. Comprehensive meta-analysis of resilience interventions. *Clin Psychol Rev* 2020; 82: 101919.
112. Maricutoiu LP, Sava FA, Butta O. The effectiveness of controlled interventions on employees' burnout: A meta-analysis. *J Occup Organ Psychol* 2016; 89: 1-27.
113. Moreira-Silva I, Teixeira PM, Santos R, Abreu S, Moreira C, Mota J. The effects of workplace physical activity programs on musculoskeletal pain: A systematic review and meta-analysis. *Workplace Health Safety* 2016; 64: 210-22.
114. Neuhaus M, Eakin EG, Straker L, et al. Reducing occupational sedentary time: a systematic review and meta-analysis of evidence on activity-permissive workstations. *Obes Rev* 2014; 15: 822-38.
115. Ochentel O, Humphrey C, Pfeifer K. Efficacy of exercise therapy in persons with burnout. A systematic review and meta-analysis. *J Sports Sci Med* 2018; 17: 475-84.
116. Oye-Somefun A, Azizi Z, Ardern CI, Rotondi MA. A systematic review and meta-analysis of the effect of treadmill desks on energy expenditure, sitting time and cardiometabolic health in adults. *BMC Public Health* 2021; 21: 2082.
117. Patterson GT, Chung IW, Swan PW. Stress management interventions for police officers and recruits: a meta-analysis. *Journal of Experimental Criminology* 2014; 10: 487-513.
118. Podrekar N, Kozinc Z, Sarabon N. Effects of cycle and treadmill desks on energy expenditure and cardiometabolic parameters in sedentary workers: review and meta-analysis. *Int J Occup Saf Ergon* 2021; 27: 728-36.
119. Ramachandran HJ, Bin Mahmud MS, Rajendran P, Jiang Y, Cheng L, Wang W. Effectiveness of mindfulness-based interventions on psychological well-being, burnout and post-traumatic stress disorder among nurses: A systematic review and meta-analysis. *J Clin Nurs* 2023; 32: 2323-38.
120. Reeve A, Tickle A, Moghaddam N. Are acceptance and commitment therapy-based interventions effective for reducing burnout in direct-care staff? A systematic review and meta-analysis. *Mental Health Rev J* 2018; 23: 133-155.

121. Regehr C, Glancy D, Pitts A, LeBlanc VR. Interventions to reduce the consequences of stress in physicians: a review and meta-analysis. *J Nerv Ment Dis* 2014; 202: 353-9.
122. Spinelli C, Wisener M, Khoury B. Mindfulness training for healthcare professionals and trainees: A meta-analysis of randomized controlled trials. *J Psychosom Res* 2019; 120: 29-38.
123. Stephenson A, McDonough SM, Murphy MH, Nugent CD, Mair JL. Using computer, mobile and wearable technology enhanced interventions to reduce sedentary behaviour: a systematic review and meta-analysis. *Int J Behav Nutr Phys Act* 2017; 14: 105.
124. Sun W, Zhang H, Lv C, Tang L, Tian S. Comparative efficacy of 12 non-drug interventions on non-specific chronic low back pain in nurses: A systematic review and network meta-analysis. *J Back Musculoskelet Rehabil* 2021; 34: 499-510.
125. Tan L, Wang MJ, Modini M, et al. Preventing the development of depression at work: a systematic review and meta-analysis of universal interventions in the workplace. *BMC Med* 2014; 12: 74.
126. Uslu D, Marcus J, Kisbu-Sakarya Y. Toward optimized effectiveness of employee training programs. *J Pers Psychol* 2022; 21: 49-65.
127. van Heijster H, Boot CRL, Robroek SJW, et al. The effectiveness of workplace health promotion programs on self-perceived health of employees with a low socioeconomic position: An individual participant data meta-analysis. *SSM Popul Health* 2021; 13: 100743.
128. Vanhove AJ, Herian MN, Perez ALU, Harms PD, Lester PB. Can resilience be developed at work? A meta-analytic review of resilience-building programme effectiveness. *J Occup Organ Psychol* 2016; 89: 278-307.
129. Wang R, Gu X, Zhang Y, Luo K, Zeng X. Loving-kindness and compassion meditations in the workplace: A meta-analysis and future prospects. *Stress Health* 2024; 40: e3273.
130. Wasson RS, Barratt C, O'Brien WH. Effects of mindfulness-based interventions on self-compassion in health care professionals: a meta-analysis. *Mindfulness* 2020; 11: 1914-34.
131. Witt K, Milner A, Allisey A, Davenport L, LaMontagne AD. Effectiveness of suicide prevention programs for emergency and protective services employees: A systematic review and meta-analysis. *Am J Ind Med* 2017; 60: 394-407.
132. Yu F, Chu G, Yeh T, Fernandez R. Effects of interventions to promote resilience in nurses: A systematic review. *Int J Nurs Stud* 2024; 157: 104825.
133. Zarate K, Maggin DM, Passmore A. Meta-analysis of mindfulness training on teacher well-being. *Psychol School* 2019; 56: 1700-15.
134. Adnan NBB, Dafny HA, Baldwin C, et al. What are the solutions for well-being and burn-out for healthcare professionals? An umbrella realist review of learnings of individual-focused interventions for critical care. *BMJ open* 2022; 12: e060973.
135. Antony J, Brar R, Khan PA, et al. Interventions for the prevention and management of occupational stress injury in first responders: a rapid overview of reviews. *Syst Rev* 2020; 9: 121.
136. Cleland CL, Jones S, Moeinaddini M, et al. Complex interventions to reduce car use and change travel behaviour: An umbrella review. *J Transport Health* 2023; 31: 101652.
137. Di Mario S, Cocchiara RA, La Torre G. The use of yoga and mindfulness-based interventions to reduce stress and burnout in healthcare workers: An umbrella review. *Altern Ther Health Med* 2023; 29: 29-35.
138. Fadel M, Roquelaure Y, Descatha A. Interventions on well-being, occupational health, and aging of healthcare Workers: A Scoping Review of Systematic Reviews. *Safety Health Work* 2023; 14: 135-40.
139. Fiedler J, Eckert T, Wunsch K, Woll A. Key facets to build up eHealth and mHealth interventions to enhance physical activity, sedentary behavior and nutrition in healthy subjects - an umbrella review. *BMC Public Health* 2020; 20: 1605.
140. Fishwick D, Carroll C, McGregor M, et al. Smoking cessation in the workplace. *Occup Med* 2013; 63: 526-36.
141. Gelius P, Messing S, Goodwin L, Schow D, Abu-Omar K. What are effective policies for promoting physical activity? A systematic review of reviews. *Prev Med Rep* 2020; 18: 101095.

142. Goldberg SB, Riordan KM, Sun S, Davidson RJ. The empirical status of mindfulness-based interventions: A systematic review of 44 meta-analyses of randomized controlled trials. *Perspect Psychol Sci* 2022; 17: 108-30.
143. Hilton, LG, Marshall, NJ, Motalaa A, Taylor SL, Miake-Lye IM, Baxi S, Shanman RM, Sollowayg MR, Beroes JM, Hempel S. Mindfulness meditation for workplace wellness. Work 2019; 63: 205-18.
144. Hoare E, Collins S, Marx W, et al. Universal depression prevention: An umbrella review of meta-analyses. *J Psychiatr Res* 2021; 144: 483-93.
145. Kajee N, Montero-Marin J, Saunders KEA, Myall K, Harriss E, Kuyken W. Mindfulness training in healthcare professions: A scoping review of systematic reviews. *Med Educ* 2024; 58: 671-86.
146. Kalani SD, Azadfallah P, Oreyzi H, Adibi P. Interventions for physician burnout: A systematic review of systematic reviews. *Int J Prev Med* 2018; 9: 81.
147. Ketelaars E, Gaudin C, Flandin S, Poizat G. Resilience training for critical situation management. An umbrella and a systematic literature review. *Safety Science* 2024; 170: 106311.
148. La Torre G, Leggieri PF, Cocchiara RA, et al. Mindfulness as a tool for reducing stress in healthcare professionals: An umbrella review. *Work* 2022; 73: 819-29.
149. Miguel C, Amarnath A, Akhtar A, et al. Universal, selective and indicated interventions for supporting mental health at the workplace: an umbrella review of meta-analyses. *Occup Environ Med* 2023; 80: 225-36.
150. Nguyen P, Le LK, Nguyen D, Gao L, Dunstan DW, Moodie M. The effectiveness of sedentary behaviour interventions on sitting time and screen time in children and adults: an umbrella review of systematic reviews. *Int J Behav Nutr Phys Act* 2020; 17: 117.
151. Proper KI, van Oostrom SH. The effectiveness of workplace health promotion interventions on physical and mental health outcomes - a systematic review of reviews. *Scand J Work Environ Health* 2019; 45: 546-59.
152. Schliemann D, Woodside JV. The effectiveness of dietary workplace interventions: a systematic review of systematic reviews. *Public Health Nutr* 2019; 22: 942-55.
153. Schröer S, Haupt J, Pieper C. Evidence-based lifestyle interventions in the workplace - an overview. *Occupational Medicine* 2014; 64: 8-12.
154. Sowah D, Boyko R, Antle D, Miller L, Zakhary M, Straube S. Occupational interventions for the prevention of back pain: Overview of systematic reviews. *J Safety Res* 2018; 66: 39-59.
155. Storen PG, Gronningsater H. Do worksite health promotion programs (WHPP) influence presenteeism among employees? A systematic review. *Work* 2024; 77: 85-102.
156. Teufer B, Ebenberger A, Affengruber L, et al. Evidence-based occupational health and safety interventions: a comprehensive overview of reviews. *BMJ open* 2019; 9: e032528.
157. Tomori C, Hernandez-Cordero S, Busath N, Menon P, Perez-Escamilla R. What works to protect, promote and support breastfeeding on a large scale: A review of reviews. *Maternal Child Nutr* 2022; 18 Suppl 3: e13344.
158. Turon H, Bezzina A, Lamont H, et al. Interventions in the workplace to reduce risk factors for noncommunicable diseases: an umbrella review of systematic reviews of effectiveness. *J Occup Health* 2024; 66: 1.
159. Waddell A, Kunstler B, Lennox A, et al. How effective are interventions in optimizing workplace mental health and well-being? A scoping review of reviews and evidence map. *Scand J Work Environ Health* 2023; 49: 235-48.
160. White MI, Dionne CE, Wärje O, et al. Physical activity and exercise interventions in the workplace impacting work outcomes: A stakeholder-centered best evidence synthesis of systematic reviews. *Int J Occup Environ Med* 2016; 7: 61-74.
161. Wnuk K, Switalski J, Tatara T, et al. Workplace interventions for type 2 diabetes mellitus prevention - an umbrella review. *Curr Diabetes Rep* 2023; 23: 293-304.
162. Wolfenden L, Barnes C, Lane C, et al. Consolidating evidence on the effectiveness of interventions promoting fruit and vegetable consumption: an umbrella review. *Int J Behav Nutr Phys Act* 2021; 18: 11.

163. Wu JY, Li H, Shuai JK, He Y, Li PC. Evidence summary on the non-pharmacological management of sleep disorders in shift workers. *Sleep Breath* 2024; 28: 909-18.
164. Zhang D, Lee EKP, Mak ECW, Ho CY, Wong SYS. Mindfulness-based interventions: an overall review. *Br Med Bull* 2021; 138: 41-57.
165. Zhang XJ, Song Y, Jiang T, Ding N, Shi TY. Interventions to reduce burnout of physicians and nurses: An overview of systematic reviews and meta-analyses. *Medicine* 2020; 99: e20992.

RETRIEVED SYSTEMATIC REVIEWS WITHOUT META-ANALYSIS ON WORKPLACE HEALTH PROMOTION

166. Aas RW, Tuntland H, Holte KA, et al. Workplace interventions for neck pain in workers. *Cochrane Database Syst Rev* 2011; 2011: CD008160.
167. Abdin S, Welch RK, Byron-Daniel J, Meyrick J. The effectiveness of physical activity interventions in improving well-being across office-based workplace settings: a systematic review. *Public Health* 2018: 70-6.
168. Akanbi MO, Iroz CB, O'Dwyer LC, Rivera AS, McHugh MC. A systematic review of the effectiveness of employer-led interventions for drug misuse. *J Occup Health* 2020; 62: e12133.
169. Albanesi B, Piredda M, Bravi M, et al. Interventions to prevent and reduce work-related musculoskeletal injuries and pain among healthcare professionals. A comprehensive systematic review of the literature. *J Safety Res* 2022; 82: 124-43.
170. Alkhawaldeh JMA, Soh KL, Mukhtar FBM, Peng OC, Anshasi HA. Stress management interventions for intensive and critical care nurses: A systematic review. *Nurs Crit Care* 2020; 25: 84-92.
171. Allan J, Querstret D, Banas K, de Bruin M. Environmental interventions for altering eating behaviours of employees in the workplace: a systematic review. *Obes Rev* 2017; 18: 214-26.
172. al-Tamimi K, van Herwerden L, Abdul M, Utter J. Hospital-based food environment interventions to improve workforce dietary behaviour: A systematic literature review. *Am J Lifestyle Med* 2023; online first.
173. Alzailai N, Barriball KL, Xyrichis A. Impact of and mitigation measures for burnout in frontline healthcare workers during disasters: A mixed-method systematic review. *Worldviews Evid Based Nurs* 2023; 20: 133-41.
174. Amatori S, Ferri Marini C, Gobbi E, et al. Short high-intensity interval exercise for workplace-based physical activity interventions: A systematic review on feasibility and wffectiveness. *Sports Med* 2023; 53: 887-901.
175. Anchors ZG, Arnold R, S DB, Bressington CA, Moreton AE, Moore LJ. Effectiveness of interventions on occupational stress, health and well-being, performance, and job satisfaction for midwives: A systematic mixed methods review. *Women Birth* 2024; 37: 101589.
176. Aneni EC, Roberson LL, Maziak W, et al. A systematic review of internet-based worksite wellness approaches for cardiovascular disease risk management: outcomes, challenges & opportunities. *PLoS One* 2014; 9: e83594.
177. Anger WK, Dimoff JK, Alley L. Addressing health care workers' mental health: A systematic review of evidence-based interventions and current resources. *Am J Public Health* 2024; 114: 213-26.
178. Araujo D, Bartolo A, Fernandes C, Pereira A, Monteiro S. Intervention programs targeting burnout in health professionals: A systematic review. *Iran J Public Health* 2024; 53: 997-1008.
179. Archer WR, Batan MC, Buchanan LR, et al. Promising practices for the prevention and control of obesity in the worksite. *Am J Health Promot* 2011; 25: e12-26.
180. Aryankhesal A, Mohammadibakhsh R, Hamidi Y, et al. Interventions on reducing burnout in physicians and nurses: A systematic review. *Med J Islam Repub Iran* 2019; 33: 77.
181. Asuquo EG, Tighe SM, Bradshaw C. Interventions to reduce work-related musculoskeletal disorders among healthcare staff in nursing homes; An integrative literature review. *Int J Nursing Stud Adv* 2021; 3: 100033.
182. Barati Jozan MM, Ghorbani BD, Khalid MS, Lotfata A, Tabesh H. Impact assessment of e-trainings in occupational safety and health: a literature review. *BMC Public Health* 2023; 23: 1187.

183. Baygi F, Djalalinia S, Qorbani M, Dejman M, Nielsen JB. Lifestyle interventions in the maritime settings: a systematic review. *Environ Health Prev Med* 2020; 25: 10.
184. Berardo L, Gerges C, Wright J, et al. Assessment of burnout prevention and wellness programs for US-based neurosurgical faculty and residents: a systematic review of the literature. *J Neurosurg* 2021; 135: 392-400.
185. Bezzina A, Ashton L, Watson T, James CL. Workplace wellness programs targeting weight outcomes in men: A scoping review. *Obes Rev* 2022; 23: e13410.
186. Billings J, Zhan Yuen Wong N, Nicholls H, et al. Post-incident psychosocial interventions after a traumatic incident in the workplace: a systematic review of current research evidence and clinical guidance. *Eur J Psychotraumatol* 2023; 14: 2281751.
187. Bischoff LL, Otto AK, Hold C, Wollesen B. The effect of physical activity interventions on occupational stress for health personnel: A systematic review. *Int J Nurs Stud* 2019; 97: 94-104.
188. Bordado Skold M, Bayattork M, Andersen LL, Schlunssen V. Psychosocial effects of workplace exercise - A systematic review. *Scand J Work Environ Health* 2019; 45: 533-45.
189. Brand SL, Thompson Coon J, Fleming LE, Carroll L, Bethel A, Wyatt K. Whole-system approaches to improving the health and wellbeing of healthcare workers: A systematic review. *PLoS One* 2017; 12: e0188418.
190. Brassington K, Lomas T. Can resilience training improve well-being for people in high-risk occupations? A systematic review through a multidimensional lens. *J Posit Psychol* 2021; 16: 573-92.
191. Bresesti I, Folgori L, De Bartolo P. Interventions to reduce occupational stress and burn out within neonatal intensive care units: a systematic review. *Occup Environ Med* 2020; 77: 515-9.
192. Breslin FC, Kyle N, Bigelow P, et al. Effectiveness of health and safety in small enterprises: a systematic review of quantitative evaluations of interventions. *J Occup Rehabil* 2010; 20: 163-79.
193. Brierley ML, Chater AM, Smith LR, Bailey DP. The effectiveness of sedentary behaviour reduction workplace interventions on cardiometabolic risk markers: A systematic review. *Sports Med* 2019; 49: 1739-67.
194. Brown HE, Gilson ND, Burton NW, Brown WJ. Does physical activity impact on presenteeism and other indicators of workplace well-being? *Sports Med* 2011; 41: 249-62.
195. Brown SA, Garcia AA, Zuniga JA, Lewis KA. Effectiveness of workplace diabetes prevention programs: A systematic review of the evidence. *Patient Educ Couns* 2018; 101: 1036-50.
196. Burns RJ, Donovan AS, Ackermann RT, Finch EA, Rothman AJ, Jeffery RW. A theoretically grounded systematic review of material incentives for weight loss: implications for interventions. *Ann Behav Med* 2012; 44: 375-88.
197. Cairns J, Bambra C, F.C. H-B, Moore HJ, Summerbell CD. Weighing up the evidence: a systematic review of the effectiveness of workplace interventions to tackle socio-economic inequalities in obesity. *J Public Health* 2014; 37: 659-70.
198. Cann R, Sinnema C, Rodway J, Daly AJ. What do we know about interventions to improve educator wellbeing? A systematic literature review. *J Educ Change* 2024; 25: 231-70.
199. Capodici A, Sanmarchi F, Golinelli D. Effects of meditation for surgeons: A systematic review of the scientific literature. *Annals Surg* 2022; 275: 1074-7.
200. Caponecchia C, Coman RL, Gopaldasani V, Mayland EC, Campbell L. Musculoskeletal disorders in aged care workers: a systematic review of contributing factors and interventions. *Int J Nurs Stud* 2020; 110: 103715.
201. Carter P, Bignardi G, Hollands GJ, Marteau TM. Information-based cues at point of choice to change selection and consumption of food, alcohol and tobacco products: a systematic review. *BMC Public Health* 2018; 18: 418.
202. Chan CW, Perry L. Lifestyle health promotion interventions for the nursing workforce: a systematic review. *J Clin Nurs* 2012; 21: 2247-61.
203. Channak S, Klinsophon T, Janwantanakul P. The effects of chair intervention on lower back pain, discomfort and trunk muscle activation in office workers: a systematic review. *Int J Occup Saf Ergon* 2022; 28: 1722-31.

204. Chmielewski J, Los K, Luczynski W. Mindfulness in healthcare professionals and medical education. *Int J Occup Med Environ Health* 2021; 34: 1-14.
205. Chu AH, Koh D, Moy FM, Muller-Riemenschneider F. Do workplace physical activity interventions improve mental health outcomes? *Occup Med* 2014; 64: 235-45.
206. Claringbold G, Robinson N, Anglim J, Kavadas V, Walker A, Forsyth L. A systematic review of well-being interventions and initiatives for Australian and New Zealand emergency service workers. *Aust J Psychol* 2022; 74: e2123282.
207. Commissaris D, Huysmans MA, Mathiassen SE, Srinivasan D, Koppes LLJ, Hendriksen IJM. Interventions to reduce sedentary behavior and increase physical activity during productive work: a systematic review. *Scand J Work Environ Health* 2016; 42: 181-91.
208. Cocchiara RA, Peruzzo M, Mannocci A, et al. The use of yoga to manage stress and burnout in healthcare workers: A systematic review. *J Clin Med* 2019; 8: 3.
209. Cooklin A, Joss N, Husser E, Oldenburg B. Integrated approaches to occupational health and safety: A systematic review. *Am J Health Promot* 2017; 31: 401-12.
210. Covington L, Banerjee M, Pereira A, Price M. Mindfulness-based interventions for professionals working in end-of-life care: A systematic review of the literature. *J Palliat Care* 2023; 38: 225-38.
211. Crane MM, Halloway S, Walts ZL, et al. Behavioural interventions for CVD risk reduction for blue-collar workers: a systematic review. *J Epidemiol Community Health* 2021; 75: 1236-43.
212. Csizmar GT, Irwin M. Efficacy of weight loss interventions in United States active duty military populations: A systematic review. *Mil Med* 2021; 186: 1093-9.
213. Davis C, Huggins CE, Kleve S, Leung GKW, Bonham MP. Conceptualizing weight management for night shift workers: A mixed-methods systematic review. *Obes Rev* 2024; 25: e13659.
214. Oliveira PNA, da Silva Filho JN, Gurgel JL, Russomano T, Porto F. Effects of exercises performed in the work environment on occupational stress: A systematic review. *J Bodyw Mov Ther* 2023; 35: 182-9.
215. DeChant PF, Acs A, Rhee KB, et al. Effect of organization-directed workplace interventions on physician burnout: A systematic review. *Mayo Clin Proc Innov Qual Outcomes* 2019; 3: 384-408.
216. Demou E, MacLean A, Cheripelli LJ, Hunt K, Gray CM. Group-based healthy lifestyle workplace interventions for shift workers: a systematic review. *Scand J Work Environ Health* 2018; 44: 568-84.
217. de Sevilla GGP, Pinto BS. Effectiveness of workplace mediterranean diet interventions on cardiometabolic risk factors. *Workplace Health Safety* 2022; 70: 73-80.
218. Doody CB, Robertson L, Cox KM, Bogue J, Egan J, Sarma KM. Pre-deployment programmes for building resilience in military and frontline emergency service personnel. *Cochrane Database Syst Rev* 2021; 12: CD013242.
219. Dugdale Z, Eiter B, Menéndez CC, Wong I, Bauerle T. Findings from a systematic review of fatigue interventions: What's (not) being tested in mining and other industrial environments. *Am J Ind Med* 2022; 65: 248-61.
220. Durand-Moreau Q, Jackson T, Deibert D, Els C, Kung JY, Straube S. Mindfulness-based practices in workers to address mental health conditions: A systematic review. *Safety Health Work* 2023; 14: 250-8.
221. Dupont F, Léger P, Begon M, et al. Health and productivity at work: which active workstation for which benefits: a systematic review. *Occup Environ Med* 2019; 76: 281-94.
222. Elbers S, Wittink H, Konings S, et al. Longitudinal outcome evaluations of interdisciplinary multimodal pain treatment programmes for patients with chronic primary musculoskeletal pain: A systematic review and meta-analysis. *Eur J Pain* 2022; 26: 310-35. [no estimates for workplaces]
223. Emerson LM, Leyland A, Hudson K, Rowse G, Hanley P, Hugh-Jones S. Teaching mindfulness to teachers: a systematic review and narrative synthesis. *Mindfulness* 2017; 8: 1136-49.
224. Feltner C, Peterson K, Palmieri Weber R, et al. The effectiveness of Total Worker Health interventions: A systematic review for a National Institutes of Health Pathways to Prevention workshop. *Ann Int Med* 2016; 165: 4.
225. Fernandez ID, Becerra A, Chin NP. Worksite environmental interventions for obesity prevention and control: Evidence from group randomized trials. *Curr Obes Rep* 2014; 3: 223-34.

226. Fibbins H, Ward PB, Watkins A, Curtis J, Rosenbaum S. Improving the health of mental health staff through exercise interventions: a systematic review. *J Ment Health* 2018; 27: 184-91.
227. Fox S, Lydon S, Byrne D, Madden C, Connolly F, O'Connor P. A systematic review of interventions to foster physician resilience. *Postgrad Med J* 2018; 94: 162-70.
228. Frascella B, Oradini-Alacreu A, Balzarini F, Signorelli C, Lopalco PL, Odone A. Effectiveness of email-based reminders to increase vaccine uptake: a systematic review. *Vaccine* 2020; 38: 433-43.
229. Gardner B, Smith L, Lorencatto F, Hamer M, Biddle SJ. How to reduce sitting time? A review of behaviour change strategies used in sedentary behaviour reduction interventions among adults. *Health Psychol Rev* 2016; 10: 89-112.
230. Gari D, Alabdulhadi AA, Alahmari AA, Alsalman ZA, Alshehr HS. Addressing occupational back pain: A systematic review of preventive and therapeutic strategies. *Cureus* 2023; 15: e48744.
231. Gatwood J, Meltzer MI, Messonnier M, Ortega-Sanchez IR, Balkrishnan R, Prosser LA. Seasonal influenza vaccination of healthy working-age adults: a review of economic evaluations. *Drugs* 2012; 72: 35-48.
232. Gawlik A, Ludemann J, Neuhausen A, Zepp C, Vitinius F, Kleinert J. A systematic review of workplace physical activity coaching. *J Occup Rehabil* 2023; 33: 550-69.
233. Geaney F, Kelly C, Greiner BA, Harrington JM, Perry IJ, Beirne P. The effectiveness of workplace dietary modification interventions: a systematic review. *Prev Med* 2013; 57: 438-47.
234. Gebhard D, Herz M. How to address the health of home care workers: A systematic review of the last two decades. *J Appl Gerontol* 2023; 42: 689-703.
235. Ghawadra SF, Abdullah KL, Choo WY, Phang CK. Mindfulness-based stress reduction for psychological distress among nurses: A systematic review. *J Clin Nurs* 2019; 28: 3747-58.
236. Ghobadi K, Eslami A, Pirzadeh A, Mazloomi SM, Hosseini F. Effects of the nutritional interventions in improving employee's cardiometabolic risk factors in the workplace: A systematic review. *Clin Nutr Open Science* 2022; 42: 73-83.
237. Gilmartin H, Goyal A, Hamati MC, Mann J, Saint S, Chopra V. Brief mindfulness practices for healthcare providers - A systematic literature review. *Am J Med* 2017; 130: 1219 e1- e17.
238. Goodman G, Kovach L, Fisher A, Elsesser E, Bobinski D, Hansen J. Effective interventions for cumulative trauma disorders of the upper extremity in computer users: practice models based on systematic review. *Work* 2012; 42: 153-72.
239. Grimani A, Aboagye E, Kwak L. The effectiveness of workplace nutrition and physical activity interventions in improving productivity, work performance and workability: a systematic review. *BMC Public Health* 2019; 19: 1676.
240. Groeneveld IF, Proper KI, van der Beek AJ, Hildebrandt VH, van Mechelen W. Lifestyle-focused interventions at the workplace to reduce the risk of cardiovascular disease - a systematic review. *Scand J Work Environ Health* 2010; 36: 202-15.
241. Gudzune K, Hutfless S, Maruthur N, Wilson R, Segal J. Strategies to prevent weight gain in workplace and college settings: a systematic review. *Prev Med* 2013; 57: 268-77.
242. Hartmann S, Weiss M, Newman A, Hoegl M. Resilience in the workplace: A multilevel review and synthesis. *Appl Psychol* 2020; 69: 913-59.
243. Hartmann-Boyce J, Lindson N, Butler AR, et al. Electronic cigarettes for smoking cessation. *Cochrane Database Syst Rev* 2022; 11: CD010216.
244. Heijkants CH, de Wind A, van Hooff MLM, Geurts SAE, Boot CRL. Effectiveness of team and organisational level workplace interventions aimed at improving sustainable employability of aged care staff: A systematic review. *J Occup Rehabil* 2023; 33: 37-60.
245. Hendren S, Logomarsino J. Impact of worksite cafeteria interventions on fruit and vegetable consumption in adults - A systematic review. *Int J Workplace Health Manag* 2017; 10: 134-52.
246. Henshall C, Ostinelli E, Harvey J, et al. Examining the effectiveness of web-based interventions to enhance resilience in health care professionals: Systematic review. *JMIR Med Educ* 2022; 8: e34230.
247. Heuel L, Otto A, Wollesen B. Physical exercise and ergonomic workplace interventions for nursing personnel—effects on physical and mental health: a systematic review. *Ger J Exerc Sport Res* 2024; 54: 291-324.

248. Hidajat TJ, Edwards EJ, Wood R, Campbell M. Mindfulness-based interventions for stress and burnout in teachers: A systematic review. *Teaching Teacher Educ* 2023; 134: 104303.
249. Hill A, Alston L, Needham C, Peeters A, LaMontagne AD, Nichols M. Systematic review of the effectiveness of health promotion interventions targeting obesity prevention in school-based staff. *Health Prom Int* 2022; 37: 3.
250. Hoosain M, de Klerk S, Burger M. Workplace-based rehabilitation of upper limb conditions: A systematic review. *J Occup Rehabil* 2019; 29: 175-93.
251. Howarth A, Quesada J, Silva J, Judycki S, Mills PR. The impact of digital health interventions on health-related outcomes in the workplace: A systematic review. *Digit Health* 2018; 4: 2055207618770861.
252. Hutcheson AK, Piazza AJ, Knowlden AP. Work site-based environmental interventions to reduce sedentary behavior: A systematic review. *Am J Health Promot* 2018; 32: 32-47.
253. Hwang Y, Bartlett B, Greben M, Hand K. A systematic review of mindfulness interventions for in-service teachers: A tool to enhance teacher wellbeing and performance. *Teaching Teacher Educ* 2017; 64: 26-42.
254. Häggman-Laitila A, Romppanen J. Outcomes of interventions for nurse leaders' well-being at work: A quantitative systematic review. *J Adv Nurs* 2018; 74: 34-44.
255. Hogg B, Medina JC, Gardoki-Souto I, et al. Workplace interventions to reduce depression and anxiety in small and medium-sized enterprises: A systematic review. *J Affect Disord* 2021; 290: 378-86.
256. Ihara Y, Kurosawa T, Matsumoto T, Takizawa R. The effectiveness of preventive group cognitive-behavioral interventions on enhancing work performance-related factors and mental health of workers: a systematic review. *Curr Psychol* 2023; 42: 2797–810.
257. Indra B, Palmasutra V, Setyawan FA. Effectiveness of digital Interventions in reducing occupational stress: A Systematic Review. *Portug J Public Health* 2024; 42: 252-65.
258. Inolopu J, Hilario-Huapayal N, Tantalean-Del-Águila MA, Hurtado-Roca Y, Ugarte-Gill C. Interventions for the prevention of risk factors and incidence of type 2 diabetes in the work environment: a systematic review. *Revista Saude Publ* 2019; 53: 101.
259. Ivandic I, Freeman A, Birner U, Nowak D, Sabariego C. A systematic review of brief mental health and well-being interventions in organizational settings. *Scand J Work Environ Health* 2017; 43: 99-108.
260. Janssen M, Heerkens Y, Kuijer W, van der Heijden B, Engels J. Effects of mindfulness-based stress reduction on employees' mental health: A systematic review. *PLoS One* 2018; 13: e0191332.
261. Jensen JD. Can worksite nutritional interventions improve productivity and firm profitability? A literature review. *Persp Public Health* 2011; 131: 184-92.
262. Jiménez O, Ramos NS, González-Moraleda A, Resurrección DM. Brief mindfulness-based interventions in a laboratory context: a systematic review of randomized controlled trials. *Mindfulness* 2020; 11: 849-61.
263. Jimenez-Merida MR, Romero-Saldana M, Molina-Luque R, et al. Women-centred workplace health promotion interventions: a systematic review. *Int Nurs Rev* 2021; 68: 90-8.
264. Joseph A, Jose TP. Coping with distress and building resilience among emergency nurses: A systematic review of mindfulness-based interventions. *Indian J Crit Care Med* 2024; 28: 785-91.
265. Kahn-Marshall JL, Gallant MP. Making healthy behaviors the easy choice for employees: a review of the literature on environmental and policy changes in worksite health promotion. *Health Educ Behav* 2012; 39: 752-76.
266. Kelly M, Wills J. Systematic review: What works to address obesity in nurses? *Occup Med* 2018; 68: 228-38.
267. Klein A, Taieb O, Xavier S, Baubet T, Reyre A. The benefits of mindfulness-based interventions on burnout among health professionals: A systematic review. *Explore* 2020; 16: 35-43.
268. Knowlden AP, Ickes MJ, Sharma M. Systematic analysis of tobacco treatment interventions implemented in worksite settings. *J Subst Use* 2014; 19: 283-94.
269. Komase Y, Watanabe K, Hori D, et al. Effects of gratitude intervention on mental health and well-being among workers: A systematic review. *J Occup Health* 2021; 63: e12290.

270. Krungkraipetch N, Krungkraipetch K, Kaewboonchoo O, Arphorn S, Sim M. Interventions to prevent musculoskeletal disorders among informal sector workers: a literature review. *Southeast Asian J Trop Med Public Health* 2012; 43: 510-25.
271. Kriakous SA, Elliott KA, Lamers C, Owen R. The effectiveness of mindfulness-based stress reduction on the psychological functioning of healthcare professionals: a systematic review. *Mindfulness* 2021; 12: 1-28.
272. Krishnan A, Odejimi O, Bertram I, Chukowry PS, Tadros G. A systematic review of interventions aiming to improve newly-qualified doctors' wellbeing in the United Kingdom. *BMC Psychol* 2022; 10: 161.
273. Kullen C, Mitchell L, O'Connor HT, Gifford JA, Beck KL. Effectiveness of nutrition interventions on improving diet quality and nutrition knowledge in military populations: a systematic review. *Nutr Rev* 2022; 80: 1664-93.
274. Kuster AT, Dalsbo TK, Luong Thanh BY, Agarwal A, Durand-Moreau QV, Kirkehei I. Computer-based versus in-person interventions for preventing and reducing stress in workers. *Cochrane Database Syst Rev* 2017; 8: CD011899.
275. Laiou E, Rapti I, Schwarzer R, et al. Review: Nudge interventions to promote healthy diets and physical activity. *Food Policy* 2021; 102: 102103.
276. Larinier N, Vuillerme N, Balaguier R. Effectiveness of warm-up interventions on work-related musculoskeletal disorders, physical and psychosocial functions among workers: a systematic review. *BMJ open* 2023; 13: e056560.
277. Lassen AD, Fagt S, Lennernas M, et al. The impact of worksite interventions promoting healthier food and/or physical activity habits among employees working 'around the clock' hours: a systematic review. *Food Nutr Res* 2018; 62.
278. Lee NK, Roche A, Duraisingam V, Fischer JA, Cameron J. Effective interventions for mental health in male-dominated workplaces. *Mental Health Rev J* 2014; 19: 237-50.
279. Lee Y, Lee NY, Lim HJ, Sung S. Weight reduction interventions using digital health for employees with obesity: A systematic Review. *Diabetes Metab Syndr Obes* 2022; 15: 3121-31.
280. Lees T, Elliott JL, Gunning S, Newton PJ, Rai T, Lal S. A systematic review of the current evidence regarding interventions for anxiety, PTSD, sleepiness and fatigue in the law enforcement workplace. *Ind Health* 2019; 57: 655-67.
281. Li X, Zhou Y, Yuen KF. A systematic review on seafarer health: Conditions, antecedents and interventions. *Transport Policy* 2022; 122: 11-25.
282. Litwan K, Tran V, Nyhan K, Perez-Escamilla R. How do breastfeeding workplace interventions work?: a realist review. *Int J Equity Health* 2021; 20: 148.
283. Locke R, Lees A. A literature review of interventions to reduce stress in doctors. *Persp Public Health* 2020; 140: 38-53.
284. Lopez-Del-Hoyo Y, Fernandez-Martinez S, Perez-Aranda A, et al. Effects of eHealth interventions on stress reduction and mental health promotion in healthcare professionals: A systematic review. *J Clin Nurs* 2023; 32: 5514-33.
285. Lowe BD, Dick RB. Workplace exercise for control of occupational neck/shoulder disorders: a review of prospective studies. *Environ Health Insights* 2014; 8: 75-95.
286. Luken M, Sammons A. systematic review of mindfulness practice for reducing job burnout. *Am J Occup Ther* 2016; 70: 7002250020p1-p10.
287. MacMillan F, Karamacoska D, El Masri A, et al. A systematic review of health promotion intervention studies in the police force: study characteristics, intervention design and impacts on health. *Occup Environ Med* 2017; 74: 913-23.
288. MacMillan F, Kolt GS, Le A, George ES. Systematic review of randomised control trial health promotion intervention studies in the fire services: study characteristics, intervention design and impacts on health. *Occup Environ Med* 2020; oemed-2020-106613.
289. Madden SK, Cordon EL, Bailey C, et al. The effect of workplace lifestyle programmes on diet, physical activity, and weight-related outcomes for working women: A systematic review using the TIDieR checklist. *Obes Rev* 2020; 21: e13027.

290. Malkawi AM, Meertens RM, Kremers SPJ, Sleddens EFC. Dietary, physical activity, and weight management interventions among active-duty military personnel: a systematic review. *Mil Med Res* 2018; 5: 43.
291. Maes L, Van Cauwenberghe E, Van Lippevelde W, et al. Effectiveness of workplace interventions in Europe promoting healthy eating: a systematic review. *Eur J Public Health* 2012; 22: 677-83.
292. Malik SH, Blake H, Suggs LS. A systematic review of workplace health promotion interventions for increasing physical activity. *Br J Health Psychol* 2014; 19: 149-80.
293. Marin TJ, Van Eerd D, Irvin E, et al. Multidisciplinary biopsychosocial rehabilitation for subacute low back pain. *Cochrane Database Syst Rev* 2017; 6: CD002193.
294. Marin-Farrona M, Wipfli B, Thosar SS, et al. Effectiveness of worksite wellness programs based on physical activity to improve workers' health and productivity: a systematic review. *Syst Rev* 2023; 12: 87.
295. Martland RN, Ma R, Paleri V, et al. The efficacy of physical activity to improve the mental wellbeing of healthcare workers: A systematic review. *Mental Health Phys Act* 2024; 26: 100577.
296. McCoy K, Stinson K, Scott K, Tenney L, Newman LS. Health promotion in small business: a systematic review of factors influencing adoption and effectiveness of worksite wellness programs. *J Occup Environ Med* 2014; 56: 579-87.
297. Mehta S, Dimsdale J, Nagle B, et al. Worksite interventions: improving lifestyle habits among Latin American adults. *Am J Prev Med* 2013; 44: 538-42.
298. Mejsner SB, Baygi F, Timilsina A, et al. Perspectives on empowerment programs, and interventions in maritime settings: A systematic review. *J Transport Health* 2024; 36: 101816.
299. Melnyk BM, Kelly SA, Stephens J, et al. Interventions to improve mental health, well-being, physical health, and lifestyle behaviors in physicians and nurses: A systematic review. *Am J Health Promot* 2020; 34: 929-41.
300. Meng L, Wolff MB, Mattick KA, DeJoy DM, Wilson MG, Smith ML. Strategies for worksite health interventions to employees with elevated risk of chronic diseases. *Safety Health Work* 2017; 8: 117-29.
301. Michalchuk VF, Lee S, Waters CM, Hong OS, Fukuoka Y. Systematic review of the influence of physical work environment on office workers' physical activity behavior. *Workplace Health Safety* 2022; 70: 97-119.
302. Moore C, Kelly S, Melnyk BM. The use of mHealth apps to improve hospital nurses' mental health and well-being: A systematic review. *Worldviews Evid Based Nurs* 2024; 21: 110-9.
303. Moorfield C, Cope V. Interventions to increase resilience in physicians: A structured literature review. *Explore* 2020; 16: 103-9.
304. Moreno AF, Karanika-Murray M, Batista P, Hill R, Vilalta SR, Oliveira-Silva P. Resilience training programs with police forces: A systematic review. *J Pol Crim Psychol* 2024; 39: 227-52.
305. Morrow A, Walker K, Calder-MacPhee N, Ozakinci G. The active ingredients of physical activity and / or dietary workplace-based interventions to achieve weight loss in overweight and obese healthcare staff: a systematic review. *J Behav Med* 2022; 45: 331-49.
306. Muir SD, Silva SSM, Woldegiorgis MA, Rider H, Meyer D, Jayawardana MW. Predictors of success of workplace physical activity interventions: A systematic review. *J Physical Act Health* 2019; 16: 647-56.
307. Mulimani P, Hoe VC, Hayes MJ, Idiculla JJ, Abas AB, Karanth L. Ergonomic interventions for preventing musculoskeletal disorders in dental care practitioners. *Cochrane Database Syst Rev* 2018; 10: CD011261.
308. Murray M, Murray L, Donnelly M. Systematic review of interventions to improve the psychological well-being of general practitioners. *BMC Fam Pract* 2016; 17: 36.
309. Murphy B, Parekh N, Vieira DL, O'Connor JA. A systematic review of randomized controlled trials examining workplace wellness interventions. *Nutr Health* 2022; 28: 111-22.
310. Manttari S, Oksa J, Lusa S, et al. Interventions to promote work ability by increasing physical activity among workers with physically strenuous jobs: A scoping review. *Scand J Public Health* 2021; 49: 206-18.

311. Naehrig D, Schokman A, Hughes JK, Epstein R, Hickie IB, Glozier N. Effect of interventions for the well-being, satisfaction and flourishing of general practitioners-a systematic review. *BMJ Open* 2021; 11: e046599.
312. Naicker A, Shrestha A, Joshi C, Willett W, Spiegelman D. Workplace cafeteria and other multicomponent interventions to promote healthy eating among adults: A systematic review. *Prev Med Rep* 2021; 22: 101333.
313. Najafabadi ZA, Vaezi A, Tavakolifard N, Amini Z. The effectiveness of implemented interventions at the workplace to promote the mental health of working women: A systematic review. *J Educ Health Promot* 2024; 13: 496.
314. Neil-Sztramko SE, Pahwa M, Demers PA, Gotay CC. Health-related interventions among night shift workers: a critical review of the literature. *Scand J Work Environ Health* 2014; 40: 543-56.
315. Nestler K, Witzki A, Rohde U, Ruther T, Tofaute KA, Leyk D. Strength training for women as a vehicle for health promotion at work. *Deutsch Arzteblatt Int* 2017; 114: 439-46.
316. Ng MK, ousuf B, Bigelow PL, Van Eerd D. Effectiveness of health promotion programmes for truck drivers: A systematic review. *Health Educ J* 2015; 74: 270-86.
317. Ni Mhurchu C, Aston LM, Jebb SA. Effects of worksite health promotion interventions on employee diets: a systematic review. *BMC Public Health* 2010; 10: 62.
318. Oakman J, Keegel T, Kinsman N, Briggs AM. Persistent musculoskeletal pain and productive employment; a systematic review of interventions. *Occup Environ Med* 2016; 73: 206-14.
319. Osilla KC, Van Busum K, Schnyer C, Larkin JW, Eibner C, Mattke S. Systematic review of the impact of worksite wellness programs. *Am J Manag Care* 2012; 18: e68-81.
320. Ottisova L, Gillard JA, Wood M, et al. Effectiveness of psychosocial interventions in mitigating adverse mental health outcomes among disaster-exposed health care workers: A systematic review. *J Trauma Stress* 2022; 35: 746-58.
321. Otto AK, Gutsch C, Bischoff LL, Wollesen B. Interventions to promote physical and mental health of nurses in elderly care: A systematic review. *Prev Med* 2021; 148: 106591.
322. Paganin G, Simbula S. Smartphone-based interventions for employees' well-being promotion: a systematic review. *Electr J Appl Stat Anal* 2020; 13: 682-712.
323. Palmer KT, Harris EC, Linaker C, et al. Effectiveness of community- and workplace-based interventions to manage musculoskeletal-related sickness absence and job loss: a systematic review. *Rheumatol* 2012; 51: 230-42.
324. Park S, Jang MK. Associations between workplace exercise interventions and job stress reduction: A systematic review. *Workplace Health Saf* 2019; 67: 592-601.
325. Park JH, Jung SE, Ha DJ, et al. The effectiveness of e-healthcare interventions for mental health of nurses: A PRISMA-compliant systematic review of randomized controlled trials. *Medicine* 2022; 101: e29125.
326. Parreira P, Heymans MW, van Tulder MW, et al. Back Schools for chronic non-specific low back pain. *Cochrane Database Syst Rev* 2017; 8: CD011674.
327. Patterson PD, Liszka MK, McIlvaine QS, et al. Does the evidence support brief (</=30-mins), moderate (31-60-mins), or long duration naps (61+ mins) on the night shift? A systematic review. *Sleep Med Rev* 2021; 59: 101509.
328. Peters M, Klein T, Stuber F, et al. Moderators and mediators of effects of interventions to reduce stress in hospital employees: A systematic review. *Stress Health* 2024; 40: e3314.
329. Petrunoff N, Rissel C, Wen LM. The effect of active travel interventions conducted in work settings on driving to work: A systematic review. *J Transport Health* 2016; 3: 61-76.
330. Pham CT, Phung D, Nguyen TV, Chu C. The effectiveness of workplace health promotion in low- and middle-income countries. *Health Prom Int* 2020; 35: 1220-9.
331. Phillips CS, Becker H. Systematic review: Expressive arts interventions to address psychosocial stress in healthcare workers. *J Adv Nurs* 2019; 75: 2285-98.
332. Plotnikoff R, Collins CE, Williams R, Germov J, Callister R. Effectiveness of interventions targeting health behaviors in university and college staff: a systematic review. *Am J Health Promot* 2015; 29: e169-87.

333. Porter C, Lommen MJJ. Primary prevention of trauma-related disorders in high-risk professionals: A systematic review. *Mental Health Prev* 2023; 29: 200257.
334. Poscia A, Moscato U, La Milia DI, et al. Workplace health promotion for older workers: a systematic literature review. *BMC Health Serv Res* 2016; 16 Suppl 5: 329.
335. Pritchard EK, Kim HC, Nguyen N, van Vreden C, Xia T, Iles R. The effect of weight loss interventions in truck drivers: Systematic review. *PLoS One* 2022; 17: e0262893.
336. Radwan A, Barnes L, DeResh R, Englund C, Gribanoff S. Effects of active microbreaks on the physical and mental well-being of office workers: A systematic review. *Cogent Eng* 2022; 9: 2026206.
337. Ramezani M, Tayefi B, Zandian E, et al. Workplace interventions for increasing physical activity in employees: A systematic review. *J Occup Health* 2022; 64: e12358.
338. Redeker NS, Caruso CC, Hashmi SD, Mullington JM, Grandner M, Morgenthaler TI. Workplace interventions to promote sleep health and an alert, healthy workforce. *J Clin Sleep Med* 2019; 15: 649-57.
339. L.L.R. R, Kuebler JC. The behavior of pain in response to sit-stand workstations: a systematic review. *Phys Ther Rev* 2019; 24: 223-8.
340. Richardson A, McNoe B, Derrett S, Harcombe H. Interventions to prevent and reduce the impact of musculoskeletal injuries among nurses: A systematic review. *Int J Nurs Stud* 2018; 82: 58-67.
341. Richter K, Acker J, Adam S, Niklewski G. Prevention of fatigue and insomnia in shift workers-a review of non-pharmacological measures. *EPMA J* 2016; 7: 16.
342. Riva S, Chinyio E. Stress factors and stress management interventions: the heuristic of “bottom up” an update from a systematic review. *Occup Health Sci* 2018; 2: 127-55.
343. Robbins R, Underwood P, Jackson CL, et al. A systematic review of workplace-based employee health interventions and their impact on sleep duration among shift workers. *Workplace Health Saf* 2021; 69: 525-39.
344. Robertson IT, Cooper CL, Sarkar M, Curran T. Resilience training in the workplace from 2003 to 2014: A systematic review. *J Occup Organ Psychol* 2015; 88: 533-62.
345. Rogers D. Which educational interventions improve healthcare professionals' resilience? *Med Teach* 2016; 38: 1236-41.
346. Roll SC, Tung KD, Chang H, et al. Prevention and rehabilitation of musculoskeletal disorders in dental professionals: A systematic review. *J Am Dent Assoc* 2019; 150: 489-502.
347. Romppanen J, Haggman-Laitila A. Interventions for nurses' well-being at work: a quantitative systematic review. *J Adv Nurs* 2017; 73: 1555-69.
348. Rostami M, Rahmati-Najarkolaei F, Salesi M, Azad E. A systematic review of suicide prevention interventions in military personnel. *Arch Suicide Res* 2022; 26: 481-99.
349. Ryan JC, Williams G, Wiggins BW, et al. Exploring the active ingredients of workplace physical and psychological wellbeing programs: a systematic review. *Transl Behav Med* 2021; 11: 1127-41.
350. Sahabudhee A, Rao CR, Chandrasekaran B, Pedersen SJ. Dose-response effects of periodic physical activity breaks on the chronic inflammatory risk associated with sedentary behavior in high- and upper-middle income countries: A systematic review and meta-analysis. *Diabetes Metab Syndr* 2023; 17: 102730.
351. Sanderson PW, Clemes SA, Biddle SJ. The correlates and treatment of obesity in military populations: a systematic review. *Obes Facts* 2011; 4: 229-37.
352. Sawada K, Wada K, Shahrook S, Ota E, Takemi Y, Mori R. Social marketing including financial incentive programs at worksite cafeterias for preventing obesity: a systematic review. *Syst Rev* 2019; 8: 66.
353. Scheepers RA, Emke H, Epstein RM, Lombarts K. The impact of mindfulness-based interventions on doctors' well-being and performance: A systematic review. *Med Educ* 2020; 54: 138-49.
354. Sepehrian R, Aghaei Hashjin A, Farahmandnia H. A systematic review of programs and interventions for reduction of sickness absence in nursing staff with work-related musculoskeletal disorders. *J Educ Health Promot* 2024; 13: 205.
355. Sevic A, Hashemi NS, Thorrisen MM, et al. Effectiveness of eHealth iInterventions targeting employee health behaviors: Systematic review. *J Med Internet Res* 2023; 25: e38307.

356. Sidossis A, Gaviola GC, Sotos-Prieto M, Kales S. Healthy lifestyle interventions across diverse workplaces: a summary of the current evidence. *Curr Opin Clin Nutr Metab Care* 2021; 24: 490-503.
357. Silva H, Ramos PGF, Teno SC, Judice PB. The impact of sit-stand desks on full-day and work-based sedentary behavior of office workers: A systematic review. *Hum Factors* 2024: 187208241305591.
358. Skamagki G, King A, Duncan M, Wahlin C. A systematic review on workplace interventions to manage chronic musculoskeletal conditions. *Physiother Res Int* 2018; 23: e1738.
359. Soler RE, Leeks KD, Razi S, et al. A systematic review of selected interventions for worksite health promotion. The assessment of health risks with feedback. *Am J Prev Med* 2010; 38: S237-62.
360. Soprovich AL, Seaton CL, Bottorff JL, et al. A systematic review of workplace behavioral interventions to promote sleep health in men. *Sleep Health* 2020; 6: 418-30.
361. Stapelfeldt CM, Klaver KM, Rosbjerg RS, et al. A systematic review of interventions to retain chronically ill occupationally active employees in work: can findings be transferred to cancer survivors? *Acta Oncol* 2019; 58: 548-65.
362. Stock SR, Nicolakakis N, Vezina N, et al. Are work organization interventions effective in preventing or reducing work-related musculoskeletal disorders? A systematic review of the literature. *Scand J Work Environ Health* 2018; 44: 113-33.
363. Suleiman-Martos N, Gomez-Urquiza JL, Aguayo-Estremera R, Canadas-De La Fuente GA, De La Fuente-Solana EI, Albendin-Garcia L. The effect of mindfulness training on burnout syndrome in nursing: A systematic review and meta-analysis. *J Adv Nurs* 2020; 76: 1124-40.(no relevant meta-analysis)
364. Sulosaari V, Unal E, Cinar FI. The effectiveness of mindfulness-based interventions on the psychological well-being of nurses: A systematic review. *Appl Nurs Res* 2022; 64: 151565.
365. Sundstrup E, Seeberg KGV, Bengtsen E, Andersen LL. A systematic review of workplace interventions to rehabilitate musculoskeletal disorders among employees with physical demanding work. *J Occup Rehabil* 2020; 30: 588-612.
366. Sundstrup E, Seeberg KGV, Dyreborg J, Clausen T, Andersen LL. Systematic review of workplace interventions to support young workers' safety, work environment and health. *J Occup Rehabil* 2024; epub.
367. Sweeney K, Mackey M, Spurway J, Clarke J, Ginn K. The effectiveness of ergonomics interventions in reducing upper limb work-related musculoskeletal pain and dysfunction in sonographers, surgeons and dentists: a systematic review. *Ergonomics* 2021; 64: 1-38.
368. Swinton PA, Cooper K, Hancock E. Workplace interventions to improve sitting posture: A systematic review. *Prev Med* 2017; 101: 204-12.
369. Tam G, Yeung MPS. A systematic review of the long-term effectiveness of work-based lifestyle interventions to tackle overweight and obesity. *Prev Med* 2018; 107: 54-60.
370. Tersa-Miralles C, Bravo C, Bellon F, Pastells-Peiro R, Rubinat Arnaldo E, Rubi-Carnacea F. Effectiveness of workplace exercise interventions in the treatment of musculoskeletal disorders in office workers: a systematic review. *BMJ open* 2022; 12: e054288.
371. Tew GA, Posso MC, Arundel CE, McDaid CM. Systematic review: height-adjustable workstations to reduce sedentary behaviour in office-based workers. *Occup Med* 2015; 65: 357-66.
372. Tjasink M, Keiller E, Stephens M, Carr CE, Priebe S. Art therapy-based interventions to address burnout and psychosocial distress in healthcare workers—a systematic review. *BMC Health Serv Res* 2018; 23: 1059.
373. To QG, Chen TT, Magnussen CG, To KG. Workplace physical activity interventions: a systematic review. *Am J Health Promot* 2013; 27: e113-23.
374. Torquati L, Pavey T, Kolbe-Alexander T, Leveritt M. Promoting diet and physical activity in nurses. *Am J Health Promot* 2017; 31: 19-27.
375. Townsley AP, Li-Wang J, Katta R. Healthcare workers' well-being: A systematic review of positive psychology interventions. *Cureus* 2023; 15: e34102.
376. Tuckwell GA, Vincent GE, Gupta CC, Ferguson SA. Does breaking up sitting in office-based settings result in cognitive performance improvements which last throughout the day? A review of the evidence. *Ind Health* 2022; 60: 501-13.

377. Unjai S, Forster EM, Mitchell AE, Creedy DK. Interventions to promote resilience and passion for work in health settings: A mixed-methods systematic review. *Int J Nurs Stud Adv* 2024; 7: 100242.
378. Vadvilavicius T, Varnagiryte E, Jarasiunaite-Fedosejeva G, Gustainiene L. The effectiveness of mindfulness-based interventions for police officers' stress reduction: a systematic review. *J Police Crim Psychol* 2023; 38: 223-39.
379. van de Ven D, Robroek SJW, Burdorf A. Are workplace health promotion programmes effective for all socioeconomic groups? A systematic review. *Occup Environ Med* 2020; 77: 589-96.
380. Van Eerd D, Munhall C, Irvin E, et al. Effectiveness of workplace interventions in the prevention of upper extremity musculoskeletal disorders and symptoms: an update of the evidence. *Occup Environ Med* 2016; 73: 62-70.
381. Van Hoof W, O'Sullivan K, O'Keeffe M, Verschueren S, O'Sullivan P, Dankaerts W. The efficacy of interventions for low back pain in nurses: A systematic review. *Int J Nurs Stud* 2018; 77: 222-31.
382. van Niekerk SM, Louw QA, Hillier S. The effectiveness of a chair intervention in the workplace to reduce musculoskeletal symptoms. A systematic review. *BMC Musculoskelet Disord* 2012; 13: 145.
383. Varatharajan S, Cote P, Shearer HM, et al. Are work disability prevention interventions effective for the management of neck pain or upper extremity disorders? A systematic review by the Ontario Protocol for Traffic Injury Management (OPTIMa) collaboration. *J Occup Rehabil* 2014; 24: 692-708.
384. Venegas CL, Nkangu MN, Duffy MC, Fergusson DA, Spilg EG. Interventions to improve resilience in physicians who have completed training: A systematic review. *PLoS One* 2019; 14: e0210512.
385. Vilar-Compte M, Hernández-Cordero S, Ancira-Moreno M, et al. Breastfeeding at the workplace: a systematic review of interventions to improve workplace environments to facilitate breastfeeding among working women. *Int J Equity Health* 2021; 20: 110.
386. Vitzthum MA, Kruger K, Weyh C. The impact of financial incentives on physical activity for employees in the context of workplace health promotion: a systematic review. *J Occup Health* 2024; 66: 1.
387. Vuillemin A, Rostami C, Maes L, et al. Worksite physical activity interventions and obesity: a review of European studies (the HOPE project). *Obes Facts* 2011; 4: 479-88.
388. Wan Mohd Yunus WMA, Musiat P, Brown JSL. Systematic review of universal and targeted workplace interventions for depression. *Occup Environ Med* 2018; 75: 66-75.
389. Wang Y, Wub L, Lange J, Fadhild A, Reiterera H. Persuasive technology in reducing prolonged sedentary behavior at work: A systematic review. *Smart Health* 2018; 7-8: 19-30.
390. Waongenngarm P, Areerak K, Janwantanakul P. The effects of breaks on low back pain, discomfort, and work productivity in office workers: A systematic review of randomized and non-randomized controlled trials. *Appl Ergon* 2018; 68: 230-9.
391. Weerasekara YK, Roberts SB, Kahn MA, LaVertu AE, Hoffman B, Das SK. Effectiveness of workplace weight management interventions: a systematic review. *Curr Obes Rep* 2016; 5: 298-306.
392. Wegrzynek PA, Wainwright E, Ravalier J. Return to work interventions for chronic pain: a systematic review. *Occup Med* 2020; 70: 268-77.
393. Westermann C, Kozak A, Harling M, Nienhaus A. Burnout intervention studies for inpatient elderly care nursing staff: systematic literature review. *Int J Nurs Stud* 2014; 51: 63-71.
394. Wiederhold BK, Cipresso P, Pizzioli D, Wiederhold M, Riva G. Intervention for physician burnout: A systematic review. *Open Med* 2018; 13: 253-63.
395. Wiisak J, Suikkala A, Leino-Kilpi H, Stolt M, Suhonen R, Koskinen S. Interventions intended to improve the well-being at work of nurses working in care settings for older people - A systematic review. *Int J Older People Nurs* 2025; 20: e70005.
396. Wild J, El-Salahi S, Esposti MD. The effectiveness of interventions aimed at improving well-being and resilience to stress in first responders - A systematic review. *Eur Psychol* 2020; 25: 252-71.
397. Williams SP, Malik HT, Nicolay CR, Chaturvedi S, Darzi A, Purkayastha S. Interventions to improve employee health and well-being within health care organizations: A systematic review. *Am Soc Healthcare Risk Manag* 2017; 37: 25-51.
398. Wolkow A, Netto K, Aisbett B. The effectiveness of health interventions in cardiovascular risk reduction among emergency service personnel. *Int Arch Occup Environ Health* 2013; 86: 245-60.

399. Wong JY, Gilson ND, van Uffelen JG, Brown WJ. The effects of workplace physical activity interventions in men: a systematic review. *Am J Men Health* 2012; 6: 303-13.
400. Wu J, Li H, Shuai J, He Y, Li P. Evidence summary on the non-pharmacological management of sleep disorders in shift workers. *Sleep Breath* 2024; 28: 909-18.
401. Xu HG, Kynoch K, Tuckett A, Eley R. Effectiveness of interventions to reduce emergency department staff occupational stress and/or burnout: a systematic review. *JBI Evid Synth* 2020; 18: 1156-88.
402. Zacharia S, Funk M, Alshuwaiyer G, Gwin S, Taylor EL, Branscum P. Internet-based physical activity interventions at the worksite: A systematic review. *Am J Health Stud* 2013; 28: 114-26.
403. Selič-Zupančič P, Klemenc-Ketiš Z, Tement SO. The impact of psychological interventions with elements of mindfulness on burnout and well-being in healthcare rofessionals: A systematic review. *J Multidisc Healthcare* 2023; 16: 1821-31.
404. Zheng C, Zhang X, Sheridan S, et al. Effect of sedentary behavior interventions on vascular function in adults: A systematic review and meta-analysis. *Scand J Med Sci Sports* 2021; 31: 1395-410.
405. Zhu X, Yoshikawa A, Qiu L, Luy Z, Lee C, Ory M. Healthy workplaces, active employees: A systematic literature review on impacts of workplace environments on employees' physical activity and sedentary behavior. *Build Environ* 2020; 168: 106455.
